# Nonlinear impacts of climate anomalies on oil palm productivity

**DOI:** 10.1016/j.heliyon.2024.e35798

**Published:** 2024-08-06

**Authors:** Nur Nadia Kamil, Saizi Xiao, Sharifah Nabilah Syed Salleh, Hongbing Xu, Castiel Chen Zhuang

**Affiliations:** a Economics and Industry Development Division, Malaysian Palm Oil Board, Selangor, Malaysia; b School of Economics, University of Nottingham Malaysia, Selangor, Malaysia; c Department of Economics, Kulliyyah of Economics and Management Sciences, International Islamic University Malaysia, Kuala Lumpur, Malaysia; d Department of Occupational and Environmental Health, Peking University School of Public Health, Beijing, China; e Peking University School of Economics, Beijing, China

## Abstract

Oil palm contributes to various global needs as one of the most productive oil crops, but there exist ongoing concerns regarding its yield reductions and associated environmental impacts resulting from land conversion. This is the first detailed report investigating the nonlinear threats to estate-level oil palm yields posed by El Niño Southern Oscillation (ENSO) in the equatorial Pacific Ocean, a major driver of climate variability. Using the Malaysian Palm Oil Board administrative records on monthly performances reported by oil palm estates through the e-submissions portal spanning from January 2015 to June 2023, we focused on elucidating the impacts of ENSO on fresh fruit bunch yield, oil extraction rate, and oil yield. We found that both El Niño and La Niña conditions, characterized by extreme levels of ENSO indices cumulated over lags of 0–23 months prior to harvest, were associated with statistically significant reductions in yields. Lag association patterns unveiled that production risks were linked to pre-harvest exposure to extreme ENSO indices in various time windows. Subgroup analyses further revealed that the effects were pronounced in labor-intensive estates and those lacking fertilizer investments. This study underscores the necessity for adaptation strategies in response to future climate anomalies.

## Highlights

• There was little evidence on the role of climate anomalies in oil palm productivity at the estate level.
• This is the first detailed report of how El Niño Southern Oscillation (ENSO) affects the yields of oil palm in 2015–2023.
• Associations of ENSO measures with fresh fruit bunch and oil yields were non-linear with nearly inverted-U shapes.
• Extreme ENSO levels were associated with reduced yields in various pre-harvest time windows.
• Labor intensity, fertilizer investment, and tree age can confer the susceptibility of ENSO exposure.

## 1 Introduction

As one of the most productive oil crops, demonstrating exceptional yield per hectare [1], oil palm contributes positively to the world across various domains. First, it plays a pivotal role in the economies of producing countries like Malaysia and Indonesia, acting as a major export commodity and contributing positively to employment and income [2,3]. Second, its primary derivative, palm oil, serves as a versatile global trade commodity, finding application in a diverse array of products ranging from food items and cosmetics to biofuels [[4], [5], [6]]. Moreover, scientists have innovatively repurposed its byproducts, such as the empty fruit bunch, into valuable resources or raw materials [7]. Despite these significant benefits, the cultivation of oil palm plantations raises valid concerns surrounding environmental and social challenges, including reduced biodiversity, deforestation, environmental pollution, depletion of peatlands, land rights conflicts, and fair labor practices [2,[8], [9], [10]]. Given the potential trade-offs between economic benefits and environmental impacts of oil palm plantations, it becomes imperative to comprehensively understand how the yield of oil palm is determined and to decide what is the optimal balance as climate and weather conditions evolve. Enhancing the productivity of oil palm can mitigate the need for additional plantations [11], preserving valuable land resources, while suboptimal productivity in oil palm cultivation can pose challenges for promoting sustainable practices within the industry.

One important determinant of oil palm productivity is climate variability, with El Niño-Southern Oscillation (ENSO) serving as a major source of such variability. ENSO, a periodic and irregular climatic phenomenon characterized by variations in sea surface temperature, pressure, and wind in the equatorial Pacific Ocean occurring every three to seven years, plays a pivotal role in shaping environmental conditions [12]. Research into ENSO has raised concerns about the large-scale detrimental effects of climate variability on oil palm yield [[13], [14], [15], [16], [17]]. The interannual variability of rainfall in Malaysia is highly affected by ENSO [18,19], and studies have suggested that yields in the year following an El Niño event tend to be less than that on average [13]. Besides below-normal rainfall, El Niño events are also associated with warmer temperatures, increasing the likelihood of heatwaves. The risk of wildfires rises as well in hot and extremely dry conditions. Moreover, given the negative impacts of extreme ENSO levels on human health [20], labor productivity is likely decreased not only during El Niño events but also La Niña events, which eventually reduces yields. Thus, we hypothesized that extreme ENSO levels can lower key indicators of oil palm productivity.

There were discussions on the linear impacts of ENSO on oil palm yield, but these studies often concentrated solely on the correlations between the two extreme phases of ENSO (El Niño and La Niña events) and yield, overlooking essential economic determinants in the production process, utilizing less frequent (e.g., annual) data, and employing ad hoc lag specification without considering the combined effects of multiple lags and their interactions. In this research, we seek to enhance the understanding of these aspects by investigating the cumulative and nonlinear lagged impacts of ENSO, using the latest monthly data while carefully controlling for economic factors. While nonlinear impacts of ENSO indices have been explored in the literature, much of the focus has been on their impacts on macroeconomic factors or human health [21,22]. Therefore, this study serves as an extension, specifically examining their impacts on oil palm yield.

A critical inquiry arises concerning whether changes in ENSO cycles exacerbate the risks of palm oil production. This paper addresses this question by assessing ENSO's impacts on the fresh fruit bunch (FFB) and palm oil yields in Malaysia, the second-largest producer of palm oil in the world. Using historical climate and palm oil production data, we find that, there are substantial negative impacts from both La Niña (cool phase) and El Niño (warm phase) on oil palm production. Notably, the effects of La Niña are found to be larger than pre-research expectations. Furthermore, the critical exposure windows for La Niña and El Niño differ, with the former being longer and less recent. The study also examines the effect modification of indicators relevant to locations, labor intensity, fertilizer inputs, age profiles of trees, soil quality, as well as environmental conditions that may confer susceptibility to climate-related effects.

## 2 Methods

### 2.1 Study Population and design

We compiled a multi-state estate-based retrospective dataset of *oil palm trees* based on the administrative records of Malaysian Palm Oil Board (MPOB), with *cross-sectional observations* reported monthly from January 2015 to June 2023. Note that, while data were repeatedly drawn from the same oil palm plantations, they typically reflected different subareas of the plantations and thus different trees. In terms of holdings, 73.6 % of the planted areas were owned by private and government agency estates, 14.5 % owned by independent smallholders (with a size of 40.45 ha [ha] or below), and 11.9 % owned by organized smallholders. Since independent smallholders were not liable to report their performances regularly to MPOB, we did not include them in our analyses, leading to 85.5 % of the oil palm areas in Malaysia covered by our sample in 2023.

We excluded extreme values from our analyses—52 observations were with an average yield of FFB greater than 6 metric tons per hectare per month (t/ha/month) or an average oil extraction rate (OER) of over 30 % and were thus not included, resulting in a final sample of 424,185 observations after exclusion. Based on Supplemental Table S3, we coded missing values of each variable as an independent category. For example, soil quality score is only available for 231,481 observations, and the remaining 192,704 observations are marked by missing soil quality information. As fertilizer cost is reported annually, we did not have the fertilizer investment information for 28,571 observations in 2023; we also did not record fertilizer cost in 2015, leading to missingness for another 41,218 observations. We did obtain the information for the remaining 83.5 % of the sample, among which 32.3 % did not invest in any fertilizers in the corresponding calendar year. We treated observations with missing or no investment in fertilizers as our reference group in the analysis. For all other variables defined in Supplemental Table S2, data are available for all observations.

### 2.2 Outcome measurement

Data on the size of harvested area and the amount of FFB were reported by estates to MPOB monthly, which were used to calculate the FFB yield (see Supplemental Table S2 for definition). The average OER measured the amount of palm oil obtained from all the harvested FFB in proportion for each estate—it is a weighted average of the mill-level OER across the mills that handled the corresponding estate's FFB. However, as mills typically handled FFB from multiple estates, this is not a perfect measure, and hence the conclusions for OER should be read with cautions. Finally, the oil yield was simply the FFB yield multiplied by OER, calculated at the estate level.

### 2.3 Covariates

Information on covariates that may confound the impacts of ENSO on yields were collected from multiple sources. One important type of confounders was prices, which could affect the supply and demand of palm oil, leading to behavioral changes of oil palm farmers and thus changes in yields. Therefore, we obtained prices for five types of fertilizers— phosphate rock, diammonium phosphate, triple superphosphate, prilled urea, and muriate of potash. These are the major fertilizers reported in the World Bank Commodity Price Data (The Pink Sheet). We also considered the average price of crude palm oil. Given that these prices were nominal (not adjusted for inflations) and palm oil was mainly exported from Malaysia, we also controlled for the average commodity price index (CPI) and nominal exchange rate. The sources were provided in Supplemental Table S2. Other economic variables included labor intensity—the average number of harvesters per hectare of harvested area, and fertilizer investment—the annual fertilizer and application cost, including fertilizer purchases, labor costs to apply fertilizer, and machinery use and maintenance for fertilizing activities.

We further considered soil quality, which was measured in 2021. The proportion of each type of soil (“alluvial”, “inland”, “shallow peat”, “laterite”, “acid sulphate”, “deep peat” and “sandy” soils) was measured, and then a score between 1 and 7 was calculated for each estate, which was the weighted average of the score of each type (i.e., “alluvial” was the best and had a score of 7, while “sandy” was the worst with a score of 1). Then, we derived the average age profile of oil palm trees from the areas reported according year of planting (weighted average) based on MPOB e-submissions. Finally, given that our research time frame spans through the pandemic, we collected the monthly number of confirmed COVID-19 cases and constructed an indicator for any lockdown policies in the corresponding state of each estate.

After getting data ready for all the above variables, we used a Least Absolute Shrinkage and Selection Operator (LASSO) regression method to refine the above-mentioned covariates for each outcome separately, to avoid overfitting. This approach has been widely applied to account for the potential multicollinearity between covariates and to facilitate variable selections [23]. Covariates with three or more categories were firstly transformed into multiple dummy variables before LASSO regressions. A generalized linear model was fitted through penalized maximum likelihood, and the minimum mean cross-validated error parameter lambda (λ) was generated from cross-validation. Then, λ was re-introduced to compute the optimal coefficients of each covariate. The fitting plot of λ and screening variables for outcome measures were presented in Supplemental Figs. S4–S6. Apart from covariates determined by LASSO, all our regressions additionally adjusted for the month and state fixed-effects to explain differences across states and months that may be unobserved.

### 2.4 ENSO and weather data

ENSO is a large, complex, and dynamic system, involving different aspects of the ocean and the atmosphere over the tropical Pacific. Using multiple different indexes derived from various measurements at sea level, such as sea surface temperature and pressure, instead of a single index, can be informative and beneficial in measuring the overall ENSO state. We collected monthly levels of various ENSO indices, including the multivariate El Niño index (MEI), Niño 1 + 2 abnormal index, Niño 3.4 abnormal index, oceanic Niño index (ONI), Southern Oscillation Index (SOI), and the Bivariate ENSO Timeseries (BEST) index from the United States National Oceanic and Atmospheric Administration (NOAA). Supplemental Table S1 provided the detailed definitions and data sources of these measures. In general, lower values of ENSO indices indicate more La Niña conditions, while higher values represent more El Niño conditions [24]. We hypothesized that ENSO-driven climate anomalies could adversely affect oil palm yields through its impacts on weather conditions and economic activities, and thus collected monthly meteorologic parameters through the Copernicus Climate Data Store. We obtained European Centre for Medium-Range Weather Forecasts (ECMWF)'s ERA5-Land monthly averaged data from 1950 to present, and then matched the address of each estate to the air and soil temperature and total precipitation in the nearest 9 km by 9 km grid. We calculated the 24-month average in the current month and the past 23 months. Details are described in Supplemental Table S2.

### 2.5 Statistical analysis

**Fig. 1:**
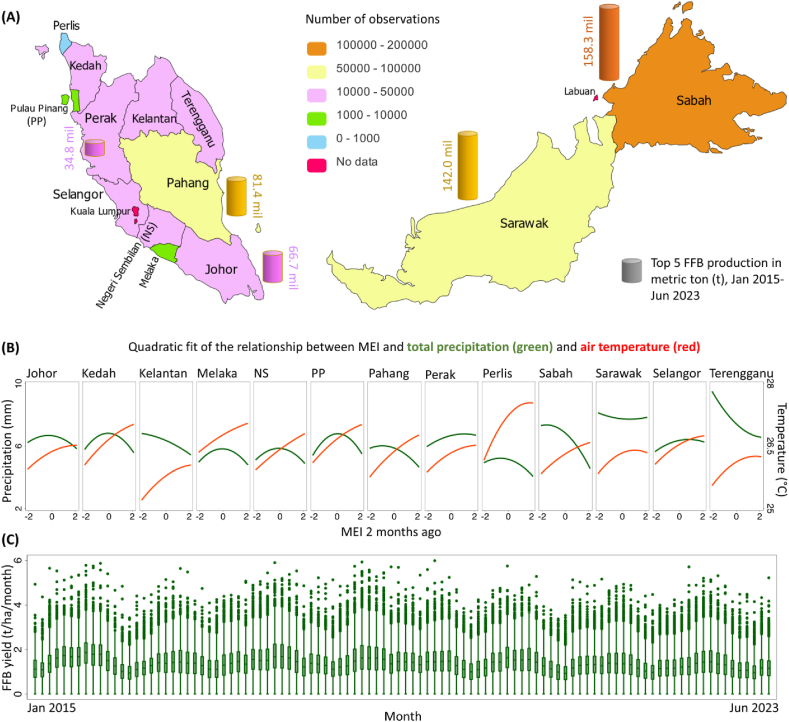
Distribution of the study areas with ENSO tele-connected impacts. (A) is the geographic locations of 13 states with data on FFB yield included in this analysis, with the top 5 FFB production in millions (mil.) of metric ton (t) during January 2015 to June 2023 depicted on the map; (B) shows the monthly correlations between the two-month lag of MEI and meteorologic parameters (total precipitation and air temperature respectively) in within each estate's nearest 9 km by 9 km grid based on quadratic fits; (C) provides the FFB yield (t/ha/month) distribution over time in tele-connected states (n = 424,185 observations from 4785 estates). Box plots indicate median (middle line), 25th, 75th percentile (box), and 5th and 95th percentile (whiskers) as well as outliers (single points). Estate composition within each month is different due to entries or exits of estates. Abbreviations: ENSO, El Niño Southern Oscillation; MEI, multivariate El Niño index; FFB, fresh fruit bunch.

We calculated descriptive statistics (mean, standard deviation [SD], minimum [min] and maximum [max]) to summarize characteristics of study estates and outcomes. We also ran ordinary least square (OLS) regressions of local levels of air temperature and total precipitation on MEI and its squared term to generate quadratic fits for each state. These results were shown in Supplemental Table S3 and Fig. 1(B) respectively.

The associations between pre-harvest exposure to ENSO and oil palm yields were then evaluated by generalized additive models (GAMs). One key reason for this choice is that, although our monthly data could be treated as a pseudo-panel dataset of estates from an economic perspective, they typically reflected different harvested areas (and oil palm trees) every month from a biological perspective. Moreover, GAMs have several merits that suit our research purposes well. For example, they can help to fit non-linear relationships automatically without attempting different transformations manually [25]. Given the growing evidence on the potential non-linear impacts of ENSO, we incorporated GAMs with distributed lag non-linear models (DLNM), providing us a flexible framework to characterize delayed associations [22,26,27]. A key advantage of DLNM is that it is originally developed for time series data, and it allows for a better separation of correlated exposure periods. In line with previous studies, our models included the cross-basis function of monthly ENSO measures built by the DLNM, which were specified by B-spline using the “bs” function. Regarding the space of lags in the cross-basis function, three knots were placed at equally-spaced log scales using the “lognots” function [22,28,29]. Given that previous research emphasized the adverse effects of lagged exposures to climate anomalies on crop yields, we focused on examining the overall cumulative associations with a variety of ENSO indices over the last two years prior to the harvest. The maximum number of lags is 23 months for FFB and oil yields, and 10 months for OER, based on the smallest Akaike information criterion (AIC) values in the GAMs with DLNM for each outcome. Further, the lag association patterns depicted by contour plots were performed to graphically discern potentially sensitive windows of exposure [30].

Subgroup analyses were conducted to examine the effect modification of geographic and production factors, including location (west versus east), labor intensity (bottom quartile or low, second quartile or medium-low, third quartile or medium-high, and top 25 % or high), fertilizer cost (0 or missing, low, medium-low, medium-high, and high), average age profile (0–3 years or immature, 4–8 years or young, 9–18 years or prime, 19–23 years or ageing, and ≥24 years or old), soil quality in 2021 (missing, bottom 25 % or worst, second quartile or fair, third quartile or good, and top quartile or best). Also, we assessed whether the associations with ENSO exposure might differ in estates exposed to different levels of weather conditions. Thus, we further conducted subgroup analyses across tertiles of average air temperature, soil temperature and precipitation during the lagged period. The differential association estimates between two subgroups were tested using 2-sample z-tests, and the formula was as follows:

$$\left(\beta_{1}-\beta_{2}\right)\pm 1.96\sqrt{{\left({SE}_{1}\right)}^{2}+{\left({SE}_{2}\right)}^{2}}$$

where $\beta_{1}$ and $\beta_{2}$ are the association estimates for the two subgroups, ${SE}_{1}$ and ${SE}_{2}$ are their respective standard errors; the statistical significance for comparisons was determined at *P* < 0.05 for two subgroups, 0.025 for three subgroups, and 0.0125 for five groups [31].

The results for ENSO exposure derived from our GAMs coupled with DLNM were presented as the differences with 95 % CIs by calculating the impact of a given ENSO value relative to the reference value (zero, close to the medium value during the study period) to explore non-linear relationships with ENSO exposure [26]. Based on the interval of exposure-response curves, we calculated the effect of moderate La Niña conditions one year prior to birth on child mortality by comparing the exposure level of −1.0 with the reference value (zero), and the effect of strong El Niño conditions was calculated by comparing the exposure level of +2.0 with the reference value (zero).

Given the nested structure of our data in which observations were repeatedly drawn from the same oil palm plantations, we conducted a robustness check that controlled for the estate random effects. We fit generalized linear mixed models (GLMMs) through penalized quasi-likelihood (PQL) combined with DLNM.

All data analyses were conducted between October and June 2024 and analyzed using R, version 4.3.2 (R Project for Statistical Computing), and the following packages were primarily used: package “mgcv” was installed to characterize associations with outcome measures; package “glmnet” was loaded to select variables by LASSO; package “dlnm” was applied to specify the cross-basis function for ENSO exposure and to predict and graphically plot the results of the fitted regression models.

## 3 Summary of region and ENSO exposure in the study period

As shown in Fig. 1(A), all 13 states of Malaysia (11 from the West or Peninsular Malaysia, and two from the East or Borneo Malaysia) were included in our analysis. Monthly observations from January 2015 to June 2023 are collected from 4785 oil palm estates. Notably, the top three productive states—Sabah, Sarawak, and Pahang—produced more than 380 million metric tons (t) of FFB in total during this period. Supplemental Table S3 provides a summary of the dataset, a total of 424,185 observations are recorded, among which the average FFB yield is 1.36 t/ha/month. With an average OER of 18.71 %, the oil yield is calculated to be 0.26 t/ha/month. Of the observations, 213,592 (50.35 %) are from estates in which oil palm trees were on average in their prime years (aged 9–18 years); 239,894 (56.55 %) are from estates that invested in fertilizers in the corresponding calendar year, and among them, the average cost was 1135.28 thousand Malaysia Ringgit. Estates labor intensity is reflected in a maximum of 5.41 workers/ha to harvest FFB, while the average is 4 workers per 100 ha.

Fig. S1 displays substantial variations in ENSO measures, including MEI and ONI, over the past decade. There were nine ENSO events observed during our period of study. Among the four El Niño events, two were weak (MEI 0.5 to 0.9), one was strong (MEI 1.5 to 1.9), and one was very strong (MEI 2.0 and above); among the five La Niña events, three were weak (MEI −0.9 to −0.5), two were moderate (MEI −1.4 to −1.0). Note that, there were no moderate El Niño events (MEI 1.0 to 1.4) or strong La Niña events (MEI −1.5 and below) during our study period. Overall, month-to-month change patterns across all ENSO measures, with high values observed during 2015–2016 and the mid-2023 (the most recent time point), suggesting the impacts of El Niño events. The quadratic fits by state in Fig. 1(B) showed that MEI was nonlinearly related to regional precipitation and temperature conditions in different ways, consistent with existing research [32,33]. For instance, in the east coast of the Peninsular Malaysia (Johor and Pahang), moderate La Niña (−1.4 to −1.0) rather than strong La Niña (MEI lower than −1.5) caused a significant increase in wet precipitation extremes. Based on Supplemental Table S3, the average temperature in each month ranges from 18.65 to 30.40 °C, leading to an average of 26.24 °C across our observations; the average precipitation ranges from 0.06 to 33.01 mm/day, leading to an average of 7.09 mm/day (or 212.7 mm per month). Fig. 1(B) further highlights regional differences, such as more observations with lower air temperature in Kelantan and higher precipitation in Sarawak and Terengganu.

Importantly Fig. 1(C) emphasizes that, the production of FFB is expanded throughout the year in Malaysia [34], and it is featured by seasonality due to variations in reproductive growth [1]. Typically, minimum yields occur in February, while peaks are observed in September and October. To account for these variations, we thus control for the month and state fixed-effects in all models.

### 3.1 Regression results

**Fig. 2:**
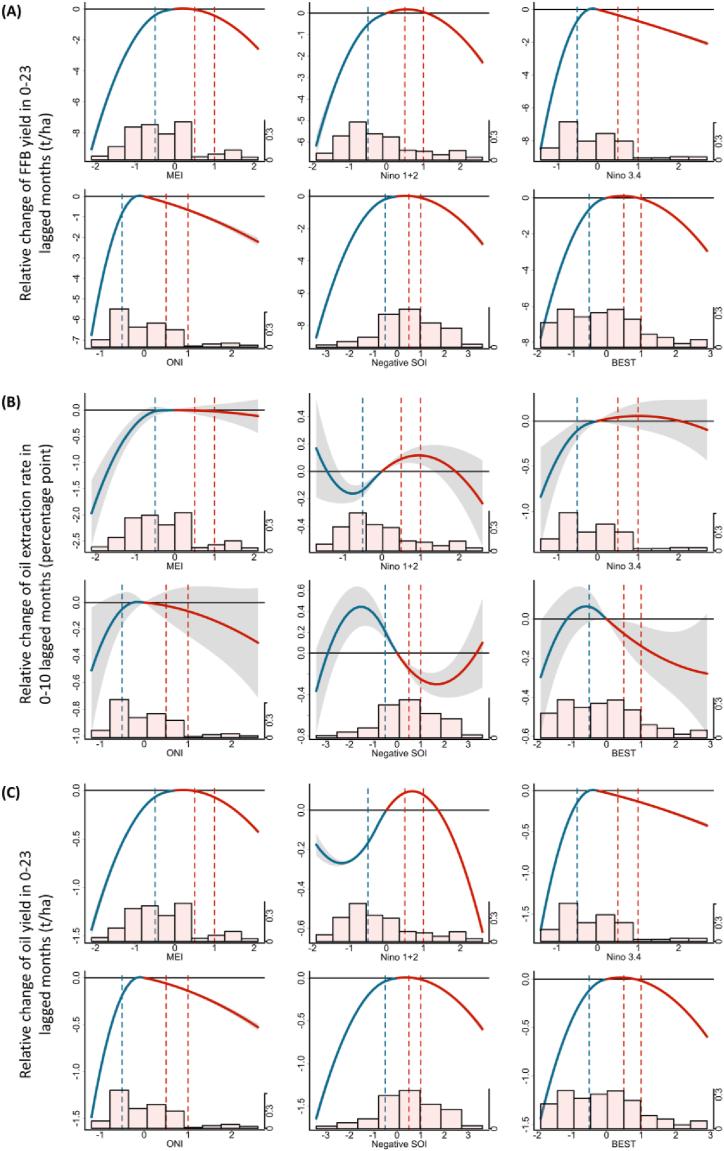
Cumulative exposure-response associations between ENSO and the relative changes of FFB yield (in 0–23), average OER (in 0–11), and oil yield (in 0–23 lagged months). The red lines (with 95 % confidence intervals [CIs], shaded grey) indicate effect estimates of El Niño conditions, and blue lines (with 95 % CIs, shaded grey) indicate effect estimates from La Niña conditions. The association of each ENSO measure with each outcome is computed as the effect of a given value of ENSO measure relative to the reference value (set at zero) Histograms of ENSO indices are plotted at the bottom, with proportions measured by the second (right) vertical axis. Abbreviations: t/ha, metric ton per hectare; ENSO, El Niño Southern Oscillation; MEI, multivariate El Niño index; ONI, Oceanic Niño Index; SOI, Southern Oscillation Index; BEST, Bivariate ENSO Timeseries. Refer to Fig. S2 for the results controlling for estate random effects.

Fig. 2 presents the overall cumulative associations between the relative changes of our outcomes of interest (i.e., FFB yield, OER, and oil yield) and their lagged ENSO exposure during the two years preceding harvest. The lags included in the models were determined based on the AIC, as detailed in the Methods section. In general, these curves exhibit an approximately inverted U-shaped relationship between ENSO and yields, with extreme ENSO levels (La Niña and El Niño conditions) associated with greater production risks than the normal levels (i.e., when indices are zero). The relationship between ENSO and extraction rate is less distinct, with Niño 1 + 2 and (negative) SOI showing different patterns. According to Table 1, comparing a high level of MEI exposure (2.0) to the reference value (zero) at lag 0–23 months, the cumulative differences in FFB and oil yields were −2.30 (95 % confidence interval [CI], −2.41, −2.18) t/ha and −0.38 (95 % CI, −0.40, −0.36) t/ha for 24 months, which can be translated into −0.10 and −0.02 t/ha/month, or 7 % and 6 % of their means. The difference between the FFB yield at high levels (2.0) of other ENSO indices (i.e., Niño 1 + 2, Niño 3.4, ONI, negative SOI, and the BEST index) and that at the reference value (zero) ranged from −0.73 (95 % CI, −0.80, −0.66) t/ha to −1.56 (95 % CI, −1.69, −1.42) t/ha. Moreover, the associations between ENSO and the relative changes of FFB yield were usually stronger at the low levels ENSO indices (−1.0), with estimates ranging from −0.67 (95 % CI, −0.72, −0.62) to −4.48 (95 % CI, −4.73, −4.22). For oil yield, similar patterns were found—relative to zero, the effect estimates ranged from −0.15 (95 % CI, −0.16, −0.13) to −0.36 (95 % CI, −0.38, −0.33) at high values (2.0) and from −0.13 (95 % CI, −0.15, −0.12) to −0.99 (95 % CI, −1.04, −0.94) at low levels (−1.0), translating into 2–16 % of the mean value.

**Table 1:** Cumulative associations between the relative changes of FFB yield, average OER, and oil yield and extreme levels of ENSO exposure relative to zero.

ENSO indicator	Indicator value	Outcome, lagged months		
		FFB Yield (t/ha), 0-23	Average OER (%), 0-10	Oil Yield (t/ha), 0-23
MEI	−1	−1.83 (−1.91, −1.74)	−0.28 (−0.44, −0.12)	−0.30 (−0.31, −0.28)
	2	−2.30 (−2.41, −2.18)	−0.10 (−0.40, 0.20)	−0.38 (−0.40, −0.36)
Niño 1 + 2	−1	−1.80 (−1.84, −1.75)	−0.14 (−0.23, −0.05)	−0.26 (−0.27, −0.25)
	2	−1.06 (−1.12, −0.99)	−0.03 (−0.21, 0.16)	−0.24 (−0.25, −0.23)
Niño 3.4	−1	−3.97 (−4.17, −3.77)	−0.42 (−0.69, −0.16)	−0.80 (−0.84, −0.76)
	2	−1.48 (−1.58, −1.39)	0.00 (−0.23, 0.24)	−0.29 (−0.31, −0.27)
ONI	−1	−4.48 (−4.73, −4.22)	−0.33 (−0.66, 0.00)	−0.99 (−1.04, −0.94)
	2	−1.56 (−1.69, −1.42)	−0.19 (−0.50, 0.11)	−0.36 (−0.38, −0.33)
Negative SOI	−1	−0.67 (−0.72, −0.62)	0.38 (0.24, 0.52)	−0.13 (−0.15, −0.12)
	2	−0.73 (−0.80, −0.66)	−0.29 (−0.43, −0.15)	−0.15 (−0.16, −0.13)
BEST	−1	−2.26 (−2.36, −2.16)	0.03 (−0.10, 0.16)	−0.42 (−0.44, −0.40)
	2	−1.11 (−1.17, −1.04)	−0.23 (−0.40, −0.07)	−0.23 (−0.24, −0.22)

Abbreviations: t/ha, metric ton per hectare; ENSO, El Niño Southern Oscillation; FFB, fresh fruit bunch; OER, oil exchange rate; MEI, multivariate El Niño index; ONI, oceanic Niño index; SOI, Southern Oscillation Index; BEST, Bivariate ENSO Timeseries.

**Fig. 3:**
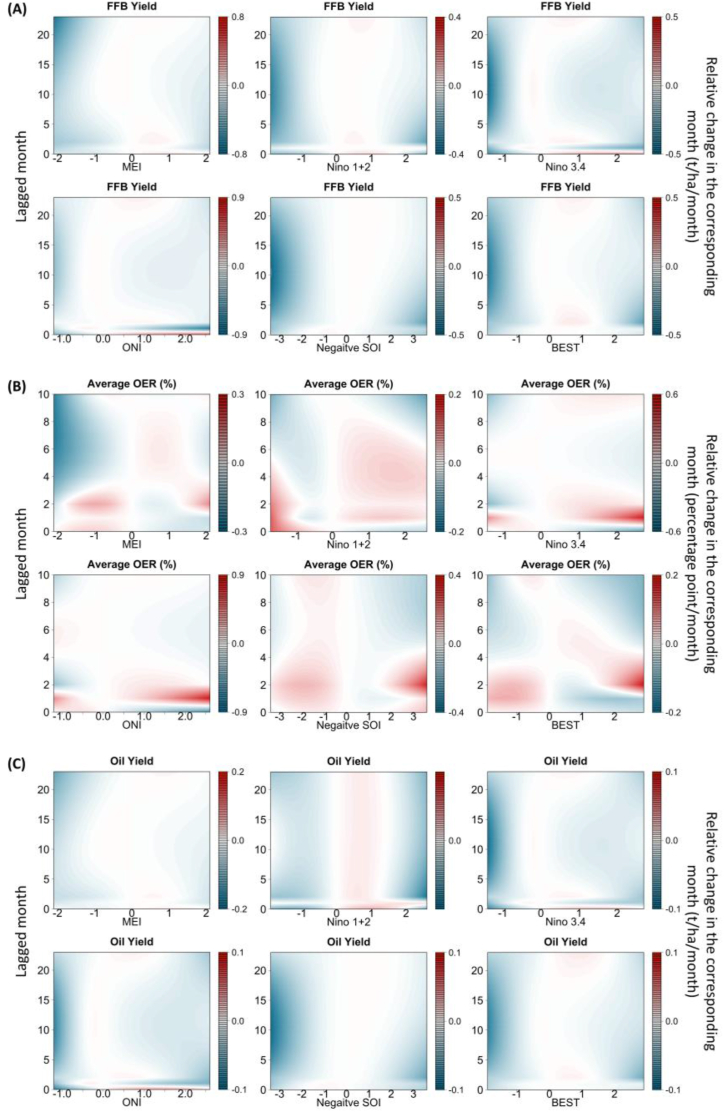
Lag association pattern between ENSO (up to 23 months ago) and the FFB yield, average OER, and oil yield from contour plots. The X-axis indicates intensity of each ENSO measure, and Y-axis indicates lags of 0–23 months. The color gradient represents the relative change. Abbreviations: t/ha/month, metric ton per hectare per month; ENSO, El Niño Southern Oscillation; MEI, multivariate El Niño index; ONI, Oceanic Niño Index; SOI, Southern Oscillation Index; BEST, Bivariate ENSO Timeseries. Refer to Fig. S3 for the results controlling for estate random effects.

Fig. 3 graphically depicts the lag association pattern of ENSO exposure at individual lagged months prior to the harvest using contour plots. Overall, we found that increased risks of yield reduction were associated with extreme levels of ENSO measures in most lagged months; however, the highest risks for FFB yield were usually found in the 1st-6th lagged months for extremely high levels of ENSO measures, and in the 0^th^-20th and 22nd-23rd lagged months for extremely low levels of ENSO measures. Table 2 provides the results for low (but not extremely low) and extremely high levels of MEI. It turns out that the highest risks for FFB yield were found in the 0^th^-3rd and 22nd-23rd rather than the 4th-20th lagged months when MEI is equal to −1.0, which suggests weaker risks during moderate La Niña compared to strong La Niña in the 4th-20th lagged months, potentially due to relatively more precipitation in Peninsular Malaysia. In terms of oil yield, similar patterns were found, but for low levels of MEI (−1.0), the negative impacts were not found in the 7th-14th lagged months. Regarding OER, risks of extraction rate reduction were typically found in the 6th-10th lagged months for extremely low levels of ENSO exposures, while elevations in extraction rate could be observed in the 1st-4th lagged months for extremely high levels of ENSO exposures.

**Table 2:** Lag association pattern between the relative changes of FFB yield, average OER, and oil yield and extreme levels of ENSO exposure relative to zero.

Lag	At the value of −1			At the value of 2		
	FFB yield (t/ha/month)	Average OER (%/month)	Oil yield (t/ha/month)	FFB yield (t/ha/month)	Average OER (%/month)	Oil yield (t/ha/month)
0	−0.11 (−0.13, −0.10)	0.06 (−0.04, 0.15)	−0.02 (−0.02, −0.01)	0.05 (0.01, 0.09)	−0.05 (−0.26, 0.17)	0.01 (0.00, 0.02)
1	−0.07 (−0.09, −0.06)	0.01 (−0.10, 0.12)	−0.01 (−0.01, −0.01)	−0.21 (−0.25, −0.17)	−0.06 (−0.31, 0.19)	−0.03 (−0.04, −0.02)
2	−0.17 (−0.18, −0.17)	0.10 (0.04, 0.16)	−0.03 (−0.03, −0.03)	−0.17 (−0.18, −0.16)	0.12 (−0.04, 0.28)	−0.03 (−0.03, −0.03)
3	−0.14 (−0.14, −0.13)	0.01 (−0.02, 0.04)	−0.02 (−0.02, −0.02)	−0.16 (−0.17, −0.15)	0.04 (−0.02, 0.11)	−0.03 (−0.03, −0.03)
4	−0.11 (−0.11, −0.10)	−0.05 (−0.07, −0.03)	−0.02 (−0.02, −0.02)	−0.15 (−0.16, −0.14)	−0.01 (−0.04, 0.03)	−0.03 (−0.03, −0.03)
5	−0.08 (−0.09, −0.08)	−0.09 (−0.11, −0.07)	−0.01 (−0.01, −0.01)	−0.14 (−0.15, −0.14)	−0.04 (−0.08, 0.01)	−0.03 (−0.03, −0.02)
6	−0.06 (−0.07, −0.06)	−0.10 (−0.12, −0.08)	−0.01 (−0.01, −0.01)	−0.14 (−0.14, −0.13)	−0.05 (−0.10, 0.00)	−0.02 (−0.03, −0.02)
7	−0.05 (−0.05, −0.04)	−0.09 (−0.11, −0.07)	−0.01 (−0.01, 0.00)	−0.13 (−0.13, −0.12)	−0.04 (−0.08, 0.00)	−0.02 (−0.02, −0.02)
8	−0.04 (−0.04, −0.03)	−0.07 (−0.09, −0.05)	0.00 (0.00, 0.00)	−0.12 (−0.13, −0.12)	−0.03 (−0.07, 0.01)	−0.02 (−0.02, −0.02)
9	−0.03 (−0.03, −0.02)	−0.04 (−0.07, −0.02)	0.00 (0.00, 0.00)	−0.12 (−0.12, −0.11)	−0.01 (−0.07, 0.05)	−0.02 (−0.02, −0.02)
10	−0.02 (−0.03, −0.02)	−0.01 (−0.05, 0.03)	0.00 (0.00, 0.00)	−0.11 (−0.11, −0.10)	0.02 (−0.08, 0.12)	−0.02 (−0.02, −0.02)
11	−0.02 (−0.03, −0.01)	–	0.00 (0.00, 0.00)	−0.10 (−0.11, −0.10)	–	−0.02 (−0.02, −0.02)
12	−0.02 (−0.03, −0.02)	–	0.00 (0.00, 0.00)	−0.10 (−0.10, −0.09)	–	−0.02 (−0.02, −0.02)
13	−0.03 (−0.03, −0.02)	–	0.00 (0.00, 0.00)	−0.09 (−0.10, −0.09)	–	−0.01 (−0.02, −0.01)
14	−0.03 (−0.04, −0.03)	–	0.00 (−0.01, 0.00)	−0.08 (−0.09, −0.08)	–	−0.01 (−0.01, −0.01)
15	−0.04 (−0.04, −0.03)	–	−0.01 (−0.01, −0.01)	−0.08 (−0.08, −0.07)	–	−0.01 (−0.01, −0.01)
16	−0.05 (−0.06, −0.04)	–	−0.01 (−0.01, −0.01)	−0.07 (−0.08, −0.07)	–	−0.01 (−0.01, −0.01)
17	−0.06 (−0.07, −0.06)	–	−0.01 (−0.01, −0.01)	−0.07 (−0.07, −0.06)	–	−0.01 (−0.01, −0.01)
18	−0.08 (−0.08, −0.07)	–	−0.01 (−0.02, −0.01)	−0.06 (−0.07, −0.06)	–	−0.01 (−0.01, −0.01)
19	−0.09 (−0.09, −0.09)	–	−0.02 (−0.02, −0.02)	−0.06 (−0.06, −0.05)	–	−0.01 (−0.01, −0.01)
20	−0.11 (−0.11, −0.10)	–	−0.02 (−0.02, −0.02)	−0.05 (−0.06, −0.05)	–	−0.01 (−0.01, −0.01)
21	−0.12 (−0.13, −0.12)	–	−0.02 (−0.02, −0.02)	−0.05 (−0.05, −0.04)	–	−0.01 (−0.01, −0.01)
22	−0.14 (−0.14, −0.13)	–	−0.03 (−0.03, −0.03)	−0.04 (−0.05, −0.04)	–	−0.01 (−0.01, 0.00)
23	−0.16 (−0.16, −0.15)	–	−0.03 (−0.03, −0.03)	−0.04 (−0.04, −0.03)	–	0.00 (−0.01, 0.00)

Abbreviations: t/ha/month, metric ton per hectare per month; FFB, fresh fruit bunch; OER, oil exchange rate; MEI, multivariate El Niño index.

As a sensitivity check, we take into account the random effects of 4785 unique plantations and generate the cumulative associations and lag association pattern, as shown by Fig. S2 and Fig. S3 in the supplementary materials. Given the fact that data are from different trees of the plantations and we have controlled for as many plantation-level covariates as possible, the results do not vary much after the inclusion of estate random effects. For example, comparing a high level of MEI exposure (2.0) to the reference value (zero) at lag 0–23 months, the cumulative differences in FFB and oil yields were −2.85 (95 % CI, −2.92, −2.78) t/ha and −0.48 (95 % CI, −0.49, −0.46) t/ha for 24 months, which can be translated into −0.12 and −0.02 t/ha/month, or 9 % and 8 % of their means. When random effects are more specifically accounted for, results typically become larger in magnitude and are likely more significant. Thus, our primary results may serve as lower bounds or “conservative” estimates.

### 3.2 Exploratory analyses

**Fig. 4:**
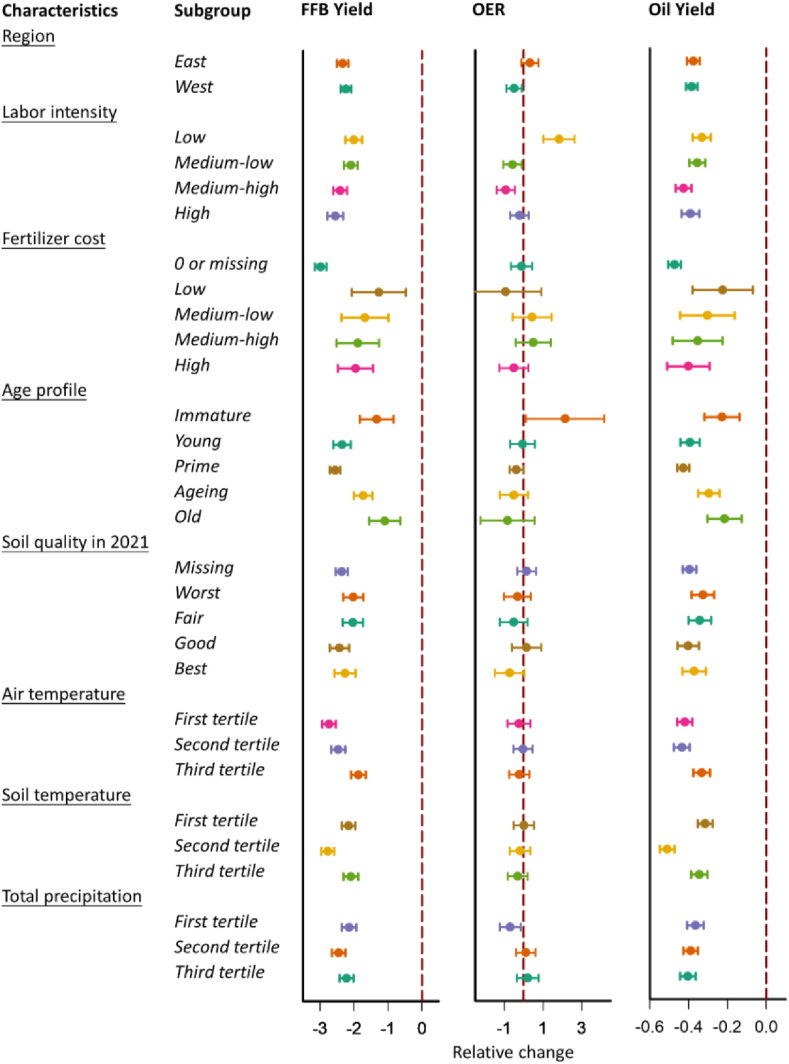
Cumulative associations between the relative changes of FFB yield (t/ha in 0–23), average OER (% in 0–11), and oil yield (t/ha in 0–23 lagged months) and MEI exposure at the value of 2 stratified by characteristics of study estates. The association of MEI with each outcome is computed as the effect of a given value of MEI relative to the reference (i.e., zero). Abbreviations: t/ha, metric ton per hectare; MEI, multivariate El Niño index; FFB, fresh fruit bunch; OER, oil extraction rate.

**Fig. 5:**
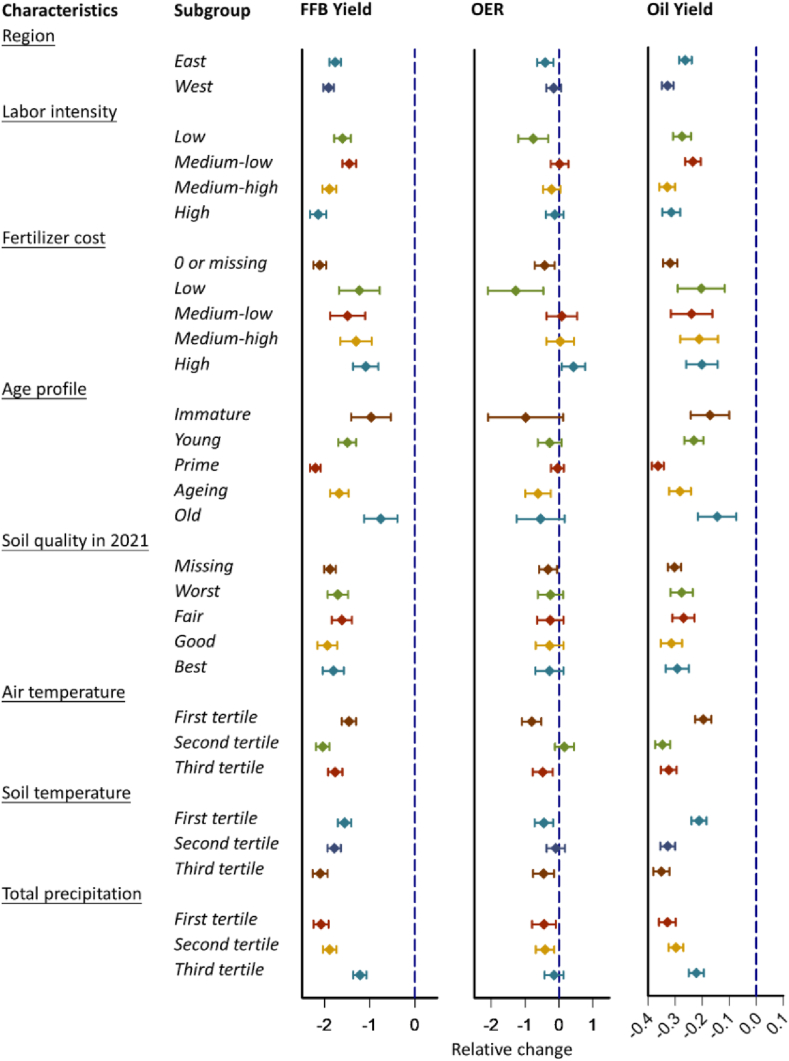
Cumulative associations between the relative changes of FFB yield (t/ha in 0–23), average OER (% in 0–11), and oil yield (t/ha in 0–23 lagged months) and MEI exposure at the value of −1 stratified by characteristics of study estates. The association of MEI with each outcome is computed as the effect of a given value of MEI relative to the reference (i.e., zero). Abbreviations: t/ha, metric ton per hectare; MEI, multivariate El Niño index; FFB, fresh fruit bunch; OER, oil extraction rate.

Fig. 4, Fig. 5, Tables S4–S9, and Figs. S7–S16 show the associations of our outcomes with ENSO exposure during the months prior to the harvest by location, labor intensity, fertilizer cost, age profile of trees, soil quality, air temperature, soil temperature, and total precipitation. Across these covariates, we found some heterogeneity in the associations between extreme levels of ENSO measures and yield reduction risks across all the covariates listed above, except for location and soil quality. Compared to the reference value (zero), the difference in FFB yield for MEI at the value of 2.0 was −2.01 (95 % CI, −2.25, −1.76) in estates that required only the lowest labor intensity, but the effect in those requiring the highest labor intensity was higher (effect, −2.55; 95 % CI, −2.79, −2.32). This pattern persisted for MEI at the value of −1.0 and the oil yield, albeit with medium-high labor intensity estates exhibiting higher risks of oil yield reduction. Interestingly, fertilizers appeared to play an important role in mitigating yield reduction risks imposed by extreme levels of ENSO measures. For example, the difference in FFB yield for MEI at the value of 2.0 was −2.98 (95 % CI, −3.16, −2.81) in estates that did not invest in fertilizers in the harvest year or lost records on fertilizer investment, but the effect was lower in those required the highest labor intensity, with effects ranging from −1.27 (95 % CI, −2.06, −0.47) to −1.96 (95 % CI, −2.48, −1.44). We also found that immature and old oil palm trees were less sensitive to climate anomalies, whereas prime trees tended to be more affected by both El Niño and La Niña conditions. For example, the differences in oil yield for MEI at the value of −1.0 were −0.17 (95 % CI, −0.24, −0.10) in estates with predominantly immature trees and −0.14 (95 % CI, −0.22, −0.07) in those with mainly old trees, but the effect in those with trees in prime years on average was higher (effect, −0.36; 95 % CI, −0.39, −0.34). Finally, estates in hot regions (with average air or soil temperature in the second and third tertiles) tended to have their FFB and oil yields less (more) sensitive to El Niño (La Niña) conditions, compared to those in cool regions (with average temperature in the first tertile). Similarly, estates in humid areas (with average total precipitation in the third tertile) tended to have their FFB and oil yields less sensitive to La Niña conditions.

As for OER, noteworthy patterns emerged. First, the estates in Peninsular Malaysia experienced more significant and negative impacts on OER from El Niño conditions than those in Borneo Malaysia (Sabah and Sarawak). Second, the estates that did not require much labor intensity tended to have OER more significantly and positively boosted by El Niño conditions but more negatively impacted by La Niña conditions, compared to the more labor-intensive estates. Third, estates with limited fertilizer investment tended to have their OER more negatively by La Niña conditions. Fourth, the estates with predominantly immature trees tended to have their OER boosted by El Niño conditions, while other estates did not seem to receive any positive effects. Finally, the estates in dry areas (with average precipitation in the first tertile) were more susceptible to El Niño conditions.

## 4 Discussion

In this study, we used an up-to-date monthly administrative dataset to systematically explore the relationship between climate anomalies and oil palm productivity. We have shown here that exposure to ENSO over the past two years prior to the harvest was significantly associated with higher risks of yield reduction. Unlike previous studies suggesting that La Niña may be favorable for oil palm production in Malaysia [13,14], we discovered that, after considering the nonlinearity in impacts, interactions between monthly lags, and economic confounders, La Niña could impose larger negative impacts on yields. Subgroup analyses further unveiled that various estate characteristics and weather conditions modified the linkages between ENSO-driven climate anomalies and oil palm productivity in Malaysia. Specifically, the most labor-intensive estates, the ones without fertilizer investment in the current year, and the ones with trees in their prime years on average are more susceptible to the negative impacts of extreme ENSO levels on yields. Additionally, estates in hotter regions demonstrated less sensitivity to El Niño conditions but more sensitivity to La Niña conditions compared to those in cooler regions. Furthermore, estates in drier areas displayed greater sensitivity to La Niña conditions compared to those in the most humid areas.

Several findings from our study contribute nuanced insights to existing scientific knowledge. First, rainfall plays an important role in the impacts of climate anomalies on oil palm yields. Fig. 1(B) shows that, particularly during La Niña conditions, rainfall in some states could be higher than usual—this additional rainfall may lead to excessiveness, causing a reduction in FFB yields [35]. While the amount of monthly rainfall has been found to significantly impact oil palm FFB yield mainly through inflorescence, abortion, and pollination after a 5-month lag period [36]. This extends the critical exposure window to the 6th-23rd lagged months as well, likely through flooding and reduced oil palm cultivation [37]. Moreover, estates in humid areas may have better adapted to the high rainfall environment and thus were less vulnerable to excessive rainfall. Nevertheless, our results highlight the importance of dealing with rainfall risks, especially during La Niña phases, given that rainfall variability of ±32 % is estimated to cause a drop in annual palm oil earnings of about 1181 US dollars [[37], [38], [39]].

Second, temperature is also a key mechanism underlying our findings. As depicted in Fig. 1(B), temperature typically rises with MEI in all states, while rainfall hits its lows under El Niño conditions. As found by existing research, when temperature rises by 1–4 °C, oil palm production is expected to decline by 10–40 % [40]. Combining high temperature with water stress could lead to a decrease in male and female flowers, which could affect the formation or development of FFB and cause a drop in crude palm oil production [1,41]. However, this channel is more long-term rather than immediate and direct [17], while our results also underline the key exposure window in the 1st-6th lagged months. It is quite likely that the current practices in Malaysia have adapted well to its hot environment, especially in areas with higher average air temperatures indicated by our subgroup analyses. Therefore, there could still be concerns about the increased rate of abortion, rotten fruit bunch, elongated inflorescences of 8–9 months, expanded lance leaf number and frond break, and the ecology of pest and disease-causing organisms, pollinators, and weeds [[42], [43], [44]].

Third, our findings emphasize the significance of fertilizer investment as an essential adaptation strategy to climate change. As suggested by the literature, agroecological practices, e.g., sustainable use of recommended fertilizer, can help to improve soil fertility and boost FFB yield [45]. Undoubtedly, managing the chemical fertility of soils through fertilizer application is the aspect that farmers have paid the most attention to Ref. [46]. Our results show that, even minimal investment in fertilizers could make a huge difference, compared to no investment at all; while more investment may not be necessary, in terms of improving resilience under climate change (Supplemental Table S5). Reduced use of chemicals and fertilizers could be achieved through intercropping or silt pit [44], which may have been adopted by some oil palm farmers in Malaysia. These accompanying adaptation strategies together can strengthen the sustainability and resilience of oil palm estates in both El Niño and La Niña phases.

Recent research has found that the frequency of extreme El Niño and La Niña events has increased, and the extreme ENSO events may further increase in frequency from about one every two decades to one every decade by the end of the 21st century under greenhouse warming [47,48]. This suggests that ENSO is likely to be a bigger concern for oil palm productivity in the coming decades under aggressive greenhouse gas emission scenarios, because yield reductions can occur more frequently under the influences of more frequent extreme ENSO levels.

Our study possesses several strengths. To our knowledge, the environmental determinants of oil palm productivity from the perspective of ENSO-driven climate variability remains to be fully characterized. Herein, we utilized a recent administrative dataset of oil palm estates in Malaysia in 2015–2023. Using the DLNM allowed us to characterize the impacts of pre-harvest exposure to ENSO on yields in more details. Nevertheless, certain limitations in the current study should be noted. First, despite controlling for a wealth of covariates, e.g., fluctuations in prices and nominal exchange rates, residual confounding due to unmeasured covariates in this observational study cannot be entirely ruled out. For example, our study period spans through the COVID-19 pandemic (2020–2022), which impacted economic activities greatly; although we have controlled for both the number of confirmed cases and an indicator for state-level lockdown policies, we may not perfectly control for all the shocks from the pandemic. Second, the relatively short study period of nine years may limit the generalization of findings to encompass major ENSO events from previous decades. Although Fig. S1 shows substantial variations in our ENSO measures, the number of El Niño events (two weak events, 2014/15 and 2018/19, with a 0.5–0.9 MEI; one strong event, 2023/24, with a 1.5–1.9 MEI; one very strong event, 2015/16 with MEI of 2.0 or above) and La Niña events (three weak events, 2016/17, 2017/18, and 2022/23, with MEI of −0.9 to −0.5; two moderate events, 2020/21 and 2021/22, with MEI of −1.4 to −1.0) observed during this recent decade may not be enough for researchers to investigate the general impacts of the major events (e.g., there were neither moderate El Niño events nor strong La Niña events during our period of study). Ideally, we would expand the research time frame to include the strong El Niño events of 1951/52, 1953/54, 1957/58, 1965/66, 1969/70, 1972/73, 1982/83, 1986/87, 1991/92, 1994/95, 1997/98, 2000/01, 2003/05, 2007/08 and La Niña events of 1975/76, 1988/89, 1998/99, and 2000/01 [32,44,49]. However, critical economic variables would be missing in those years. Hence, we advocate for continuous attention to this topic and the use of new data in the future to investigate again the nonlinear relationship between ENSO and oil palm yield with a more extended timeframe.

## 5 Conclusion

In summary, this research unveils the nonlinear exposure-response relationships and association patterns between pre-harvest exposure to ENSO and oil palm yields in Malaysia. A significant implication arises, underscoring the pivotal role of extreme levels of climate variability driven by ENSO as a crucial environmental risk factor for oil palm production in the subsequent two years. The magnitudes of such effects were higher for estates with high labor intensity, zero fertilizer investment, and trees in their prime years on average. These findings highlight the ongoing imperative for the development and implementation of more effective adaptation strategies to prevent and mitigate the adverse effects of ENSO-related climate anomalies on oil palm production.

## Funding

10.13039/501100012226Fundamental Research Funds for the Central Universities, Peking University 7100604271 (CCZ), Peking University School of Economics Research Seed Grant (CCZ), 10.13039/501100012456National Social Science Fund of China 22VMG017 (CCZ), 10.13039/100014717National Natural Science Foundation of China 71973008 (CCZ)

## Data and materials availability

The authors do not have the permission to distribute the estate-level data from MPOB. The data on the monthly intensity of all the ENSO measures used in this study can be downloaded at https://psl.noaa.gov/data/climateindices. The data on prices, CPI, nominal exchange rate, COVID-19 cases, lockdown status, and weather conditions are publicly available, and their sources are provided in Table S2. Replication codes can be found at https://github.com/castielzhuang/MPOB1.

## CRediT authorship contribution statement

**Nur Nadia Kamil:** Writing – review & editing, Writing – original draft, Supervision, Resources, Project administration. **Saizi Xiao:** Writing – review & editing, Writing – original draft, Supervision, Resources, Project administration, Investigation, Data curation, Conceptualization. **Sharifah Nabilah Syed Salleh:** Writing – review & editing, Supervision, Project administration. **Hongbing Xu:** Writing – review & editing, Visualization, Validation, Software, Methodology, Investigation, Formal analysis, Data curation. **Castiel Chen Zhuang:** Writing – review & editing, Writing – original draft, Visualization, Validation, Methodology, Investigation, Funding acquisition, Formal analysis, Data curation, Conceptualization.

## Declaration of competing interest

The authors declare that they have no known competing financial interests or personal relationships that could have appeared to influence the work reported in this paper.
