# Supplementary material for: Nonlinear impacts of climate anomalies on oil palm productivity
Source: Heliyon. 2024 Aug 6;10(15):e35798. doi: 10.1016/j.heliyon.2024.e35798 (PMC11337023; doi:10.1016/j.heliyon.2024.e35798)
Supplement: Multimedia component 1 [file mmc1.docx]

Supplementary Materials for

**Nonlinear Impacts of Climate Anomalies on Oil Palm Productivity**

Nur Nadia Kamil, Saizi Xiao, Sharifah Nabilah Syed Salleh, Hongbing Xu, Castiel Chen Zhuang*

Corresponding author: zogcee@gmail.com

**The PDF file includes:**

Figs. S1 to S16

Tables S1 to S9

**
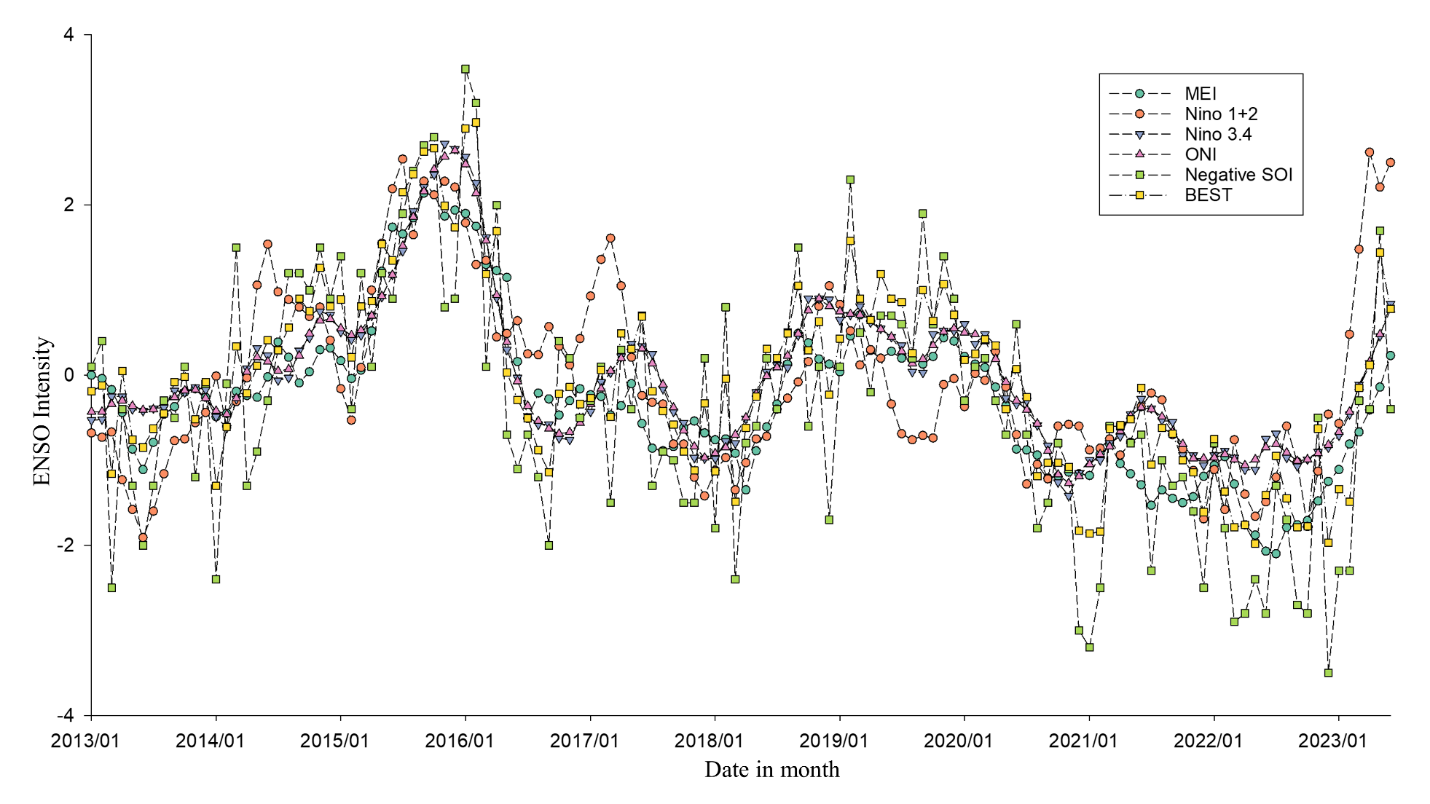
**

Fig. S1.

**The time-series pattern of ENSO indices throughout the study period.** Abbreviations: ENSO, El Niño Southern Oscillation; MEI, multivariate El Niño index; ONI, oceanic Niño index; SOI, Southern Oscillation Index; BEST, Bivariate ENSO Timeseries.

**
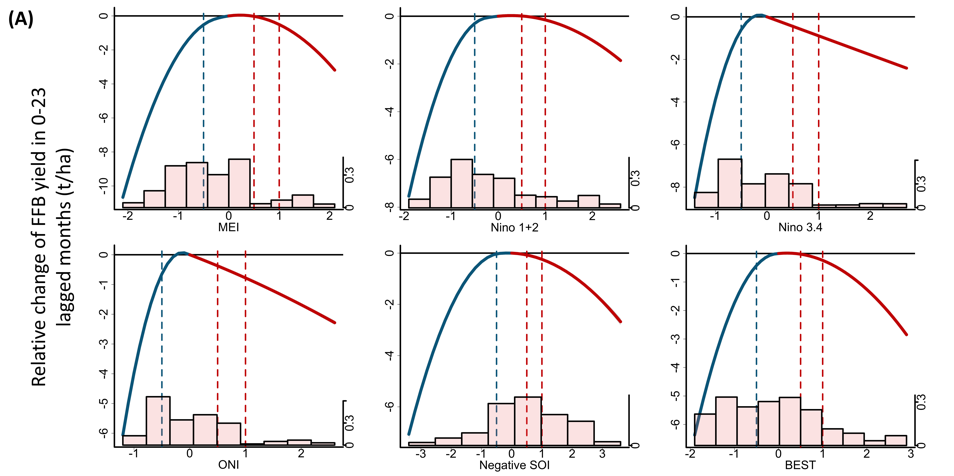

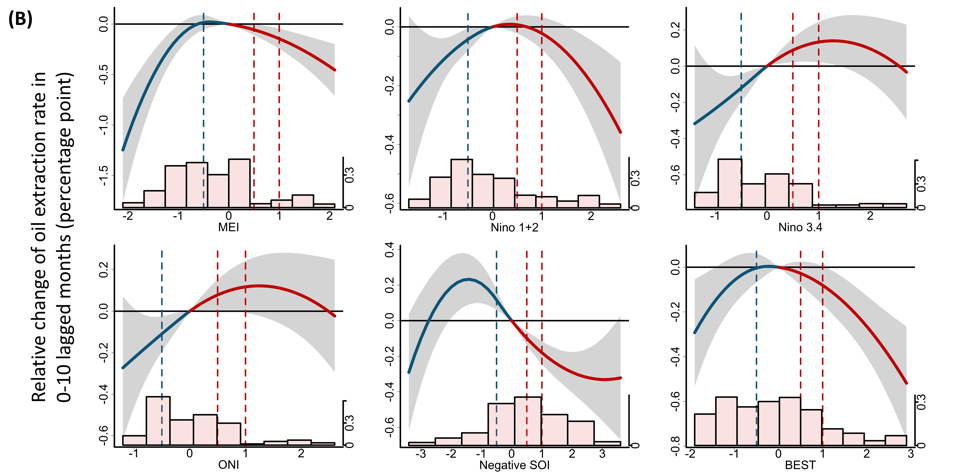

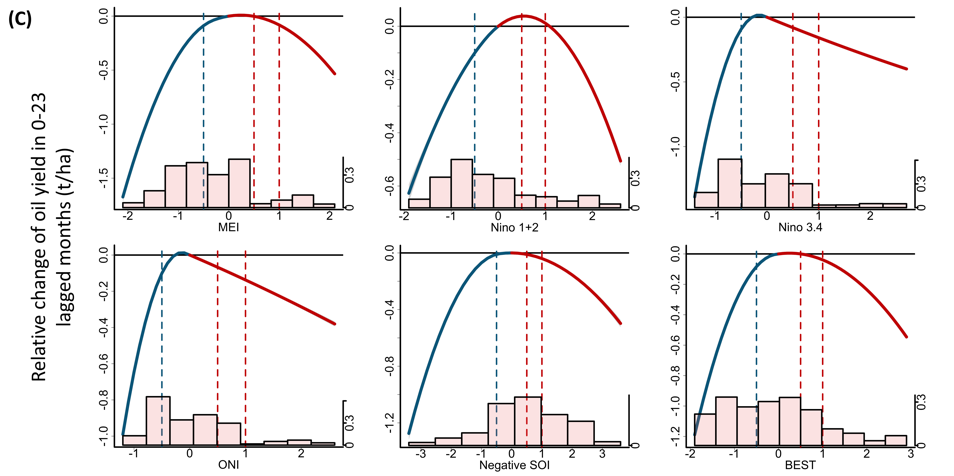
**

**Fig. S2. Cumulative exposure-response associations between ENSO and the relative changes of FFB yield (in 0-23), average OER (in 0-11), and oil yield (in 0-23 lagged months), controlling for estate random effects.** The red lines (with 95% confidence intervals [CIs], shaded grey) indicate effect estimates of El Niño conditions, and blue lines (with 95% CIs, shaded grey) indicate effect estimates from La Niña conditions. The association of each ENSO measure with each outcome is computed as the effect of a given value of ENSO measure relative to the reference value (set at zero) Histograms of ENSO indices are plotted at the bottom, with proportios measured by the second (right) vertical axis. Abbreviations: t/ha, metric ton per hectare; ENSO, El Niño Southern Oscillation; MEI, multivariate El Niño index; ONI, Oceanic Niño Index; SOI, Southern Oscillation Index; BEST, Bivariate ENSO Timeseries.

**
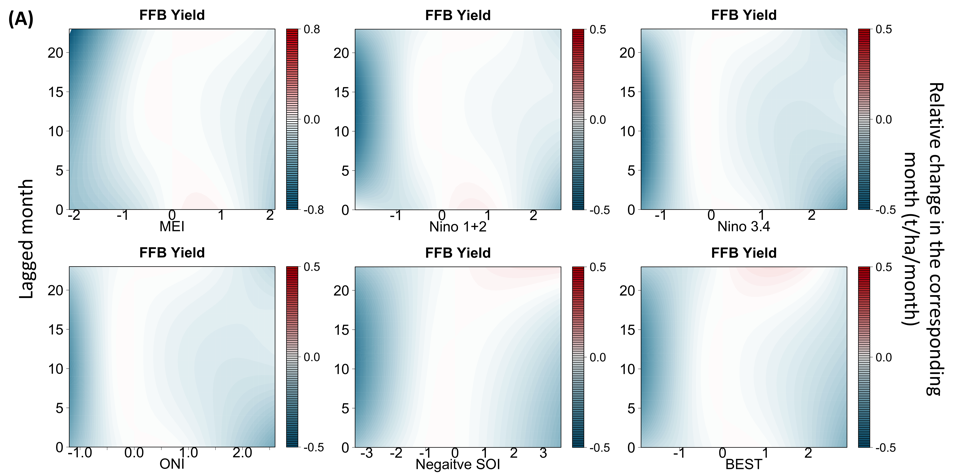

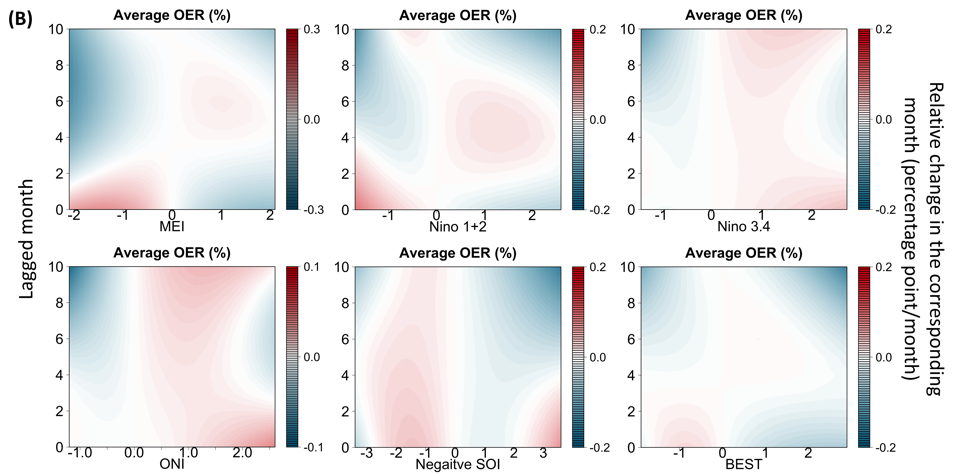

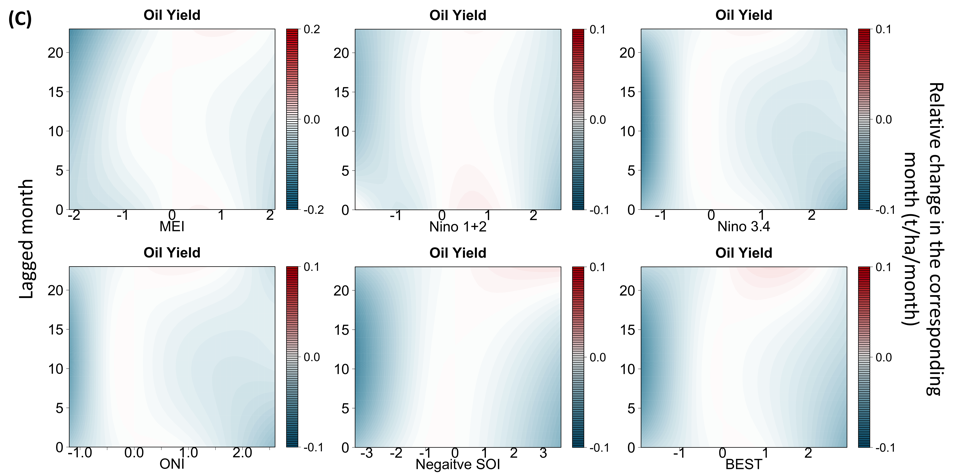
**

**Fig. 3. Lag association pattern between ENSO (up to 23 months ago) and the FFB yield, average OER, and oil yield from contour plots, controlling for estate random effects.** The X-axis indicates intensity of each ENSO measure, and Y-axis indicates lags of 0-23 months. The color gradient represents the relative change. Abbreviations: t/ha/month, metric ton per hectare and month; ENSO, El Niño Southern Oscillation; MEI, multivariate El Niño index; ONI, Oceanic Niño Index; SOI, Southern Oscillation Index; BEST, Bivariate ENSO Timeseries.


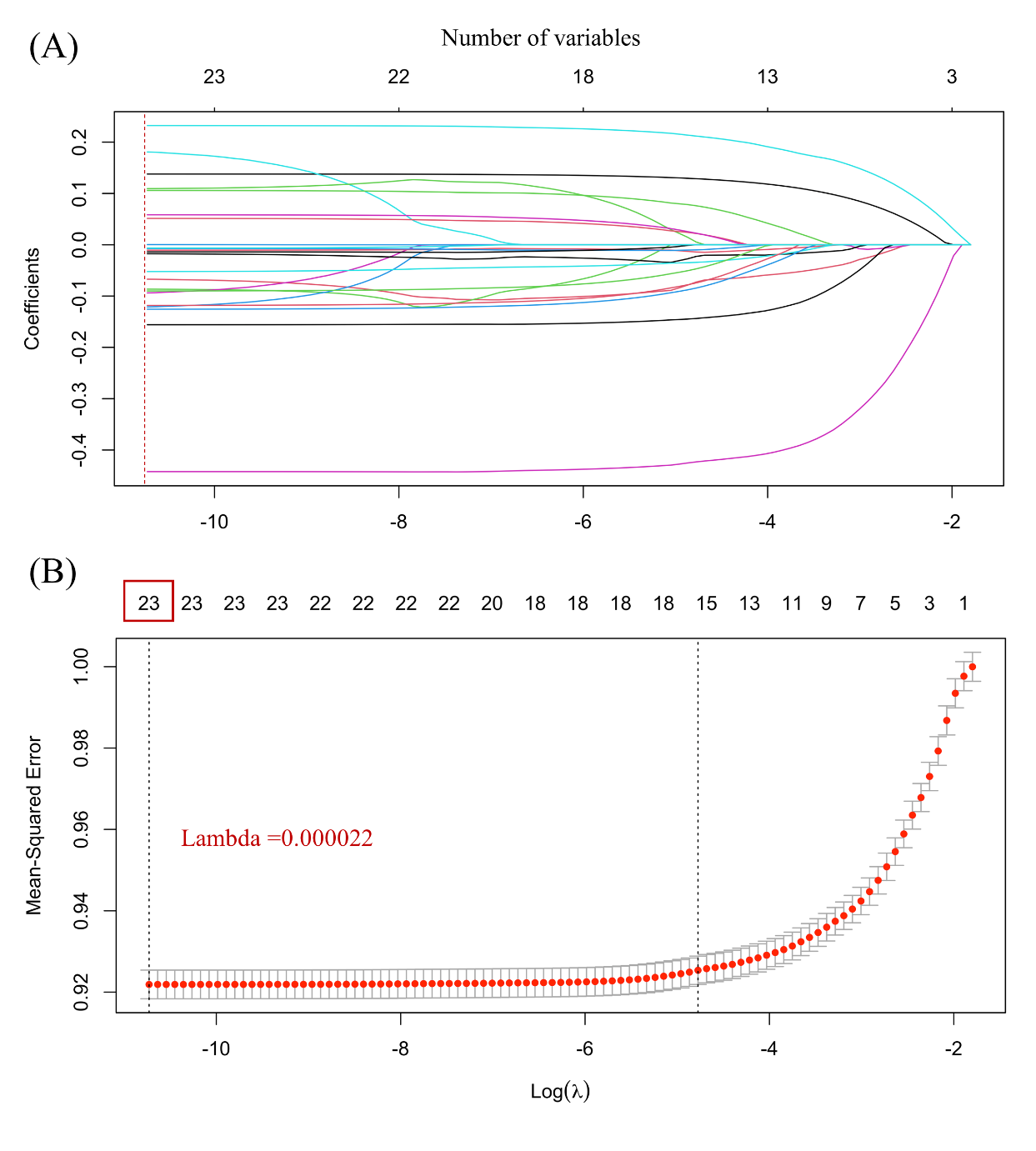


Fig. S4.

**Selection of covariates for the fresh fruit bunch yield by LASSO—the fitting plot of λ: (A) The fitting process of λ; (B) the plot of cross-validation curve.** The 23 non-zero coefficients correspond to all the control variables described in table S2 and summarized in table S3.


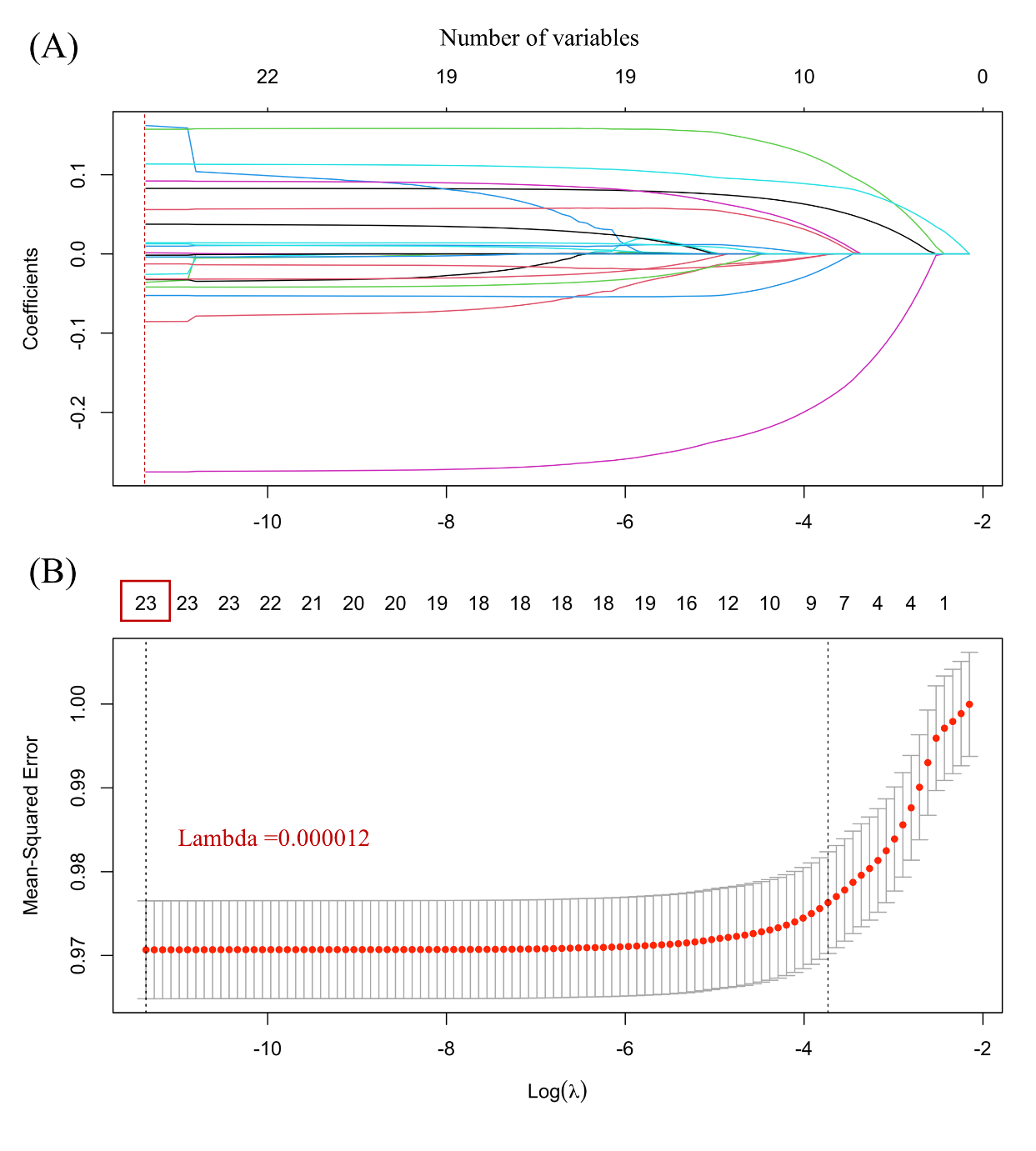


Fig. S5.

**Selection of covariates for oil extraction rate by LASSO—the fitting plot of λ: (A) The fitting process of λ; (B) the plot of cross-validation curve.** The 23 non-zero coefficients correspond to all the control variables described in table S2 and summarized in table S3.


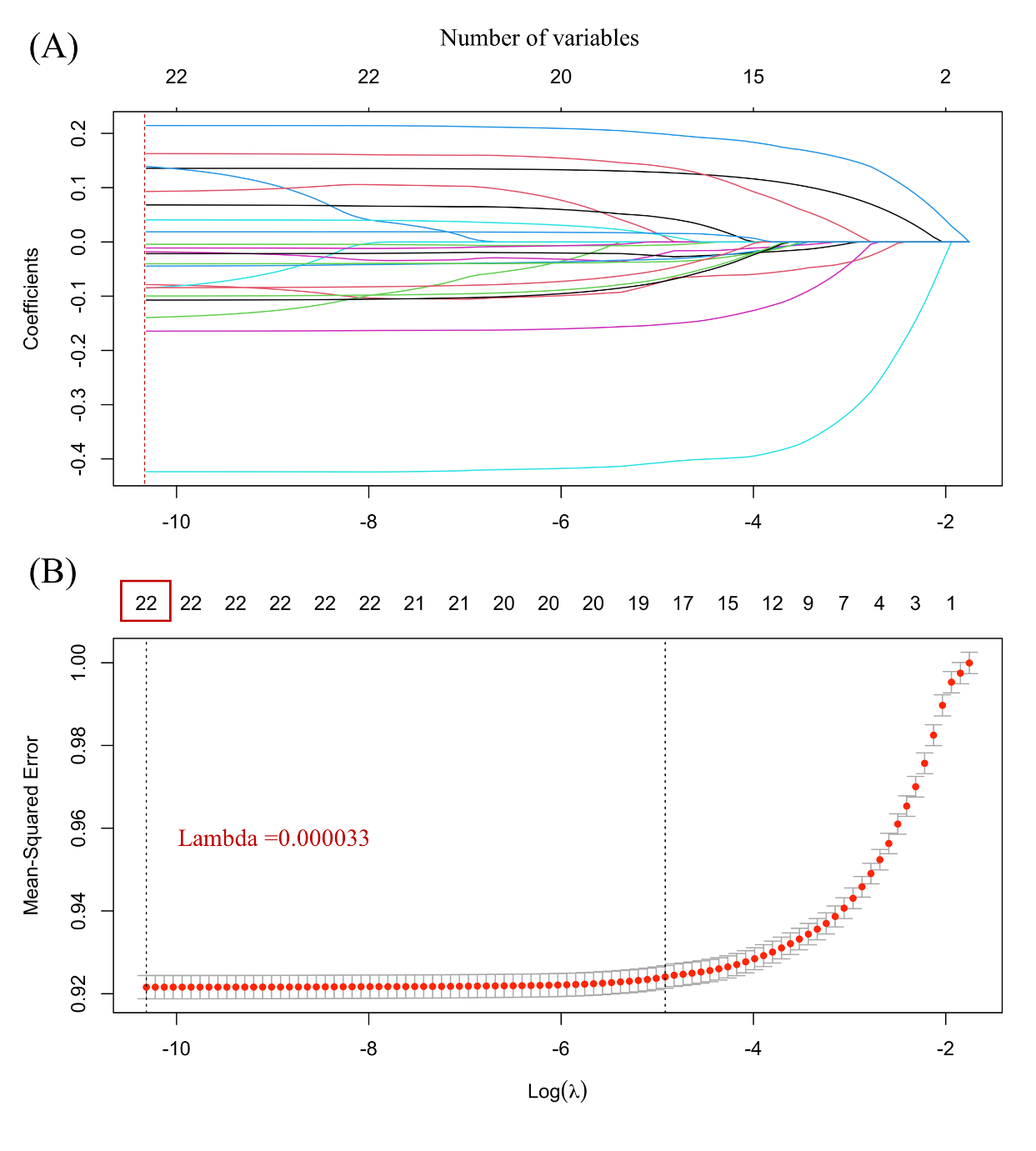


Fig. S6.

**Selection of covariates for the oil yield by LASSO—the fitting plot of λ: (A) The fitting process of λ; (B) the plot of cross-validation curve.** The 22 non-zero coefficients correspond to all the control variables described in table S2 and summarized in table S3, except for the price of triple superphosphate (TSP), which is excluded by LASSO.


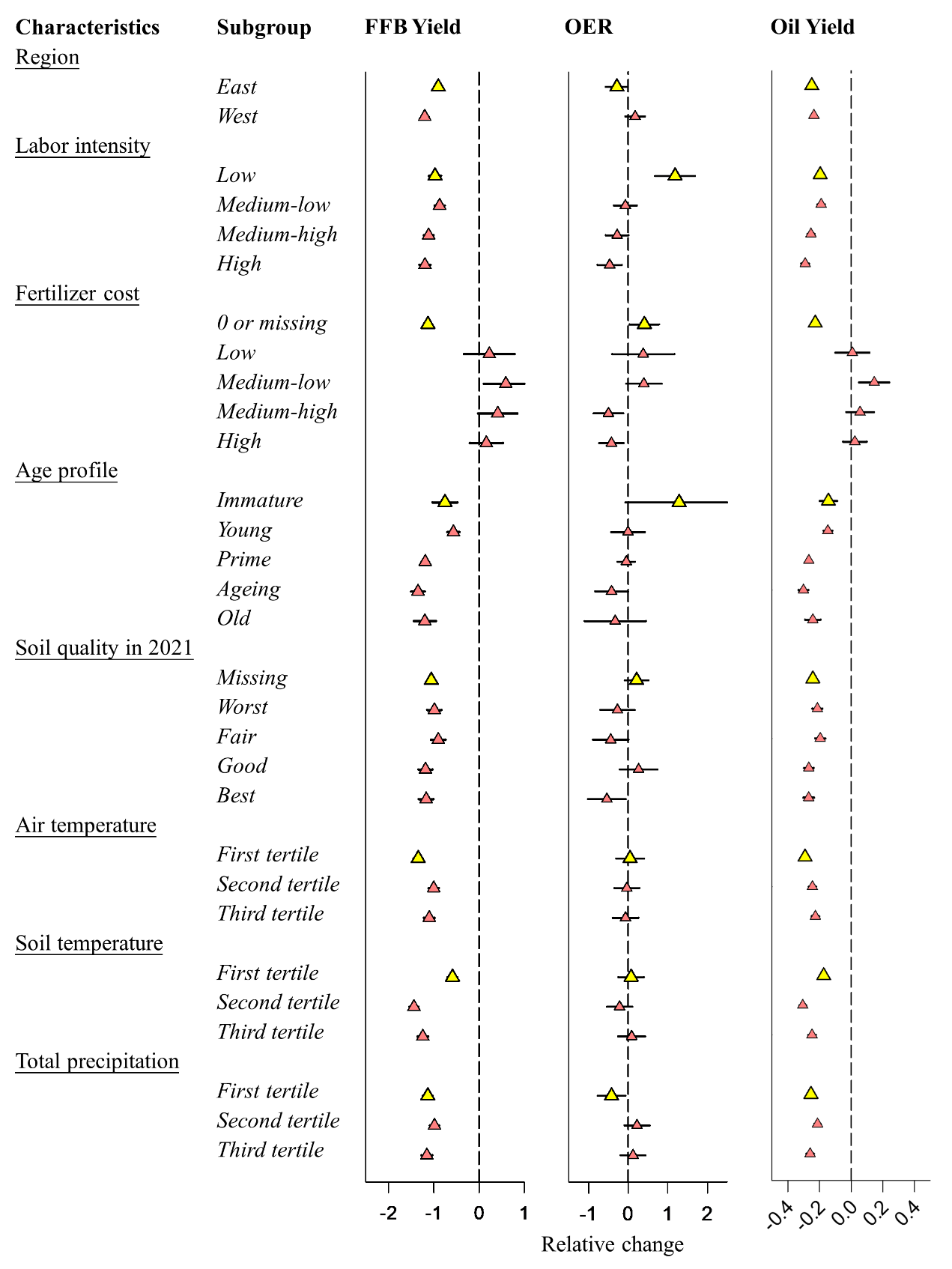


Fig. S7.

**Cumulative associations between the FFB yield (t/ha, in 0-23 lagged months), average OER (%, in 0-10 lagged months), and oil yield (t/ha, in 0-23 lagged months) and Niño 1+2 exposure at the value of 2 stratified by characteristics of study estates.** Notes: (1) Abbreviations: FFB, fresh fruit bunch; OER, oil extraction rate. (2) For the definitions and data sources of each variable, refer to Extended Data Table S2. (3) Reference groups are the first subgroup of each characteristic with a different color.


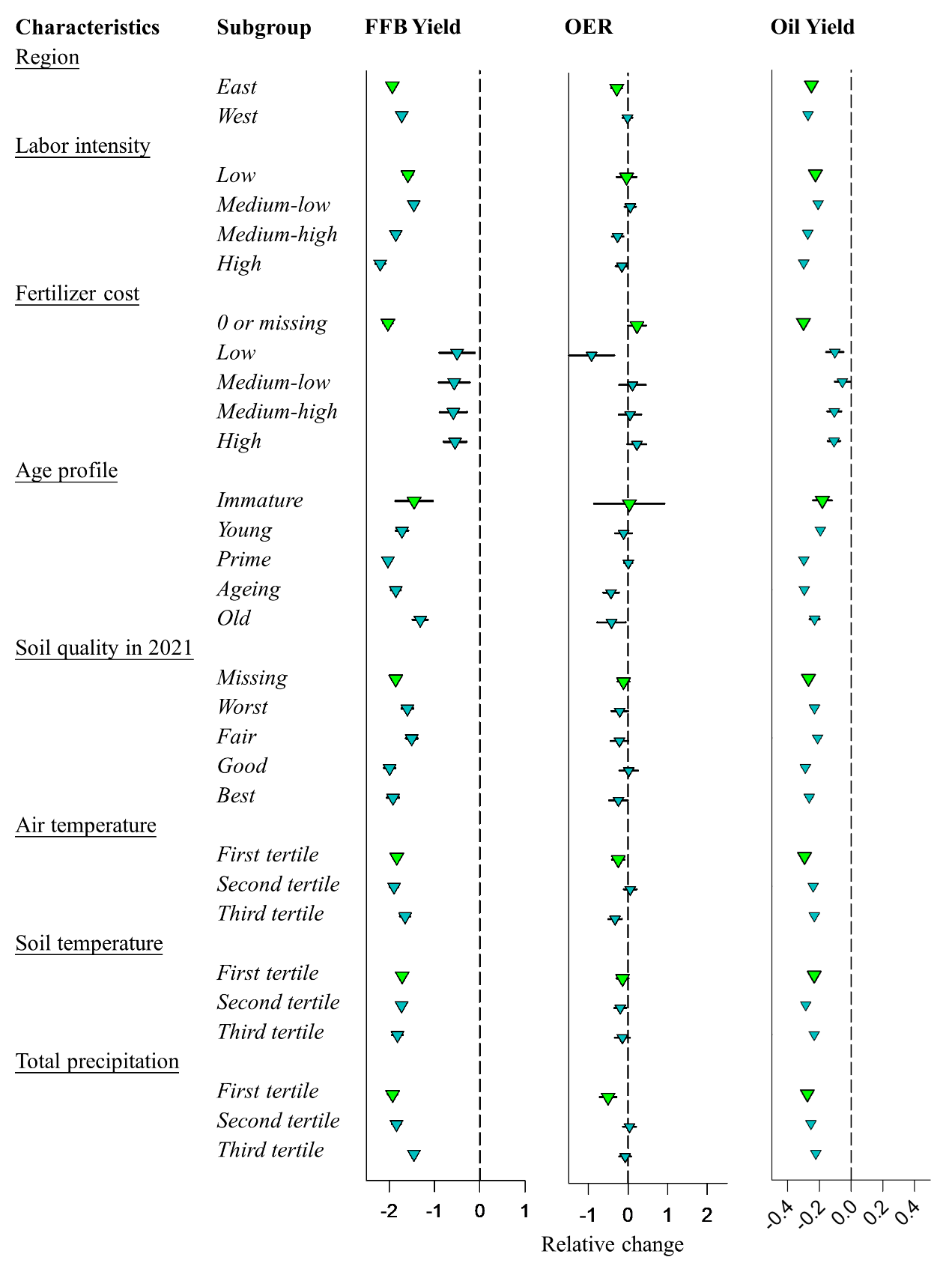


Fig. S8.

**Cumulative associations between the FFB yield (t/ha, in 0-23 lagged months), average OER (%, in 0-10 lagged months), and oil yield (t/ha, in 0-23 lagged months) and Niño 1+2 exposure at the value of -1 stratified by characteristics of study estates.** Notes: (1) Abbreviations: FFB, fresh fruit bunch; OER, oil extraction rate. (2) For the definitions and data sources of each variable, refer to Extended Data Table S2. (3) Reference groups are the first subgroup of each characteristic with a different color.


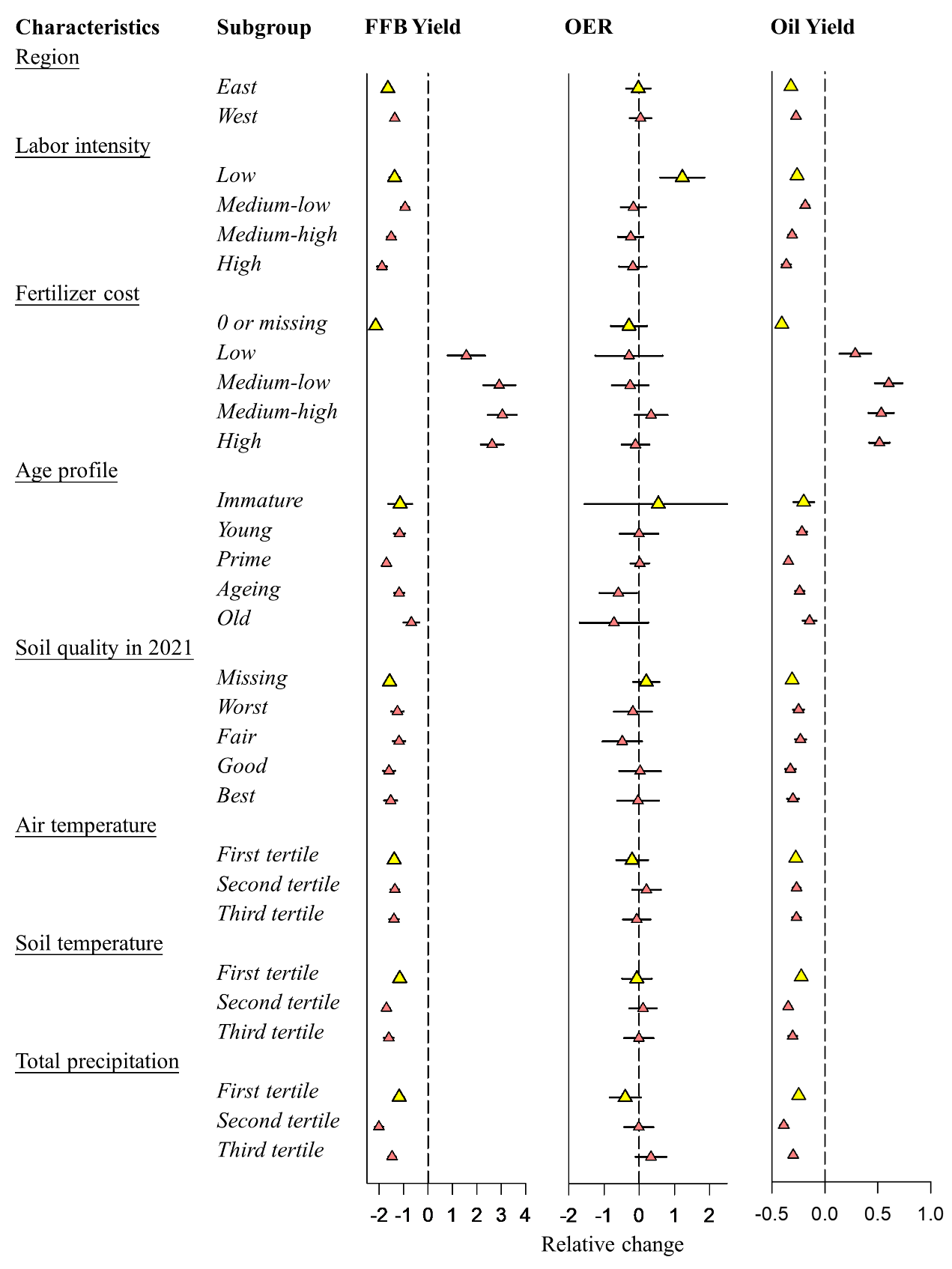


Fig. S9.

**Cumulative associations between the FFB yield (t/ha, in 0-23 lagged months), average OER (%, in 0-10 lagged months), and oil yield (t/ha, in 0-23 lagged months) and Niño 3.4 exposure at the value of 2 stratified by characteristics of study estates.** Notes: (1) Abbreviations: FFB, fresh fruit bunch; OER, oil extraction rate. (2) For the definitions and data sources of each variable, refer to Extended Data Table S2. (3) Reference groups are the first subgroup of each characteristic with a different color.


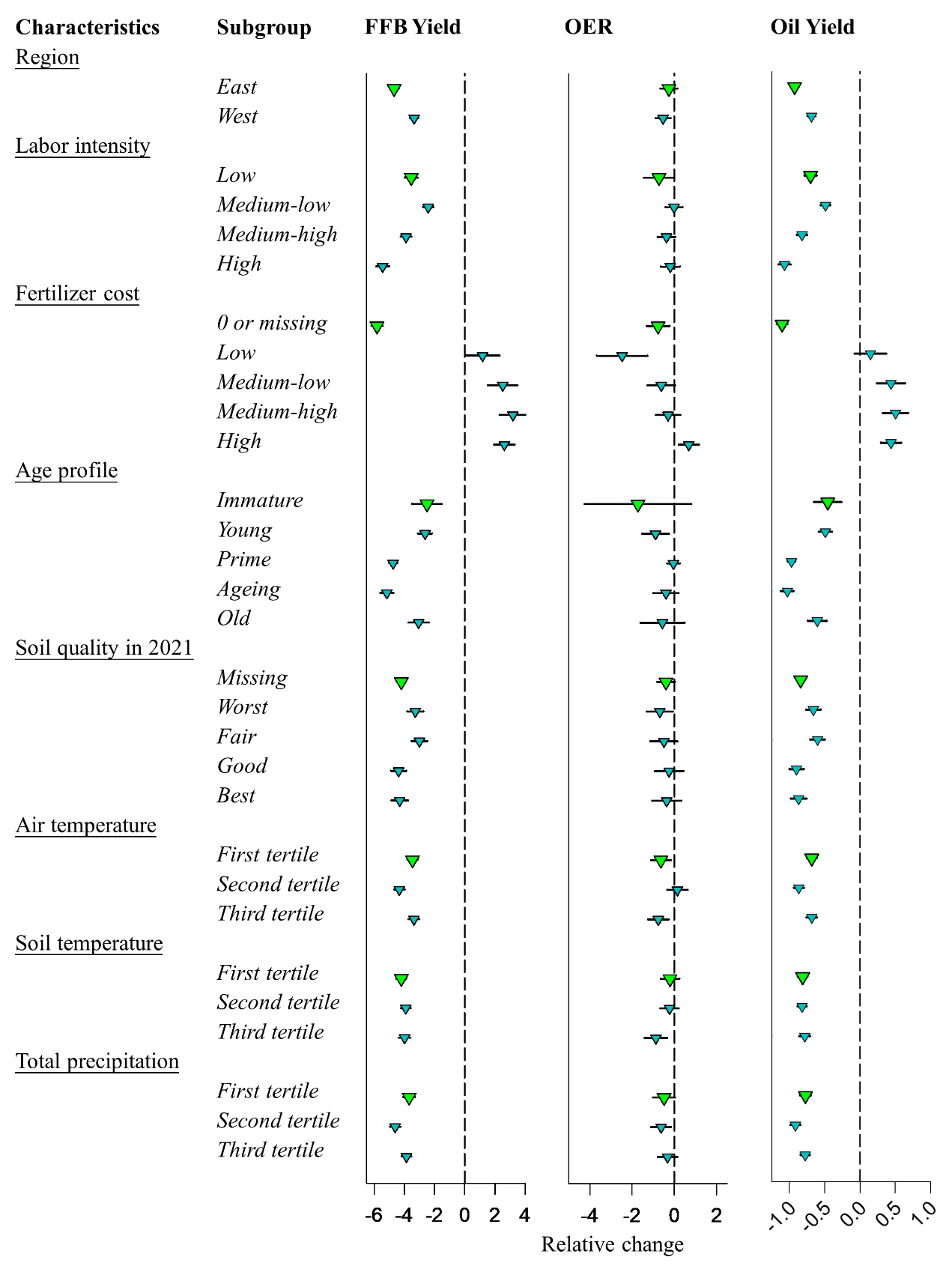


Fig. S10.

**Cumulative associations between the FFB yield (t/ha, in 0-23 lagged months), average OER (%, in 0-10 lagged months), and oil yield (t/ha, in 0-23 lagged months) and Niño 3.4 exposure at the value of -1 stratified by characteristics of study estates.** Notes: (1) Abbreviations: FFB, fresh fruit bunch; OER, oil extraction rate. (2) For the definitions and data sources of each variable, refer to Extended Data Table S2. (3) Reference groups are the first subgroup of each characteristic with a different color.


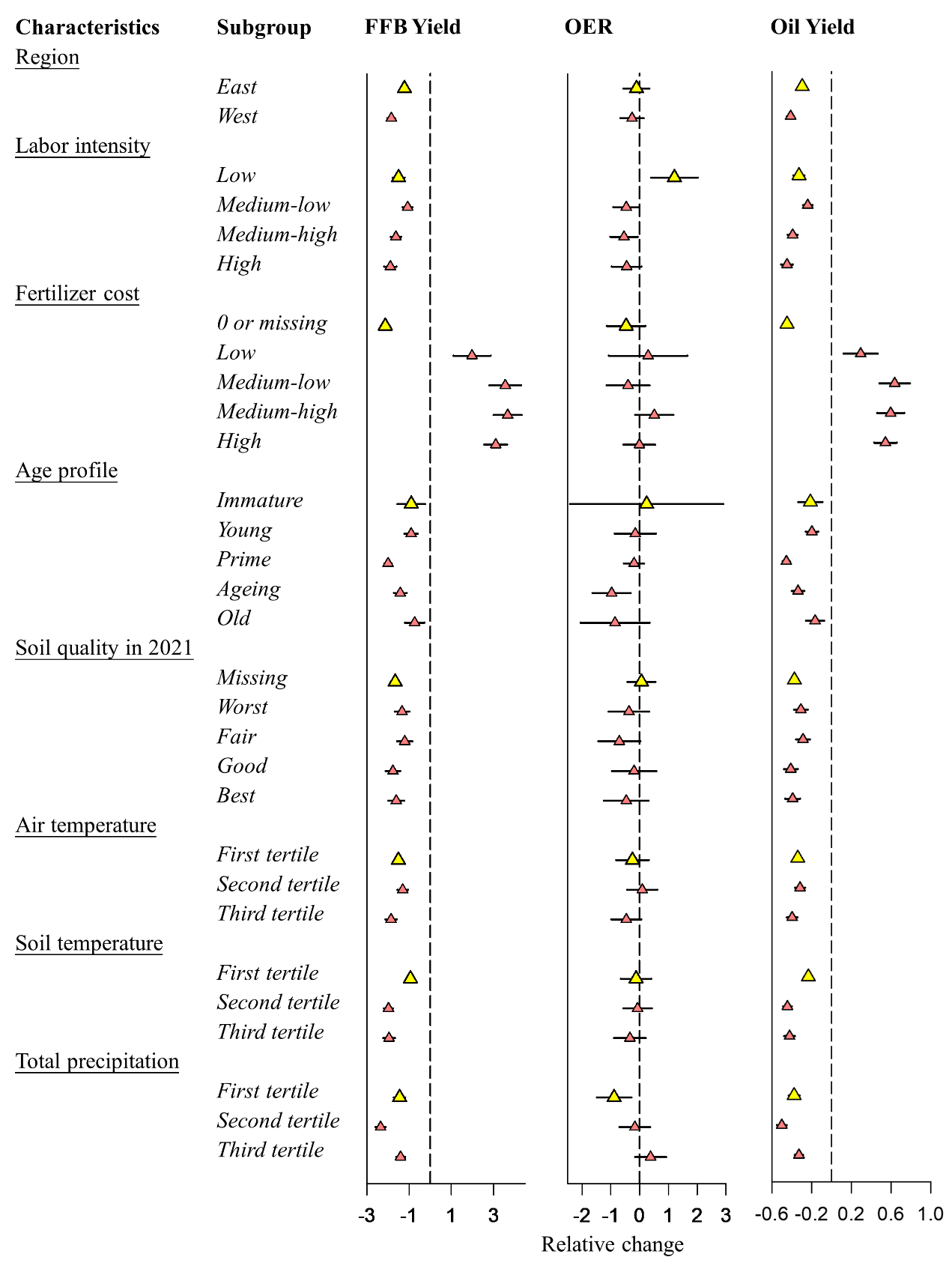


Fig. S11.

**Cumulative associations between the FFB yield (t/ha, in 0-23 lagged months), average OER (%, in 0-10 lagged months), and oil yield (t/ha, in 0-23 lagged months) and ONI exposure at the value of 2 stratified by characteristics of study estates.** Notes: (1) Abbreviations: ONI, Oceanic Niño Index; FFB, fresh fruit bunch; OER, oil extraction rate. (2) For the definitions and data sources of each variable, refer to Extended Data Table S2. (3) Reference groups are the first subgroup of each characteristic with a different color.


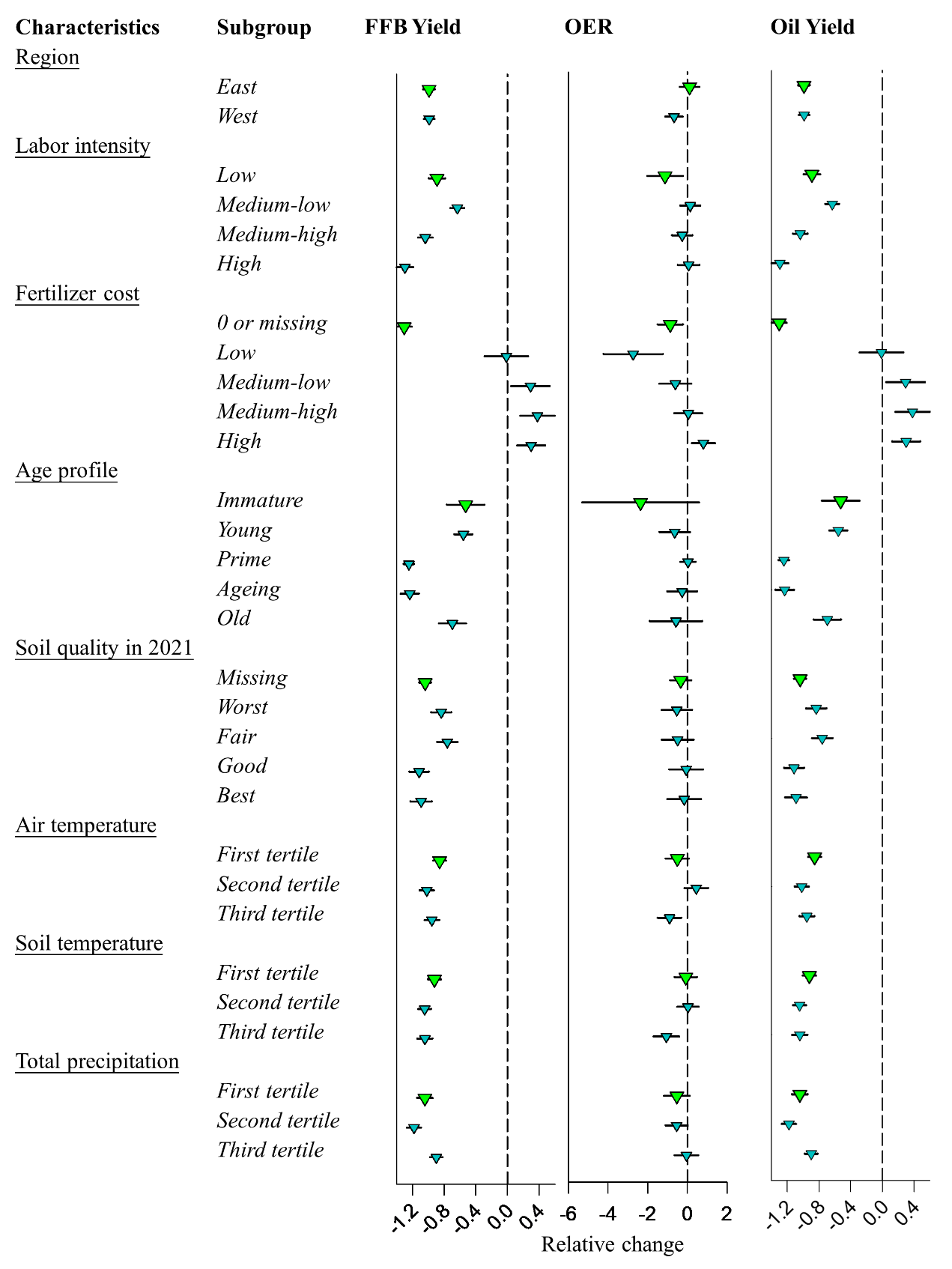


Fig. S12.

**Cumulative associations between the FFB yield (t/ha, in 0-23 lagged months), average OER (%, in 0-10 lagged months), and oil yield (t/ha, in 0-23 lagged months) and ONI exposure at the value of -1 stratified by characteristics of study estates.** Notes: (1) Abbreviations: ONI, Oceanic Niño Index; FFB, fresh fruit bunch; OER, oil extraction rate. (2) For the definitions and data sources of each variable, refer to Extended Data Table S2. (3) Reference groups are the first subgroup of each characteristic with a different color.


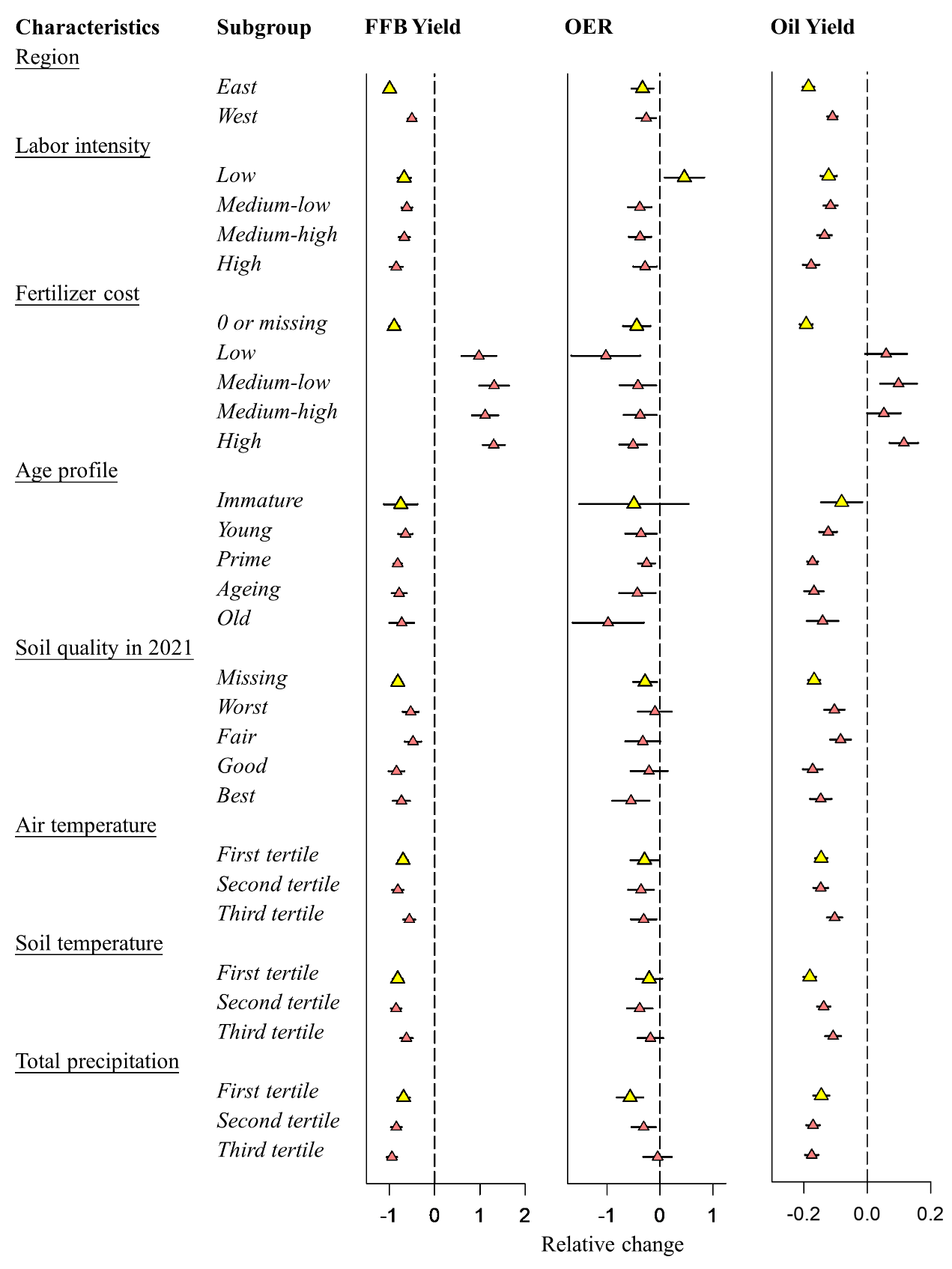


Fig. S13.

**Cumulative associations between the FFB yield (t/ha, in 0-23 lagged months), average OER (%, in 0-10 lagged months), and oil yield (t/ha, in 0-23 lagged months) and SOI exposure at the value of -2 (the value of negative SOI is 2) stratified by characteristics of study estates.** Notes: (1) Abbreviations: SOI, Southern Oscillation Index; FFB, fresh fruit bunch; OER, oil extraction rate. (2) For the definitions and data sources of each variable, refer to Extended Data Table S2. (3) Reference groups are the first subgroup of each characteristic with a different color.


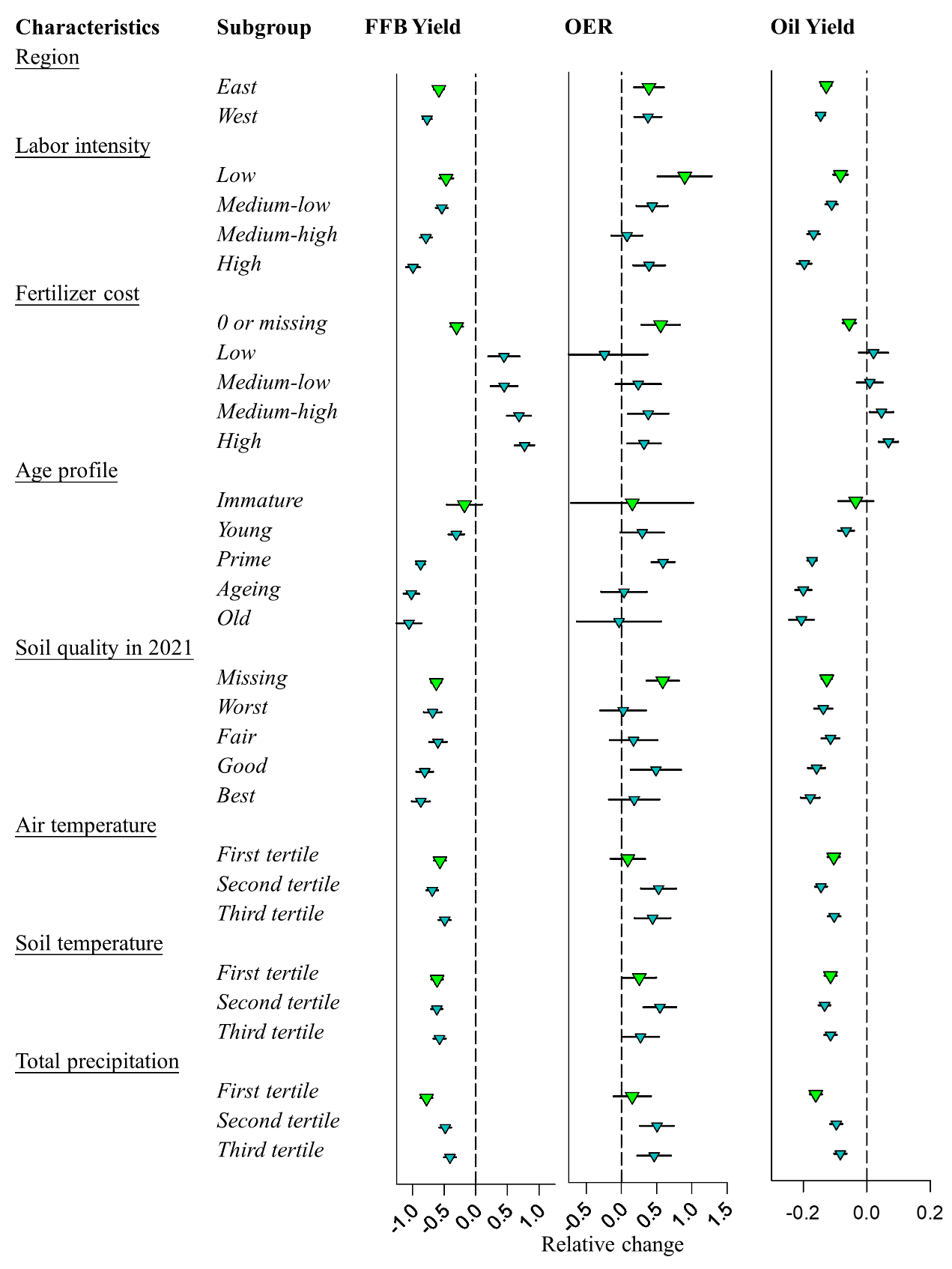


Fig. S14.

**Cumulative associations between the FFB yield (t/ha, in 0-23 lagged months), average OER (%, in 0-10 lagged months), and oil yield (t/ha, in 0-23 lagged months) and SOI exposure at the value of 1 (the value of negative SOI is -1) stratified by characteristics of study estates.** Notes: (1) Abbreviations: SOI, Southern Oscillation Index; FFB, fresh fruit bunch; OER, oil extraction rate. (2) For the definitions and data sources of each variable, refer to Extended Data Table S2. (3) Reference groups are the first subgroup of each characteristic with a different color.


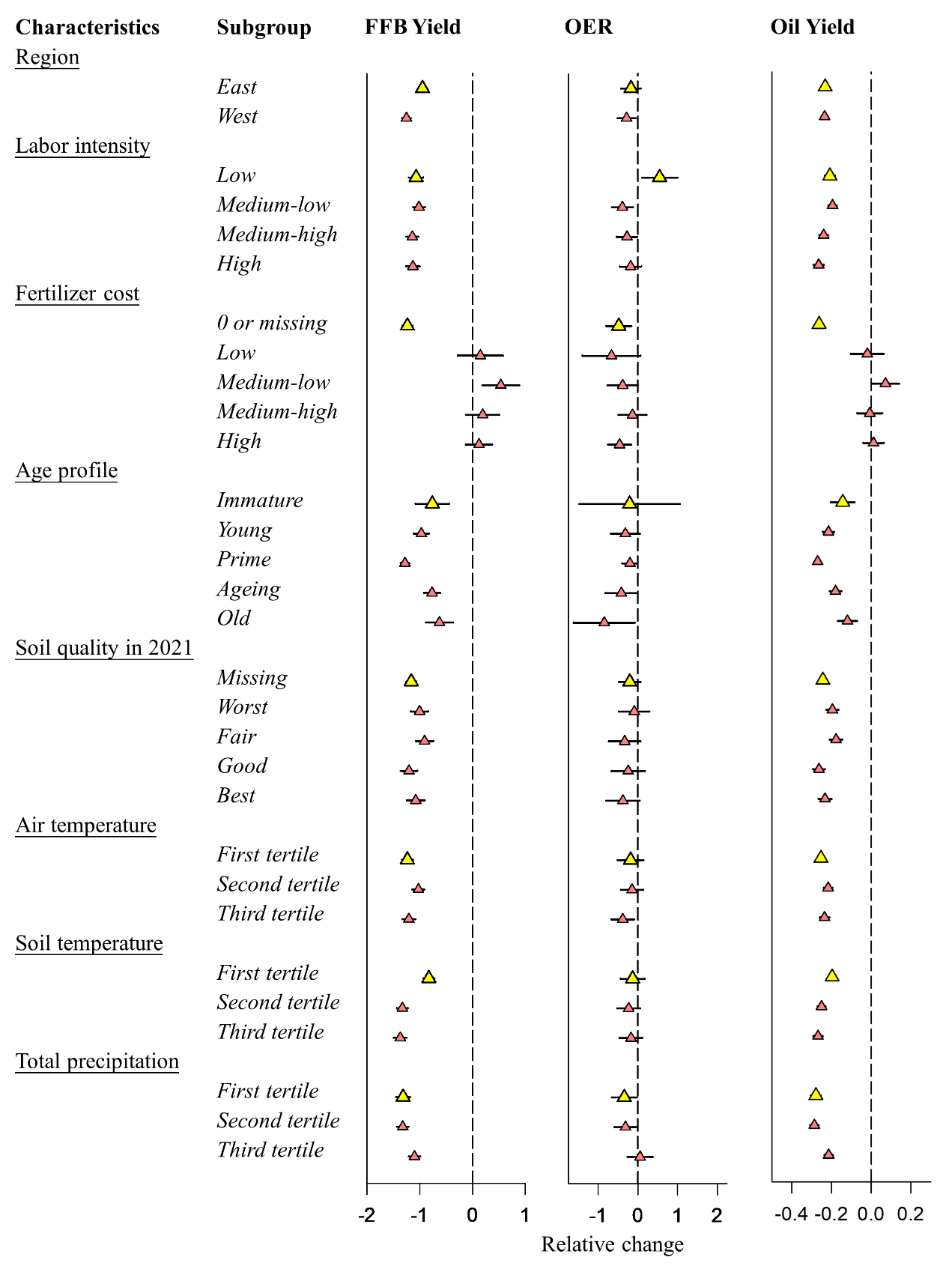


Fig. S15.

**Cumulative associations between the FFB yield (t/ha, in 0-23 lagged months), average OER (%, in 0-10 lagged months), and oil yield (t/ha, in 0-23 lagged months) and BEST exposure at the value of 2 stratified by characteristics of study estates.** Notes: (1) Abbreviations: BEST, Bivariate El Niño-Southern Oscillation (ENSO) Timeseries; FFB, fresh fruit bunch; OER, oil extraction rate. (2) For the definitions and data sources of each variable, refer to Extended Data Table S2. (3) Reference groups are the first subgroup of each characteristic with a different color.


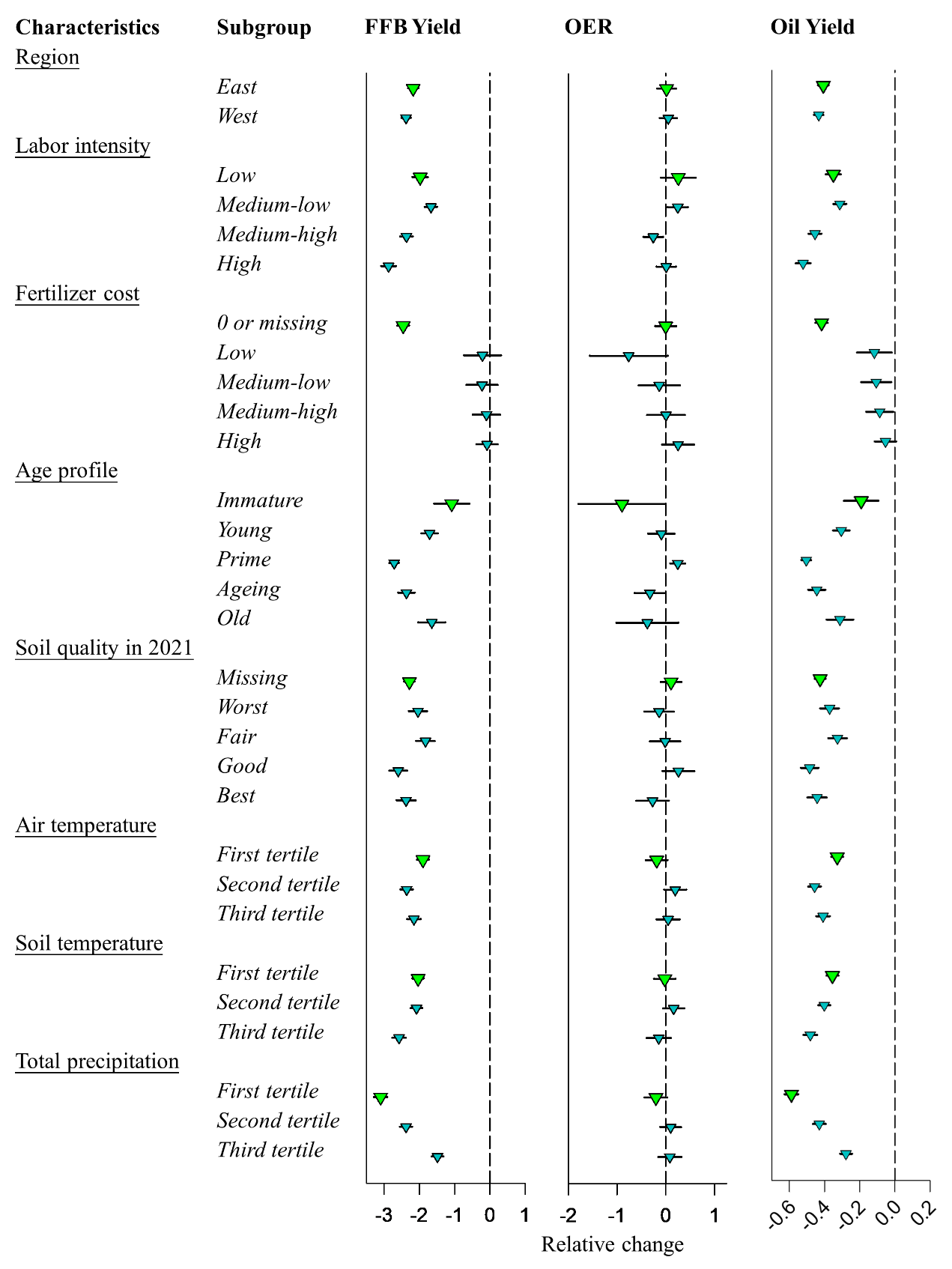


Fig. S16.

**Cumulative associations between the FFB yield (t/ha, in 0-23 lagged months), average OER (%, in 0-10 lagged months), and oil yield (t/ha, in 0-23 lagged months) and BEST exposure at the value of -1 stratified by characteristics of study estates.** Notes: (1) Abbreviations: BEST, Bivariate El Niño-Southern Oscillation (ENSO) Timeseries; FFB, fresh fruit bunch; OER, oil extraction rate. (2) For the definitions and data sources of each variable, refer to Extended Data Table S2. (3) Reference groups are the first subgroup of each characteristic with a different color.

Table S1.

**Detailed information on the time series of each ENSO indicator.**

| **Indicator** | **Definition and Source** |
| --- | --- |
| MEI | The Multivariate El Niño Index (MEI) is a monthly indicator that incorporates sea-surface temperature (SST), winds, sea-level pressure (SLP), and outgoing long-wave radiation into a single multivariable index. Data were last updated on 5 October 2023.  Source: <https://psl.noaa.gov/enso/mei/data/meiv2.data> |
| Niño 1+2 | SST anomalies (1991-2020 mean removed) averaged over the Niño 1+2 region, the smallest and eastern-most of the Niño SST regions (0-10°S, 90°W-80°W). It is based on ERSSTv5.  Source: <https://psl.noaa.gov/data/correlation/nina1.anom.data> |
| Niño 3.4 | SST anomalies (1991-2020 mean removed) averaged over the Niño 3.4 region (5°N-5°S, 170°W-120°W). It is based on ERSSTv5.  Source: <https://psl.noaa.gov/data/correlation/nina34.anom.data> |
| ONI | The Oceanic Niño Index (ONI) is a three-month running mean of ERSSTv5 SST anomalies in the Niño 3.4 region, based on changing base period which consists of multiple centered 30-year base periods. It is calculated from the Monthly NOAA ERSST V5 (at NOAA/CPC).  Source: <https://www.cpc.ncep.noaa.gov/data/indices/oni.ascii.txt> |
| SOI | The Southern Oscillation Index (SOI) is the difference between standardized Tahiti and standardized Darwin surface pressure values. Note the anomalies are departures from the 1981-2010 base period.  Source: <https://psl.noaa.gov/data/correlation/soi.data> |
| BEST | The Bivariate ENSO Timeseries (BEST) is calculated from combining a standardized SOI and a standardized Niño3.4 SST timeseries. HadISST1.1 is now used to calculate Niño 3.4 timeseries. Most recent data are based on the NOAA OI V2 SST dataset.  Source: <https://psl.noaa.gov/data/correlation/censo.data> |

Notes: (1) Abbreviations: ENSO, El Niño-Southern Oscillation; ERSST, Extended Reconstructed Sea Surface Temperature; HadISST, Hadley Centre Global Sea Ice and Sea Surface Temperature; NOAA, National Oceanic and Atmospheric Administration; CPC, Climate Prediction Center. (2) All the data are available from NOAA’s website at: <https://psl.noaa.gov/data/climateindices>.

Table S2.

**Detailed information on outcomes, control variables, and mediators.**

| **Indicator** | **Definition and Source** |
| --- | --- |
| *Panel A. Outcomes* | |
| FFB Yield | The fresh fruit bunch (FFB) yield is measured by the amount of FFB in metric ton (t) per hectare (ha) of harvested area in the current month for each estate. The monthly estate-level data are collected from different estates by the Malaysian Palm Oil Board (MPOB). |
| Average OER | The average oil extraction rate (OER) is the amount of oil obtained from all the FFB yield of each estate across multiple mills, measured in proportion, in the current month. The monthly estate-level data are collected from different mills and estates and calculated by the MPOB. |
| Oil Yield | This is the palm oil yield measured by the amount of palm oil in metric ton per hectare of harvested area in the current month for each estate. It is estimated by multiplying the FFB yield by the average OER. |
| *Panel B. Control variables* | |
| Labor intensity | It is based on the number of harvesters per hectare of harvested area in the current month for each estate. The monthly estate-level data are collected from different estates by the MPOB. |
| Phosphate rock | It is the average “free on board” (FOB) price of phosphate rock in USD in North America per metric ton in the past 12 months. The monthly data are obtained from the World Bank Commodity Price Data (The Pink Sheet), which can be downloaded freely from <https://www.worldbank.org/en/research/commodity-markets>. |
| DAP | It is the average FOB spot price of diammonium phosphate in USD in US Gulf per metric ton in the past 12 months. The data are obtained from the same source as the previous variable. |
| TSP | It is the average import spot price of triple superphosphate in USD in US Gulf per metric ton in the past 12 months. The data are obtained from the same source as the previous variable. |
| Urea | It is the average FOB spot price of prilled urea in USD in the Middle East beginning in March 2022 and previously in Black Sea per metric ton in the past 12 months. The data are obtained from the same source as the previous variable. |
| Potash | It is the average “cost and freight” (CFR) spot price of granular Potassium chloride (muriate of potash) in USD in Brazil from January 2020 and previously FOB price in USD in Vancouver per metric ton in the past 12 months. The data are obtained from the same source as the previous variable. |
| CPO | The average price of crude palm oil (CPO) in USD per metric ton in the past 12 months. It is calculated based on the monthly data obtained from MPOB in Malaysian Ringgit (MYR) and the nominal exchange rate in each month obtained below. |
| NER | It is the average nominal exchange rate (NER) in the past 12 months. We obtained the “middle rate” (between “buying” and “selling”) in each month from the Central Bank of Malaysia (<https://www.bnm.gov.my/web/guest/exchange-rates>) for the calculation. |
| CPI | It is the average commodity price index (CPI) in the past 12 months obtained from the World Bank Commodity Price Data (The Pink Sheet). |
| Soil quality | It is the quality of soil measured by MPOB for each estate in 2021. The proportion of each type of soil (including “alluvial”, “inland”, “shallow peat”, “laterite”, “acid sulphate”, “deep peat” and “sandy” soils) is measured, and then a score between 1 and 7 is calculated for each estate, which is the weighted average of the score of each type (i.e., “alluvial” is the best and have a score of 7, while “sandy” is the worst and have a score of 1). |
| AAP | It is the average age profile (AAP) of oil palm trees in the previous year for each estate. The yearly estate-level data are collected from different estates by the MPOB. Immature, young, prime, ageing, and old ages refer to 0-3, 4-8, 9-18, 19-23, and over 24 years. |
| Fertilizer cost | It is the total fertilizer and application cost in the current year for each estate, which includes fertilizer purchases, labor costs to apply fertilizer, and machinery use and maintenance for fertilizing activities. Foliar analysis costs are excluded from the total cost. The yearly estate-level data are collected from different estates by the MPOB. For the year of 2023, all estates are categorized into the “0 or missing” group, as they have not reported their fertilizer costs yet. Before 2023, estates are only categorized into the “0 or missing” group when they do not report any costs—in most cases, they do not incur any costs. |
| COVID cases | The number of confirmed COVID-19 infections in the current month for each estate’s state is obtained from Ministry of Health Malaysia (<https://covidnow.moh.gov.my>). |
| Lockdown | It is a binary variable indicating whether any lockdowns are conducted in the current month in the estate’s state. The variable is constructed based on governmental announcements from official websites, news, and articles on the Rahmat Lim & Partners (<https://www.rahmatlim.com>). |
| *Panel C. Mediators* | |
| Air temperature | The data are available from the Copernicus Climate Data Store (<https://cds.climate.copernicus.eu/cdsapp#!/home>). We obtained European Centre for Medium-Range Weather Forecasts (ECMWF)’s ERA5-Land monthly averaged data from 1950 to present, and then matched the address of each estate to the temperature of air at 2m above the surface of land, sea or in-land waters in the nearest 9 km by 9 km grid. We calculate the 24-month average air temperature in the current month and the past 23 months. |
| Soil temperature | The data are available from the previous source. The address of each estate is matched to the temperature of the soil in layer 1 (0-7 cm, with 0 cm being the surface) of the ECMWF Integrated Forecasting System in the nearest 9 km by 9 km grid. The soil temperature is set at the middle of the layer, and heat transfer is calculated at the interfaces between them. It is assumed that there is no heat transfer out of the bottom of the lowest layer. We calculate the 24-month average soil temperature in the current month and the past 23 months. |
| Total precipitation | The data are available from the previous source. The address of each estate is matched to the accumulated liquid and frozen water, including rain and snow, that falls to the Earth’s surface in the nearest 9 km by 9 km grid. It is the sum of large-scale precipitation (that precipitation which is generated by large-scale weather patterns, such as troughs and cold fronts) and convective precipitation (generated by convection which occurs when air at lower levels in the atmosphere is warmer and less dense than the air above, so it rises). It does not include fog, dew or the precipitation that evaporates in the atmosphere before it lands at the surface of the Earth. It is the depth the water would have if it were spread evenly over the grid box. We calculate the 24-month average daily precipitation in the current month and the past 23 months. |

Table S3.

**Characteristics of estates based on the study sample (N = 424,185).**

| **Characteristic** | **Mean** | **SD** | **Min** | **Max** |
| --- | --- | --- | --- | --- |
| FFB Yield (t/ha) | 1.36 | 0.69 | 0.00 | 5.99 |
| Average OER (%) | 18.71 | 4.01 | 0.00 | 30.00 |
| Oil Yield (t/ha) | 0.26 | 0.14 | 0.00 | 1.55 |
| Labor intensity (persons/hectare) | 0.04 | 0.05 | 0.00 | 5.41 |
| Low (N = 105,974) | 0.01 | 0.01 | 0.00 | 0.02 |
| Medium-low (N = 106,066) | 0.03 | <0.01 | 0.02 | 0.04 |
| Medium-high (N = 106,098) | 0.04 | <0.01 | 0.04 | 0.05 |
| High (N = 106,047) | 0.08 | 0.08 | 0.05 | 5.41 |
| Phosphate rock (USD/t) | 137.10 | 82.64 | 70.75 | 345.00 |
| DAP (USD/t) | 448.96 | 172.78 | 238.16 | 954.00 |
| TSP (USD/t) | 405.66 | 160.66 | 239.00 | 856.00 |
| Urea (USD/t) | 379.38 | 189.53 | 142.63 | 925.00 |
| Potash (USD/t) | 379.38 | 245.40 | 206.50 | 1,202.00 |
| CPO (USD/t) | 753.27 | 272.71 | 434.50 | 1,634.26 |
| NER | 4.19 | 0.21 | 3.56 | 4.73 |
| CPI (2010 = 100) | 88.59 | 26.91 | 46.24 | 158.78 |
| Soil quality score, 1-7, higher is better (N = 231,481) | 5.24 | 1.58 | 1.00 | 7.00 |
| Missing (N = 192,704) | -- | -- | -- | -- |
| Worst (N = 57,061) | 2.90 | 1.13 | 1.00 | 4.27 |
| Fair (N = 59,189) | 5.19 | 0.44 | 4.29 | 6.00 |
| Good (N = 57,782) | 6.00 | 0.01 | 6.00 | 6.10 |
| Best (N = 57,449) | 6.83 | 0.27 | 6.10 | 7.00 |
| AAP (years) last year | 13.18 | 6.71 | 0.00 | 45.00 |
| Immature (N = 29,826) | 2.23 | 1.25 | 0.00 | 4.00 |
| Young (N = 94,135) | 6.57 | 1.43 | 4.00 | 9.00 |
| Prime (N = 213,592) | 13.67 | 2.82 | 9.00 | 19.00 |
| Ageing (N = 60,599) | 21.01 | 1.39 | 19.00 | 24.00 |
| Old (N = 26,033) | 27.37 | 3.72 | 24.00 | 45.00 |
| Fertilizer cost (thousand MYR) this year (N = 239,894) | 1,135.28 | 2,282.65 | 0.00 | 78,891.70 |
| 0 or missing (N = 184,291) | 0.00 | 0.00 | 0.00 | 0.00 |
| Low (N = 51,454) | 42.06 | 32.91 | 0.00 | 99.26 |
| Medium-low (N = 61,214) | 193.89 | 65.08 | 99.28 | 327.46 |
| Medium-high (N = 63,137) | 649.54 | 233.54 | 327.51 | 1,159.53 |
| High (N = 64,089) | 3,390.65 | 3,509.97 | 1,160.13 | 78,891.70 |
| COVID-19 cases (thousand) | 3.83 | 12.74 | 0.00 | 243.48 |
| Lockdown | 0.12 | 0.32 | 0.00 | 1.00 |
| Yes (N = 49,532) | 1.00 | 0.00 | 1.00 | 1.00 |
| No (N = 374,653) | 0.00 | 0.00 | 0.00 | 0.00 |
| Air temperature (°C) | 26.24 | 0.86 | 18.65 | 30.40 |
| Soil temperature (°C) | 27.06 | 0.97 | 19.49 | 33.85 |
| Total precipitation (mm/day) | 7.09 | 3.32 | 0.06 | 33.01 |

Notes: (1) Abbreviations: SD, standard deviation; FFB, fresh fruit bunch; t, metric ton; OER, oil extraction rate; USD, US dollar; DAP, diammonium phosphate; TSP, triple superphosphate; CPO, crude palm oil; NER, nominal exchange rate; CPI, commodity price index; AAP, average age profile; MYR, Malaysian Ringgit; °C, degree Celsius; mm, millimeter. (2) For detailed definitions and data sources, please refer to Table S2.

Table S4.

**Cumulative associations between the FFB yield, average OER, and oil yield and extreme levels of ENSO exposure stratified by estates’ region and labor intensity in the current month.**

| **Characteristic** | | **Region** | |  | **Labor intensity** | | | |
| --- | --- | --- | --- | --- | --- | --- | --- | --- |
| **Subgroup** | | **East** | **West** |  | **Low** | **Medium-low** | **Medium-high** | **High** |
| *Panel A. At the value of +2 for each indicator* | | | | | | | | |
| **FFB yield in 0-23 lagged months** | | | | | | | | |
|  | MEI | -2.34 (-2.50, -2.17) | -2.24 (-2.40, -2.09) |  | -2.01 (-2.25, -1.76) | -2.09 (-2.30, -1.89) | -2.41 (-2.61, -2.20) | -2.55 (-2.79, -2.32) |
|  | *P*-value | Ref. | 0.41 |  | Ref. | 0.60 | 0.01 | <0.01 |
|  | Niño 1+2 | -0.90 (-0.99, -0.81) | -1.20 (-1.28, -1.12) |  | -0.97 (-1.10, -0.84) | -0.87 (-0.99, -0.76) | -1.11 (-1.22, -1.01) | -1.20 (-1.32, -1.07) |
|  | *P*-value | Ref. | <0.01 |  | Ref. | 0.27 | 0.10 | 0.02 |
|  | Niño 3.4 | -1.64 (-1.79, -1.50) | -1.37 (-1.50, -1.24) |  | -1.37 (-1.58, -1.16) | -0.94 (-1.10, -0.78) | -1.51 (-1.68, -1.33) | -1.89 (-2.09, -1.68) |
|  | *P*-value | Ref. | 0.01 |  | Ref. | <0.01 | 0.33 | <0.01 |
|  | ONI | -1.23 (-1.42, -1.03) | -1.85 (-2.03, -1.67) |  | -1.51 (-1.79, -1.22) | -1.07 (-1.30, -0.84) | -1.63 (-1.88, -1.39) | -1.89 (-2.19, -1.60) |
|  | *P*-value | Ref. | <0.01 |  | Ref. | 0.02 | 0.51 | 0.06 |
|  | Negative SOI | -0.99 (-1.09, -0.89) | -0.50 (-0.59, -0.41) |  | -0.67 (-0.82, -0.52) | -0.61 (-0.74, -0.49) | -0.67 (-0.79, -0.54) | -0.85 (-0.99, -0.70) |
|  | *P*-value | Ref. | <0.01 |  | Ref. | 0.55 | 0.98 | 0.09 |
|  | BEST | -0.95 (-1.04, -0.85) | -1.25 (-1.34, -1.17) |  | -1.07 (-1.21, -0.93) | -1.01 (-1.13, -0.90) | -1.14 (-1.25, -1.03) | -1.13 (-1.26, -1.00) |
|  | *P*-value | Ref. | <0.01 |  | Ref. | 0.54 | 0.43 | 0.54 |
| **Average OER (%) in 0-10 lagged months** | | | | | | | | |
|  | MEI | 0.32 (-0.12, 0.77) | -0.49 (-0.90, -0.09) |  | 1.83 (1.03, 2.64) | -0.58 (-1.05, -0.10) | -0.92 (-1.39, -0.46) | -0.22 (-0.70, 0.27) |
|  | *P*-value | Ref. | 0.01 |  | Ref. | <0.01 | <0.01 | <0.01 |
|  | Niño 1+2 | -0.29 (-0.57, 0.00) | 0.17 (-0.08, 0.42) |  | 1.18 (0.68, 1.69) | -0.07 (-0.37, 0.22) | -0.28 (-0.57, 0.01) | -0.47 (-0.78, -0.16) |
|  | *P*-value | Ref. | 0.02 |  | Ref. | <0.01 | <0.01 | <0.01 |
|  | Niño 3.4 | -0.02 (-0.37, 0.32) | 0.04 (-0.27, 0.35) |  | 1.23 (0.60, 1.85) | -0.16 (-0.52, 0.19) | -0.24 (-0.60, 0.12) | -0.18 (-0.58, 0.21) |
|  | *P*-value | Ref. | 0.80 |  | Ref. | <0.01 | <0.01 | <0.01 |
|  | ONI | -0.11 (-0.56, 0.34) | -0.26 (-0.67, 0.14) |  | 1.21 (0.40, 2.03) | -0.46 (-0.92, -0.01) | -0.54 (-1.02, -0.06) | -0.45 (-0.97, 0.07) |
|  | *P*-value | Ref. | 0.62 |  | Ref. | <0.01 | <0.01 | <0.01 |
|  | Negative SOI | -0.33 (-0.54, -0.12) | -0.26 (-0.45, -0.07) |  | 0.46 (0.09, 0.84) | -0.38 (-0.61, -0.16) | -0.38 (-0.59, -0.16) | -0.28 (-0.51, -0.06) |
|  | *P*-value | Ref. | 0.62 |  | Ref. | <0.01 | <0.01 | <0.01 |
|  | BEST | -0.17 (-0.42, 0.08) | -0.29 (-0.51, -0.06) |  | 0.55 (0.10, 1.00) | -0.39 (-0.66, -0.12) | -0.28 (-0.53, -0.02) | -0.19 (-0.46, 0.09) |
|  | *P*-value | Ref. | 0.51 |  | Ref. | <0.01 | <0.01 | 0.01 |
| **Oil yield in 0-23 lagged months** | | | | | | | | |
|  | MEI | -0.38 (-0.41, -0.34) | -0.38 (-0.41, -0.35) |  | -0.33 (-0.38, -0.29) | -0.36 (-0.40, -0.31) | -0.43 (-0.47, -0.39) | -0.39 (-0.44, -0.35) |
|  | *P*-value | Ref. | 0.70 |  | Ref. | 0.47 | <0.01 | 0.08 |
|  | Niño 1+2 | -0.25 (-0.27, -0.23) | -0.24 (-0.25, -0.22) |  | -0.20 (-0.22, -0.17) | -0.19 (-0.21, -0.17) | -0.25 (-0.28, -0.23) | -0.29 (-0.32, -0.27) |
|  | *P*-value | Ref. | 0.29 |  | Ref. | 0.73 | <0.01 | <0.01 |
|  | Niño 3.4 | -0.32 (-0.35, -0.29) | -0.27 (-0.30, -0.25) |  | -0.26 (-0.31, -0.22) | -0.19 (-0.22, -0.15) | -0.31 (-0.35, -0.27) | -0.37 (-0.41, -0.32) |
|  | *P*-value | Ref. | 0.02 |  | Ref. | <0.01 | 0.10 | <0.01 |
|  | ONI | -0.30 (-0.33, -0.26) | -0.41 (-0.45, -0.38) |  | -0.33 (-0.38, -0.27) | -0.24 (-0.29, -0.19) | -0.39 (-0.44, -0.34) | -0.45 (-0.51, -0.39) |
|  | *P*-value | Ref. | <0.01 |  | Ref. | 0.02 | 0.10 | <0.01 |
|  | Negative SOI | -0.19 (-0.20, -0.17) | -0.11 (-0.13, -0.09) |  | -0.12 (-0.15, -0.10) | -0.12 (-0.14, -0.09) | -0.13 (-0.16, -0.11) | -0.18 (-0.2, -0.15) |
|  | *P*-value | Ref. | <0.01 |  | Ref. | 0.74 | 0.46 | <0.01 |
|  | BEST | -0.23 (-0.25, -0.21) | -0.23 (-0.25, -0.22) |  | -0.21 (-0.23, -0.18) | -0.19 (-0.22, -0.17) | -0.24 (-0.26, -0.22) | -0.26 (-0.29, -0.24) |
|  | *P*-value | Ref. | 0.83 |  | Ref. | 0.43 | 0.06 | <0.01 |
| *Panel B. At the value of -1 for each indicator* | | | | | | | | |
| **FFB yield in 0-23 lagged months** | | | | | | | | |
|  | MEI | -1.76 (-1.89, -1.64) | -1.91 (-2.03, -1.80) |  | -1.60 (-1.79, -1.42) | -1.45 (-1.61, -1.30) | -1.89 (-2.05, -1.74) | -2.14 (-2.32, -1.96) |
|  | *P*-value | Ref. | 0.09 |  | Ref. | 0.22 | 0.02 | <0.01 |
|  | Niño 1+2 | -1.93 (-2.00, -1.86) | -1.72 (-1.79, -1.66) |  | -1.59 (-1.69, -1.48) | -1.46 (-1.54, -1.37) | -1.85 (-1.94, -1.76) | -2.20 (-2.30, -2.09) |
|  | *P*-value | Ref. | <0.01 |  | Ref. | 0.05 | <0.01 | <0.01 |
|  | Niño 3.4 | -4.67 (-4.97, -4.37) | -3.35 (-3.63, -3.08) |  | -3.53 (-3.97, -3.10) | -2.41 (-2.76, -2.07) | -3.88 (-4.24, -3.52) | -5.43 (-5.86, -5.00) |
|  | *P*-value | Ref. | <0.01 |  | Ref. | <0.01 | 0.23 | <0.01 |
|  | ONI | -4.42 (-4.79, -4.05) | -4.52 (-4.86, -4.18) |  | -4.16 (-4.70, -3.62) | -2.86 (-3.30, -2.42) | -4.45 (-4.91, -3.99) | -5.79 (-6.33, -5.24) |
|  | *P*-value | Ref. | 0.69 |  | Ref. | <0.01 | 0.42 | <0.01 |
|  | Negative SOI | -0.58 (-0.66, -0.50) | -0.77 (-0.84, -0.70) |  | -0.47 (-0.58, -0.35) | -0.54 (-0.63, -0.44) | -0.79 (-0.88, -0.69) | -0.99 (-1.10, -0.88) |
|  | *P*-value | Ref. | <0.01 |  | Ref. | 0.36 | <0.01 | <0.01 |
|  | BEST | -2.17 (-2.32, -2.03) | -2.38 (-2.51, -2.24) |  | -1.98 (-2.19, -1.76) | -1.67 (-1.85, -1.49) | -2.37 (-2.54, -2.19) | -2.87 (-3.08, -2.67) |
|  | *P*-value | Ref. | 0.05 |  | Ref. | 0.03 | 0.01 | <0.01 |
| **Average OER (%) in 0-10 lagged months** | | | | | | | | |
|  | MEI | -0.40 (-0.65, -0.16) | -0.15 (-0.37, 0.06) |  | -0.76 (-1.20, -0.31) | 0.02 (-0.23, 0.28) | -0.21 (-0.46, 0.04) | -0.12 (-0.38, 0.14) |
|  | *P*-value | Ref. | 0.14 |  | Ref. | <0.01 | 0.04 | 0.02 |
|  | Niño 1+2 | -0.29 (-0.42, -0.15) | -0.01 (-0.14, 0.11) |  | -0.04 (-0.29, 0.21) | 0.06 (-0.08, 0.20) | -0.26 (-0.41, -0.12) | -0.16 (-0.31, 0.00) |
|  | *P*-value | Ref. | <0.01 |  | Ref. | 0.53 | 0.12 | 0.42 |
|  | Niño 3.4 | -0.25 (-0.65, 0.15) | -0.53 (-0.89, -0.17) |  | -0.73 (-1.45, 0.00) | -0.01 (-0.42, 0.40) | -0.37 (-0.79, 0.05) | -0.19 (-0.65, 0.27) |
|  | *P*-value | Ref. | 0.31 |  | Ref. | 0.09 | 0.41 | 0.22 |
|  | ONI | 0.11 (-0.38, 0.61) | -0.67 (-1.11, -0.22) |  | -1.13 (-2.03, -0.23) | 0.15 (-0.36, 0.65) | -0.26 (-0.78, 0.26) | 0.07 (-0.49, 0.62) |
|  | *P*-value | Ref. | 0.02 |  | Ref. | 0.02 | 0.10 | 0.03 |
|  | Negative SOI | 0.39 (0.18, 0.60) | 0.38 (0.18, 0.57) |  | 0.9 (0.51, 1.28) | 0.44 (0.21, 0.66) | 0.08 (-0.14, 0.30) | 0.39 (0.16, 0.62) |
|  | *P*-value | Ref. | 0.92 |  | Ref. | 0.04 | <0.01 | 0.03 |
|  | BEST | 0.02 (-0.18, 0.21) | 0.05 (-0.12, 0.23) |  | 0.26 (-0.10, 0.62) | 0.25 (0.04, 0.46) | -0.26 (-0.46, -0.06) | 0.01 (-0.19, 0.21) |
|  | *P*-value | Ref. | 0.79 |  | Ref. | 0.95 | 0.01 | 0.24 |
| **Oil yield in 0-23 lagged months** | | | | | | | | |
|  | MEI | -0.26 (-0.29, -0.24) | -0.33 (-0.35, -0.31) |  | -0.27 (-0.31, -0.24) | -0.23 (-0.26, -0.21) | -0.33 (-0.36, -0.30) | -0.31 (-0.35, -0.28) |
|  | *P*-value | Ref. | <0.01 |  | Ref. | 0.08 | 0.02 | 0.10 |
|  | Niño 1+2 | -0.25 (-0.26, -0.24) | -0.27 (-0.28, -0.26) |  | -0.23 (-0.24, -0.21) | -0.21 (-0.22, -0.20) | -0.27 (-0.29, -0.26) | -0.3 (-0.31, -0.28) |
|  | *P*-value | Ref. | <0.01 |  | Ref. | 0.09 | <0.01 | <0.01 |
|  | Niño 3.4 | -0.93 (-0.99, -0.87) | -0.69 (-0.74, -0.63) |  | -0.70 (-0.79, -0.61) | -0.49 (-0.56, -0.42) | -0.82 (-0.90, -0.75) | -1.07 (-1.16, -0.98) |
|  | *P*-value | Ref. | <0.01 |  | Ref. | <0.01 | 0.03 | <0.01 |
|  | ONI | -0.99 (-1.06, -0.92) | -0.99 (-1.05, -0.92) |  | -0.89 (-0.99, -0.79) | -0.63 (-0.72, -0.55) | -1.04 (-1.13, -0.94) | -1.29 (-1.40, -1.19) |
|  | *P*-value | Ref. | 0.95 |  | Ref. | <0.01 | 0.04 | <0.01 |
|  | Negative SOI | -0.13 (-0.14, -0.11) | -0.15 (-0.16, -0.13) |  | -0.08 (-0.11, -0.06) | -0.11 (-0.13, -0.09) | -0.17 (-0.19, -0.15) | -0.20 (-0.22, -0.17) |
|  | *P*-value | Ref. | 0.11 |  | Ref. | 0.07 | <0.01 | <0.01 |
|  | BEST | -0.41 (-0.44, -0.38) | -0.43 (-0.46, -0.41) |  | -0.35 (-0.39, -0.31) | -0.31 (-0.35, -0.28) | -0.45 (-0.49, -0.42) | -0.52 (-0.56, -0.48) |
|  | *P*-value | Ref. | 0.21 |  | Ref. | 0.20 | <0.01 | <0.01 |

Notes: (1) Abbreviations: ENSO, El Niño Southern Oscillation; MEI, multivariate El Niño index; ONI, oceanic Niño index; SOI, Southern Oscillation Index; BEST, Bivariate ENSO Timeseries; Ref., reference group. (2) *P*-values are for the differences in effect estimates across subgroups. The association of each ENSO measure with each outcome is computed as the effect of a given value of ENSO measure relative to the reference value (set at zero). (3) For detailed definitions and data sources, please refer to Table S2.

Table S5.

**Cumulative associations between the FFB yield, average OER, and oil yield and extreme levels of ENSO exposure stratified by estates’ annual fertilizer cost in the current year.**

| **Characteristic** | | **Fertilizer cost in the current year** | | | | |
| --- | --- | --- | --- | --- | --- | --- |
| **Subgroup** | | **0 or missing** | **Low** | **Medium-low** | **Medium-high** | **High** |
| *Panel A. At the value of +2 for each indicator* | | | | | | |
| **FFB yield in 0-23 lagged months** | | | | | | |
|  | MEI | -2.98 (-3.16, -2.81) | -1.27 (-2.06, -0.47) | -1.68 (-2.38, -0.99) | -1.89 (-2.51, -1.26) | -1.96 (-2.48, -1.44) |
|  | *P*-value | Ref. | <0.01 | <0.01 | <0.01 | <0.01 |
|  | Niño 1+2 | -1.13 (-1.22, -1.04) | 0.23 (-0.33, 0.78) | 0.59 (0.11, 1.08) | 0.41 (-0.02, 0.85) | 0.16 (-0.21, 0.53) |
|  | *P*-value | Ref. | <0.01 | <0.01 | <0.01 | <0.01 |
|  | Niño 3.4 | -2.14 (-2.30, -1.98) | 1.57 (0.81, 2.34) | 2.92 (2.26, 3.58) | 3.04 (2.45, 3.64) | 2.63 (2.16, 3.10) |
|  | *P*-value | Ref. | <0.01 | <0.01 | <0.01 | <0.01 |
|  | ONI | -2.14 (-2.36, -1.91) | 1.98 (1.09, 2.86) | 3.55 (2.79, 4.31) | 3.66 (3.00, 4.33) | 3.1 (2.56, 3.64) |
|  | *P*-value | Ref. | <0.01 | <0.01 | <0.01 | <0.01 |
|  | Negative SOI | -0.89 (-1.00, -0.78) | 0.98 (0.60, 1.36) | 1.32 (0.99, 1.64) | 1.12 (0.82, 1.41) | 1.31 (1.06, 1.55) |
|  | *P*-value | Ref. | <0.01 | <0.01 | <0.01 | <0.01 |
|  | BEST | -1.24 (-1.33, -1.14) | 0.15 (-0.28, 0.58) | 0.54 (0.18, 0.89) | 0.19 (-0.12, 0.51) | 0.12 (-0.13, 0.37) |
|  | *P*-value | Ref. | <0.01 | <0.01 | <0.01 | <0.01 |
| **Average OER (%) in 0-10 lagged months** | | | | | | |
|  | MEI | -0.11 (-0.65, 0.43) | -0.93 (-2.77, 0.91) | 0.44 (-0.56, 1.44) | 0.50 (-0.40, 1.39) | -0.51 (-1.25, 0.24) |
|  | *P*-value | Ref. | 0.40 | 0.34 | 0.25 | 0.40 |
|  | Niño 1+2 | 0.41 (0.03, 0.78) | 0.38 (-0.41, 1.17) | 0.40 (-0.05, 0.85) | -0.5 (-0.87, -0.12) | -0.43 (-0.74, -0.12) |
|  | *P*-value | Ref. | 0.95 | 0.97 | <0.01 | <0.01 |
|  | Niño 3.4 | -0.29 (-0.81, 0.22) | -0.29 (-1.25, 0.67) | -0.26 (-0.78, 0.26) | 0.34 (-0.13, 0.81) | -0.11 (-0.50, 0.28) |
|  | *P*-value | Ref. | 1.00 | 0.93 | 0.08 | 0.57 |
|  | ONI | -0.47 (-1.14, 0.20) | 0.30 (-1.07, 1.67) | -0.40 (-1.15, 0.35) | 0.51 (-0.16, 1.18) | -0.01 (-0.56, 0.55) |
|  | *P*-value | Ref. | 0.33 | 0.90 | 0.04 | 0.30 |
|  | Negative SOI | -0.44 (-0.70, -0.18) | -1.02 (-1.68, -0.37) | -0.42 (-0.76, -0.07) | -0.38 (-0.69, -0.06) | -0.51 (-0.77, -0.25) |
|  | *P*-value | Ref. | 0.10 | 0.92 | 0.75 | 0.71 |
|  | BEST | -0.48 (-0.80, -0.16) | -0.66 (-1.40, 0.07) | -0.38 (-0.77, 0.00) | -0.14 (-0.49, 0.22) | -0.46 (-0.75, -0.17) |
|  | *P*-value | Ref. | 0.65 | 0.70 | 0.15 | 0.92 |
| **Oil yield in 0-23 lagged months** | | | | | | |
|  | MEI | -0.47 (-0.51, -0.44) | -0.22 (-0.38, -0.07) | -0.30 (-0.44, -0.16) | -0.35 (-0.48, -0.23) | -0.40 (-0.51, -0.29) |
|  | *P*-value | Ref. | <0.01 | 0.02 | 0.08 | 0.23 |
|  | Niño 1+2 | -0.23 (-0.25, -0.21) | 0.01 (-0.10, 0.11) | 0.15 (0.05, 0.24) | 0.06 (-0.03, 0.14) | 0.02 (-0.05, 0.10) |
|  | *P*-value | Ref. | <0.01 | <0.01 | <0.01 | <0.01 |
|  | Niño 3.4 | -0.41 (-0.44, -0.38) | 0.29 (0.14, 0.43) | 0.60 (0.47, 0.73) | 0.53 (0.41, 0.65) | 0.51 (0.42, 0.61) |
|  | *P*-value | Ref. | <0.01 | <0.01 | <0.01 | <0.01 |
|  | ONI | -0.45 (-0.49, -0.40) | 0.29 (0.12, 0.47) | 0.64 (0.48, 0.79) | 0.60 (0.46, 0.73) | 0.54 (0.43, 0.66) |
|  | *P*-value | Ref. | <0.01 | <0.01 | <0.01 | <0.01 |
|  | Negative SOI | -0.19 (-0.21, -0.17) | 0.06 (-0.01, 0.13) | 0.10 (0.04, 0.16) | 0.05 (0.00, 0.11) | 0.12 (0.07, 0.16) |
|  | *P*-value | Ref. | <0.01 | <0.01 | <0.01 | <0.01 |
|  | BEST | -0.26 (-0.28, -0.24) | -0.02 (-0.10, 0.06) | 0.07 (0.00, 0.14) | -0.01 (-0.07, 0.06) | 0.01 (-0.04, 0.06) |
|  | *P*-value | Ref. | <0.01 | <0.01 | <0.01 | <0.01 |
| *Panel B. At the value of -1 for each indicator* | | | | | | |
| **FFB yield in 0-23 lagged months** | | | | | | |
|  | MEI | -2.11 (-2.25, -1.97) | -1.23 (-1.68, -0.78) | -1.49 (-1.88, -1.10) | -1.30 (-1.65, -0.96) | -1.09 (-1.37, -0.81) |
|  | *P*-value | Ref. | <0.01 | <0.01 | <0.01 | <0.01 |
|  | Niño 1+2 | -2.03 (-2.13, -1.93) | -0.50 (-0.89, -0.11) | -0.56 (-0.9, -0.22) | -0.58 (-0.88, -0.29) | -0.55 (-0.79, -0.30) |
|  | *P*-value | Ref. | <0.01 | <0.01 | <0.01 | <0.01 |
|  | Niño 3.4 | -5.80 (-6.16, -5.43) | 1.19 (0.04, 2.33) | 2.52 (1.53, 3.51) | 3.20 (2.32, 4.07) | 2.63 (1.94, 3.31) |
|  | *P*-value | Ref. | <0.01 | <0.01 | <0.01 | <0.01 |
|  | ONI | -6.43 (-6.89, -5.97) | 0.62 (-0.81, 2.05) | 1.93 (0.71, 3.16) | 2.41 (1.34, 3.48) | 1.82 (0.95, 2.68) |
|  | *P*-value | Ref. | <0.01 | <0.01 | <0.01 | <0.01 |
|  | Negative SOI | -0.30 (-0.40, -0.20) | 0.45 (0.20, 0.69) | 0.45 (0.24, 0.66) | 0.68 (0.49, 0.87) | 0.77 (0.61, 0.93) |
|  | *P*-value | Ref. | <0.01 | <0.01 | <0.01 | <0.01 |
|  | BEST | -2.46 (-2.63, -2.29) | -0.21 (-0.73, 0.32) | -0.22 (-0.67, 0.22) | -0.10 (-0.49, 0.29) | -0.08 (-0.39, 0.22) |
|  | *P*-value | Ref. | <0.01 | <0.01 | <0.01 | <0.01 |
| **Average OER (%) in 0-10 lagged months** | | | | | | |
|  | MEI | -0.42 (-0.72, -0.12) | -1.28 (-2.10, -0.46) | 0.09 (-0.37, 0.54) | 0.04 (-0.37, 0.45) | 0.43 (0.08, 0.78) |
|  | *P*-value | Ref. | 0.05 | 0.07 | 0.07 | <0.01 |
|  | Niño 1+2 | 0.22 (0.00, 0.45) | -0.92 (-1.50, -0.35) | 0.11 (-0.23, 0.45) | 0.05 (-0.23, 0.33) | 0.22 (-0.02, 0.46) |
|  | *P*-value | Ref. | <0.01 | 0.58 | 0.34 | 0.99 |
|  | Niño 3.4 | -0.77 (-1.32, -0.22) | -2.47 (-3.67, -1.27) | -0.61 (-1.27, 0.05) | -0.29 (-0.87, 0.29) | 0.69 (0.21, 1.17) |
|  | *P*-value | Ref. | 0.01 | 0.72 | 0.24 | <0.01 |
|  | ONI | -0.86 (-1.50, -0.22) | -2.73 (-4.23, -1.24) | -0.6 (-1.41, 0.20) | 0.05 (-0.66, 0.76) | 0.81 (0.23, 1.40) |
|  | *P*-value | Ref. | 0.02 | 0.63 | 0.06 | <0.01 |
|  | Negative SOI | 0.56 (0.28, 0.83) | -0.24 (-0.86, 0.37) | 0.24 (-0.08, 0.56) | 0.38 (0.09, 0.67) | 0.32 (0.08, 0.56) |
|  | *P*-value | Ref. | 0.02 | 0.14 | 0.39 | 0.20 |
|  | BEST | 0.00 (-0.22, 0.22) | -0.76 (-1.56, 0.04) | -0.13 (-0.56, 0.29) | 0.00 (-0.38, 0.39) | 0.25 (-0.08, 0.58) |
|  | *P*-value | Ref. | 0.07 | 0.58 | 0.98 | 0.21 |
| **Oil yield in 0-23 lagged months** | | | | | | |
|  | MEI | -0.32 (-0.34, -0.29) | -0.20 (-0.29, -0.12) | -0.24 (-0.32, -0.16) | -0.21 (-0.28, -0.14) | -0.2 (-0.26, -0.14) |
|  | *P*-value | Ref. | 0.01 | 0.06 | <0.01 | <0.01 |
|  | Niño 1+2 | -0.30 (-0.32, -0.29) | -0.10 (-0.16, -0.05) | -0.05 (-0.10, -0.01) | -0.11 (-0.15, -0.06) | -0.11 (-0.15, -0.07) |
|  | *P*-value | Ref. | <0.01 | <0.01 | <0.01 | <0.01 |
|  | Niño 3.4 | -1.10 (-1.18, -1.03) | 0.15 (-0.07, 0.37) | 0.44 (0.24, 0.64) | 0.51 (0.32, 0.69) | 0.44 (0.30, 0.58) |
|  | *P*-value | Ref. | <0.01 | <0.01 | <0.01 | <0.01 |
|  | ONI | -1.30 (-1.40, -1.21) | -0.01 (-0.29, 0.26) | 0.29 (0.05, 0.54) | 0.38 (0.16, 0.60) | 0.30 (0.12, 0.48) |
|  | *P*-value | Ref. | <0.01 | <0.01 | <0.01 | <0.01 |
|  | Negative SOI | -0.05 (-0.08, -0.03) | 0.02 (-0.02, 0.07) | 0.01 (-0.03, 0.05) | 0.05 (0.01, 0.08) | 0.07 (0.04, 0.10) |
|  | *P*-value | Ref. | <0.01 | 0.01 | <0.01 | <0.01 |
|  | BEST | -0.42 (-0.45, -0.38) | -0.12 (-0.22, -0.02) | -0.11 (-0.19, -0.02) | -0.09 (-0.16, -0.01) | -0.05 (-0.11, 0.01) |
|  | *P*-value | Ref. | <0.01 | <0.01 | <0.01 | <0.01 |

Notes: (1) Abbreviations: ENSO, El Niño Southern Oscillation; MEI, multivariate El Niño index; ONI, oceanic Niño index; SOI, Southern Oscillation Index; BEST, Bivariate ENSO Timeseries; Ref., reference group. (2) *P*-values are for the differences in effect estimates across subgroups. The association of each ENSO measure with each outcome is computed as the effect of a given value of ENSO measure relative to the reference value (set at zero). (3) For detailed definitions and data sources, please refer to Table S2.

Table S6.

**Cumulative associations between the FFB yield, average OER, and oil yield and extreme levels of ENSO exposure stratified by estates’ average age profile of oil palm trees in the previous year.**

| **Characteristic** | | **Average age profile in the previous year** | | | | |
| --- | --- | --- | --- | --- | --- | --- |
| **Subgroup** | | **Immature** | **Young** | **Prime** | **Ageing** | **Old** |
| *Panel A. At the value of +2 for each indicator* | | | | | | |
| **FFB yield in 0-23 lagged months** | | | | | | |
|  | MEI | -1.33 (-1.83, -0.84) | -2.35 (-2.61, -2.10) | -2.56 (-2.71, -2.40) | -1.73 (-2.00, -1.45) | -1.10 (-1.55, -0.64) |
|  | *P*-value | Ref. | <0.01 | <0.01 | 0.17 | 0.49 |
|  | Niño 1+2 | -0.75 (-1.03, -0.48) | -0.57 (-0.70, -0.43) | -1.19 (-1.27, -1.11) | -1.35 (-1.50, -1.20) | -1.20 (-1.44, -0.95) |
|  | *P*-value | Ref. | 0.24 | <0.01 | <0.01 | 0.02 |
|  | Niño 3.4 | -1.14 (-1.63, -0.65) | -1.17 (-1.40, -0.94) | -1.70 (-1.83, -1.58) | -1.18 (-1.39, -0.98) | -0.68 (-1.01, -0.36) |
|  | *P*-value | Ref. | 0.93 | 0.03 | 0.89 | 0.12 |
|  | ONI | -0.90 (-1.56, -0.25) | -0.91 (-1.23, -0.59) | -2.00 (-2.18, -1.82) | -1.42 (-1.72, -1.12) | -0.74 (-1.21, -0.28) |
|  | *P*-value | Ref. | 0.99 | <0.01 | 0.16 | 0.70 |
|  | Negative SOI | -0.75 (-1.12, -0.38) | -0.64 (-0.80, -0.49) | -0.81 (-0.91, -0.72) | -0.78 (-0.95, -0.62) | -0.73 (-1.00, -0.45) |
|  | *P*-value | Ref. | 0.62 | 0.73 | 0.87 | 0.93 |
|  | BEST | -0.76 (-1.08, -0.44) | -0.97 (-1.12, -0.83) | -1.28 (-1.36, -1.20) | -0.77 (-0.92, -0.61) | -0.63 (-0.88, -0.37) |
|  | *P*-value | Ref. | 0.25 | <0.01 | 0.98 | 0.52 |
| **Average OER (%) in 0-10 lagged months** | | | | | | |
|  | MEI | 2.14 (0.12, 4.16) | -0.06 (-0.70, 0.59) | -0.38 (-0.73, -0.03) | -0.51 (-1.24, 0.22) | -0.83 (-2.23, 0.57) |
|  | *P*-value | Ref. | 0.04 | 0.02 | 0.02 | 0.02 |
|  | Niño 1+2 | 1.29 (-0.07, 2.65) | -0.01 (-0.43, 0.42) | -0.05 (-0.27, 0.17) | -0.42 (-0.84, 0.00) | -0.33 (-1.11, 0.45) |
|  | *P*-value | Ref. | 0.08 | 0.06 | 0.02 | 0.04 |
|  | Niño 3.4 | 0.54 (-1.55, 2.64) | 0.00 (-0.55, 0.55) | 0.02 (-0.25, 0.28) | -0.59 (-1.12, -0.06) | -0.72 (-1.7, 0.26) |
|  | *P*-value | Ref. | 0.62 | 0.63 | 0.30 | 0.29 |
|  | ONI | 0.24 (-2.42, 2.91) | -0.15 (-0.87, 0.57) | -0.19 (-0.54, 0.16) | -0.97 (-1.63, -0.31) | -0.85 (-2.05, 0.35) |
|  | *P*-value | Ref. | 0.78 | 0.75 | 0.39 | 0.46 |
|  | Negative SOI | -0.49 (-1.53, 0.54) | -0.36 (-0.66, -0.06) | -0.25 (-0.41, -0.09) | -0.43 (-0.77, -0.08) | -0.98 (-1.66, -0.31) |
|  | *P*-value | Ref. | 0.81 | 0.65 | 0.91 | 0.44 |
|  | BEST | -0.21 (-1.48, 1.06) | -0.32 (-0.68, 0.05) | -0.20 (-0.40, -0.01) | -0.42 (-0.82, -0.01) | -0.85 (-1.62, -0.08) |
|  | *P*-value | Ref. | 0.87 | 0.99 | 0.76 | 0.40 |
| **Oil yield in 0-23 lagged months** | | | | | | |
|  | MEI | -0.23 (-0.32, -0.14) | -0.39 (-0.44, -0.34) | -0.43 (-0.46, -0.40) | -0.30 (-0.35, -0.24) | -0.22 (-0.30, -0.13) |
|  | *P*-value | Ref. | <0.01 | <0.01 | 0.21 | 0.84 |
|  | Niño 1+2 | -0.14 (-0.20, -0.09) | -0.15 (-0.17, -0.12) | -0.27 (-0.28, -0.25) | -0.30 (-0.33, -0.27) | -0.24 (-0.29, -0.19) |
|  | *P*-value | Ref. | 0.92 | <0.01 | <0.01 | 0.01 |
|  | Niño 3.4 | -0.20 (-0.30, -0.10) | -0.22 (-0.26, -0.17) | -0.35 (-0.37, -0.32) | -0.24 (-0.28, -0.20) | -0.14 (-0.21, -0.08) |
|  | *P*-value | Ref. | 0.77 | <0.01 | 0.48 | 0.35 |
|  | ONI | -0.21 (-0.34, -0.09) | -0.20 (-0.26, -0.14) | -0.46 (-0.49, -0.42) | -0.34 (-0.40, -0.28) | -0.17 (-0.26, -0.07) |
|  | *P*-value | Ref. | 0.82 | <0.01 | 0.08 | 0.54 |
|  | Negative SOI | -0.08 (-0.15, -0.02) | -0.12 (-0.15, -0.10) | -0.17 (-0.19, -0.16) | -0.17 (-0.20, -0.14) | -0.14 (-0.19, -0.09) |
|  | *P*-value | Ref. | 0.24 | 0.01 | 0.02 | 0.15 |
|  | BEST | -0.14 (-0.20, -0.08) | -0.22 (-0.24, -0.19) | -0.27 (-0.29, -0.25) | -0.18 (-0.21, -0.15) | -0.12 (-0.17, -0.07) |
|  | *P*-value | Ref. | 0.03 | <0.01 | 0.29 | 0.54 |
| *Panel B. At the value of -1 for each indicator* | | | | | | |
| **FFB yield in 0-23 lagged months** | | | | | | |
|  | MEI | -0.97 (-1.41, -0.53) | -1.50 (-1.69, -1.30) | -2.20 (-2.32, -2.09) | -1.68 (-1.88, -1.47) | -0.76 (-1.13, -0.39) |
|  | *P*-value | Ref. | 0.03 | <0.01 | <0.01 | 0.46 |
|  | Niño 1+2 | -1.45 (-1.86, -1.04) | -1.72 (-1.85, -1.58) | -2.03 (-2.09, -1.97) | -1.85 (-1.96, -1.74) | -1.31 (-1.48, -1.15) |
|  | *P*-value | Ref. | 0.22 | 0.01 | 0.06 | 0.55 |
|  | Niño 3.4 | -2.50 (-3.49, -1.51) | -2.62 (-3.09, -2.15) | -4.73 (-5.00, -4.46) | -5.15 (-5.59, -4.71) | -3.05 (-3.72, -2.37) |
|  | *P*-value | Ref. | 0.82 | <0.01 | <0.01 | 0.37 |
|  | ONI | -2.32 (-3.60, -1.03) | -2.63 (-3.23, -2.04) | -5.64 (-5.98, -5.30) | -5.73 (-6.30, -5.16) | -3.49 (-4.36, -2.63) |
|  | *P*-value | Ref. | 0.66 | <0.01 | <0.01 | 0.14 |
|  | Negative SOI | -0.18 (-0.46, 0.10) | -0.31 (-0.43, -0.18) | -0.87 (-0.94, -0.80) | -1.02 (-1.14, -0.89) | -1.05 (-1.25, -0.86) |
|  | *P*-value | Ref. | 0.41 | <0.01 | <0.01 | <0.01 |
|  | BEST | -1.08 (-1.59, -0.58) | -1.71 (-1.94, -1.48) | -2.71 (-2.85, -2.58) | -2.37 (-2.60, -2.13) | -1.65 (-2.03, -1.26) |
|  | *P*-value | Ref. | 0.03 | <0.01 | <0.01 | 0.08 |
| **Average OER (%) in 0-10 lagged months** | | | | | | |
|  | MEI | -0.98 (-2.09, 0.13) | -0.27 (-0.62, 0.08) | -0.04 (-0.23, 0.15) | -0.61 (-0.99, -0.24) | -0.54 (-1.25, 0.17) |
|  | *P*-value | Ref. | 0.23 | 0.10 | 0.54 | 0.51 |
|  | Niño 1+2 | 0.03 (-0.86, 0.92) | -0.11 (-0.33, 0.10) | 0.00 (-0.10, 0.11) | -0.43 (-0.63, -0.23) | -0.42 (-0.78, -0.06) |
|  | *P*-value | Ref. | 0.76 | 0.95 | 0.32 | 0.36 |
|  | Niño 3.4 | -1.72 (-4.25, 0.80) | -0.89 (-1.53, -0.24) | -0.04 (-0.34, 0.27) | -0.39 (-0.99, 0.21) | -0.56 (-1.61, 0.49) |
|  | *P*-value | Ref. | 0.53 | 0.19 | 0.32 | 0.41 |
|  | ONI | -2.36 (-5.3, 0.58) | -0.64 (-1.40, 0.13) | 0.03 (-0.35, 0.41) | -0.26 (-1.01, 0.49) | -0.57 (-1.90, 0.75) |
|  | *P*-value | Ref. | 0.27 | 0.11 | 0.18 | 0.28 |
|  | Negative SOI | 0.15 (-0.72, 1.02) | 0.29 (-0.02, 0.60) | 0.59 (0.43, 0.76) | 0.04 (-0.29, 0.36) | -0.04 (-0.63, 0.56) |
|  | *P*-value | Ref. | 0.76 | 0.33 | 0.80 | 0.73 |
|  | BEST | -0.90 (-1.79, 0.00) | -0.09 (-0.36, 0.18) | 0.25 (0.09, 0.40) | -0.32 (-0.64, -0.01) | -0.38 (-1.01, 0.26) |
|  | *P*-value | Ref. | 0.09 | 0.01 | 0.24 | 0.35 |
| **Oil yield in 0-23 lagged months** | | | | | | |
|  | MEI | -0.17 (-0.24, -0.10) | -0.23 (-0.27, -0.20) | -0.36 (-0.39, -0.34) | -0.28 (-0.32, -0.24) | -0.14 (-0.22, -0.07) |
|  | *P*-value | Ref. | 0.14 | <0.01 | 0.01 | 0.61 |
|  | Niño 1+2 | -0.18 (-0.24, -0.12) | -0.19 (-0.21, -0.18) | -0.30 (-0.31, -0.29) | -0.3 (-0.31, -0.28) | -0.23 (-0.26, -0.20) |
|  | *P*-value | Ref. | 0.67 | <0.01 | <0.01 | 0.14 |
|  | Niño 3.4 | -0.46 (-0.65, -0.26) | -0.49 (-0.58, -0.40) | -0.97 (-1.03, -0.92) | -1.03 (-1.12, -0.94) | -0.61 (-0.74, -0.47) |
|  | *P*-value | Ref. | 0.76 | <0.01 | <0.01 | 0.22 |
|  | ONI | -0.53 (-0.76, -0.29) | -0.56 (-0.67, -0.44) | -1.24 (-1.31, -1.18) | -1.23 (-1.35, -1.12) | -0.69 (-0.87, -0.52) |
|  | *P*-value | Ref. | 0.83 | <0.01 | <0.01 | 0.26 |
|  | Negative SOI | -0.03 (-0.09, 0.02) | -0.07 (-0.09, -0.04) | -0.17 (-0.19, -0.16) | -0.20 (-0.23, -0.17) | -0.21 (-0.24, -0.17) |
|  | *P*-value | Ref. | 0.33 | <0.01 | <0.01 | <0.01 |
|  | BEST | -0.19 (-0.29, -0.09) | -0.30 (-0.35, -0.26) | -0.50 (-0.53, -0.48) | -0.44 (-0.49, -0.40) | -0.31 (-0.39, -0.24) |
|  | *P*-value | Ref. | 0.04 | <0.01 | <0.01 | 0.06 |

Notes: (1) Abbreviations: ENSO, El Niño Southern Oscillation; MEI, multivariate El Niño index; ONI, oceanic Niño index; SOI, Southern Oscillation Index; BEST, Bivariate ENSO Timeseries; Ref., reference group. (2) *P*-values are for the differences in effect estimates across subgroups. The association of each ENSO measure with each outcome is computed as the effect of a given value of ENSO measure relative to the reference value (set at zero). (3) For detailed definitions and data sources, please refer to Table S2.

Table S7.

**Cumulative associations between the FFB yield, average OER, and oil yield and extreme levels of ENSO exposure stratified by estates’ soil quality in 2021.**

| **Characteristic** | | **Soil quality in 2021** | | | | |
| --- | --- | --- | --- | --- | --- | --- |
| **Subgroup** | | **Missing** | **Worst** | **Fair** | **Good** | **Best** |
| *Panel A. At the value of +2 for each indicator* | | | | | | |
| **FFB yield in 0-23 lagged months** | | | | | | |
|  | MEI | -2.37 (-2.54, -2.19) | -2.03 (-2.32, -1.73) | -2.04 (-2.33, -1.74) | -2.43 (-2.72, -2.14) | -2.27 (-2.57, -1.96) |
|  | *P*-value | Ref. | 0.05 | 0.06 | 0.70 | 0.58 |
|  | Niño 1+2 | -1.06 (-1.15, -0.96) | -0.99 (-1.15, -0.83) | -0.90 (-1.06, -0.74) | -1.18 (-1.34, -1.03) | -1.17 (-1.33, -1.00) |
|  | *P*-value | Ref. | 0.47 | 0.11 | 0.17 | 0.24 |
|  | Niño 3.4 | -1.57 (-1.72, -1.43) | -1.25 (-1.50, -1.00) | -1.19 (-1.44, -0.93) | -1.60 (-1.85, -1.35) | -1.53 (-1.80, -1.26) |
|  | *P*-value | Ref. | 0.03 | 0.01 | 0.85 | 0.78 |
|  | ONI | -1.67 (-1.87, -1.46) | -1.34 (-1.68, -0.99) | -1.21 (-1.57, -0.85) | -1.77 (-2.12, -1.42) | -1.61 (-1.99, -1.24) |
|  | *P*-value | Ref. | 0.11 | 0.03 | 0.62 | 0.81 |
|  | Negative SOI | -0.81 (-0.92, -0.71) | -0.53 (-0.70, -0.35) | -0.48 (-0.66, -0.29) | -0.84 (-1.02, -0.66) | -0.73 (-0.92, -0.54) |
|  | *P*-value | Ref. | 0.01 | <0.01 | 0.78 | 0.47 |
|  | BEST | -1.16 (-1.26, -1.07) | -1.00 (-1.17, -0.84) | -0.91 (-1.07, -0.74) | -1.20 (-1.36, -1.05) | -1.08 (-1.25, -0.91) |
|  | *P*-value | Ref. | 0.10 | 0.01 | 0.66 | 0.39 |
| **Average OER (%) in 0-10 lagged months** | | | | | | |
|  | MEI | 0.15 (-0.34, 0.64) | -0.32 (-1.01, 0.37) | -0.51 (-1.23, 0.20) | 0.15 (-0.61, 0.90) | -0.73 (-1.49, 0.04) |
|  | *P*-value | Ref. | 0.28 | 0.13 | 0.99 | 0.06 |
|  | Niño 1+2 | 0.21 (-0.09, 0.52) | -0.27 (-0.71, 0.16) | -0.44 (-0.89, 0.01) | 0.26 (-0.22, 0.74) | -0.54 (-1.02, -0.06) |
|  | *P*-value | Ref. | 0.07 | 0.02 | 0.86 | 0.01 |
|  | Niño 3.4 | 0.20 (-0.17, 0.58) | -0.18 (-0.72, 0.36) | -0.48 (-1.04, 0.08) | 0.03 (-0.56, 0.62) | -0.04 (-0.63, 0.56) |
|  | *P*-value | Ref. | 0.25 | 0.05 | 0.62 | 0.51 |
|  | ONI | 0.06 (-0.43, 0.55) | -0.37 (-1.07, 0.33) | -0.70 (-1.44, 0.03) | -0.19 (-0.96, 0.58) | -0.46 (-1.24, 0.32) |
|  | *P*-value | Ref. | 0.32 | 0.09 | 0.59 | 0.26 |
|  | Negative SOI | -0.28 (-0.51, -0.06) | -0.10 (-0.42, 0.22) | -0.33 (-0.66, 0.01) | -0.21 (-0.56, 0.14) | -0.55 (-0.90, -0.20) |
|  | *P*-value | Ref. | 0.35 | 0.84 | 0.72 | 0.21 |
|  | BEST | -0.20 (-0.48, 0.07) | -0.09 (-0.48, 0.29) | -0.33 (-0.73, 0.07) | -0.24 (-0.67, 0.18) | -0.38 (-0.80, 0.05) |
|  | *P*-value | Ref. | 0.64 | 0.60 | 0.88 | 0.51 |
| **Oil yield in 0-23 lagged months** | | | | | | |
|  | MEI | -0.40 (-0.43, -0.36) | -0.33 (-0.38, -0.27) | -0.34 (-0.40, -0.29) | -0.40 (-0.46, -0.35) | -0.37 (-0.43, -0.31) |
|  | *P*-value | Ref. | 0.04 | 0.13 | 0.83 | 0.51 |
|  | Niño 1+2 | -0.24 (-0.26, -0.22) | -0.21 (-0.25, -0.18) | -0.20 (-0.23, -0.16) | -0.27 (-0.30, -0.24) | -0.27 (-0.30, -0.23) |
|  | *P*-value | Ref. | 0.13 | 0.01 | 0.17 | 0.18 |
|  | Niño 3.4 | -0.31 (-0.34, -0.28) | -0.25 (-0.30, -0.20) | -0.23 (-0.28, -0.18) | -0.33 (-0.38, -0.28) | -0.30 (-0.36, -0.25) |
|  | *P*-value | Ref. | 0.04 | 0.01 | 0.58 | 0.83 |
|  | ONI | -0.37 (-0.42, -0.33) | -0.31 (-0.38, -0.24) | -0.29 (-0.36, -0.22) | -0.41 (-0.48, -0.34) | -0.39 (-0.47, -0.32) |
|  | *P*-value | Ref. | 0.11 | 0.04 | 0.37 | 0.69 |
|  | Negative SOI | -0.17 (-0.19, -0.15) | -0.10 (-0.14, -0.07) | -0.08 (-0.12, -0.05) | -0.17 (-0.20, -0.14) | -0.15 (-0.18, -0.11) |
|  | *P*-value | Ref. | <0.01 | <0.01 | 0.80 | 0.29 |
|  | BEST | -0.24 (-0.26, -0.23) | -0.20 (-0.23, -0.16) | -0.18 (-0.21, -0.15) | -0.26 (-0.29, -0.23) | -0.23 (-0.27, -0.20) |
|  | *P*-value | Ref. | 0.01 | <0.01 | 0.28 | 0.57 |
| *Panel B. At the value of -1 for each indicator* | | | | | | |
| **FFB yield in 0-23 lagged months** | | | | | | |
|  | MEI | -1.88 (-2.01, -1.75) | -1.71 (-1.93, -1.48) | -1.62 (-1.84, -1.39) | -1.94 (-2.15, -1.72) | -1.81 (-2.04, -1.57) |
|  | *P*-value | Ref. | 0.19 | 0.05 | 0.65 | 0.59 |
|  | Niño 1+2 | -1.85 (-1.93, -1.78) | -1.6 (-1.73, -1.47) | -1.5 (-1.63, -1.37) | -1.99 (-2.11, -1.87) | -1.92 (-2.05, -1.78) |
|  | *P*-value | Ref. | <0.01 | <0.01 | 0.06 | 0.43 |
|  | Niño 3.4 | -4.19 (-4.50, -3.89) | -3.27 (-3.79, -2.74) | -2.99 (-3.52, -2.46) | -4.37 (-4.89, -3.86) | -4.30 (-4.85, -3.74) |
|  | *P*-value | Ref. | <0.01 | <0.01 | 0.55 | 0.74 |
|  | ONI | -4.77 (-5.16, -4.39) | -3.77 (-4.43, -3.12) | -3.32 (-3.99, -2.64) | -4.95 (-5.6, -4.31) | -4.75 (-5.45, -4.06) |
|  | *P*-value | Ref. | 0.01 | <0.01 | 0.64 | 0.96 |
|  | Negative SOI | -0.62 (-0.70, -0.54) | -0.68 (-0.82, -0.54) | -0.60 (-0.74, -0.46) | -0.80 (-0.94, -0.67) | -0.87 (-1.01, -0.72) |
|  | *P*-value | Ref. | 0.48 | 0.75 | 0.02 | <0.01 |
|  | BEST | -2.28 (-2.44, -2.13) | -2.04 (-2.30, -1.78) | -1.83 (-2.09, -1.56) | -2.6 (-2.85, -2.35) | -2.38 (-2.65, -2.11) |
|  | *P*-value | Ref. | 0.11 | <0.01 | 0.03 | 0.55 |
| **Average OER (%) in 0-10 lagged months** | | | | | | |
|  | MEI | -0.32 (-0.59, -0.05) | -0.25 (-0.62, 0.13) | -0.25 (-0.64, 0.14) | -0.27 (-0.69, 0.14) | -0.28 (-0.69, 0.14) |
|  | *P*-value | Ref. | 0.76 | 0.78 | 0.85 | 0.86 |
|  | Niño 1+2 | -0.12 (-0.27, 0.03) | -0.21 (-0.42, 0.00) | -0.22 (-0.44, 0.00) | 0.01 (-0.22, 0.25) | -0.24 (-0.48, -0.01) |
|  | *P*-value | Ref. | 0.48 | 0.45 | 0.35 | 0.37 |
|  | Niño 3.4 | -0.39 (-0.82, 0.04) | -0.69 (-1.31, -0.07) | -0.49 (-1.14, 0.16) | -0.25 (-0.93, 0.44) | -0.36 (-1.05, 0.33) |
|  | *P*-value | Ref. | 0.44 | 0.80 | 0.73 | 0.95 |
|  | ONI | -0.33 (-0.87, 0.20) | -0.53 (-1.30, 0.24) | -0.49 (-1.29, 0.31) | -0.05 (-0.90, 0.79) | -0.16 (-1.01, 0.70) |
|  | *P*-value | Ref. | 0.68 | 0.75 | 0.59 | 0.73 |
|  | Negative SOI | 0.59 (0.36, 0.82) | 0.02 (-0.30, 0.35) | 0.17 (-0.17, 0.51) | 0.49 (0.13, 0.85) | 0.18 (-0.18, 0.54) |
|  | *P*-value | Ref. | 0.01 | 0.05 | 0.66 | 0.06 |
|  | BEST | 0.11 (-0.11, 0.32) | -0.14 (-0.44, 0.16) | -0.01 (-0.32, 0.3) | 0.26 (-0.07, 0.59) | -0.27 (-0.6, 0.06) |
|  | *P*-value | Ref. | 0.19 | 0.53 | 0.45 | 0.06 |
| **Oil yield in 0-23 lagged months** | | | | | | |
|  | MEI | -0.30 (-0.33, -0.28) | -0.28 (-0.32, -0.23) | -0.27 (-0.31, -0.23) | -0.31 (-0.35, -0.27) | -0.29 (-0.34, -0.25) |
|  | *P*-value | Ref. | 0.28 | 0.17 | 0.63 | 0.68 |
|  | Niño 1+2 | -0.27 (-0.28, -0.26) | -0.23 (-0.25, -0.21) | -0.21 (-0.23, -0.19) | -0.29 (-0.31, -0.27) | -0.26 (-0.28, -0.25) |
|  | *P*-value | Ref. | <0.01 | <0.01 | 0.06 | 0.68 |
|  | Niño 3.4 | -0.84 (-0.90, -0.78) | -0.66 (-0.77, -0.55) | -0.6 (-0.71, -0.50) | -0.90 (-1.00, -0.80) | -0.87 (-0.98, -0.76) |
|  | *P*-value | Ref. | <0.01 | <0.01 | 0.33 | 0.66 |
|  | ONI | -1.04 (-1.12, -0.96) | -0.84 (-0.96, -0.71) | -0.76 (-0.89, -0.63) | -1.11 (-1.24, -0.99) | -1.09 (-1.23, -0.95) |
|  | *P*-value | Ref. | 0.01 | <0.01 | 0.32 | 0.53 |
|  | Negative SOI | -0.13 (-0.14, -0.11) | -0.14 (-0.17, -0.11) | -0.11 (-0.14, -0.09) | -0.16 (-0.19, -0.13) | -0.18 (-0.21, -0.15) |
|  | *P*-value | Ref. | 0.50 | 0.49 | 0.05 | <0.01 |
|  | BEST | -0.43 (-0.46, -0.39) | -0.37 (-0.42, -0.32) | -0.33 (-0.38, -0.27) | -0.48 (-0.54, -0.43) | -0.44 (-0.5, -0.39) |
|  | *P*-value | Ref. | 0.09 | <0.01 | 0.05 | 0.59 |

Notes: (1) Abbreviations: ENSO, El Niño Southern Oscillation; MEI, multivariate El Niño index; ONI, oceanic Niño index; SOI, Southern Oscillation Index; BEST, Bivariate ENSO Timeseries; Ref., reference group. (2) *P*-values are for the differences in effect estimates across subgroups. The association of each ENSO measure with each outcome is computed as the effect of a given value of ENSO measure relative to the reference value (set at zero). (3) For detailed definitions and data sources, please refer to Table S2.

Table S8.

**Cumulative associations between the FFB yield, average OER, and oil yield and extreme levels of ENSO exposure stratified by estates’ exposure to the monthly average air temperature and total precipitation in the 0-23 lagged months.**

| **Characteristic** | | **Average air temperature in 0-23 lagged months** | | |  | **Average total precipitation in 0-23 lagged months** | | |
| --- | --- | --- | --- | --- | --- | --- | --- | --- |
| **Subgroup** | | **First tertile** | **Second tertile** | **Third tertile** |  | **First tertile** | **Second tertile** | **Third tertile** |
| *Panel A. At the value of +2 for each indicator* | | | | | | | | |
| **FFB yield in 0-23 lagged months** | | | | | | | | |
|  | MEI | -2.74 (-2.94, -2.54) | -2.47 (-2.68, -2.26) | -1.87 (-2.09, -1.65) |  | -2.15 (-2.36, -1.93) | -2.45 (-2.65, -2.26) | -2.22 (-2.43, -2.02) |
|  | *P*-value | Ref. | 0.07 | <0.01 |  | Ref. | 0.04 | 0.62 |
|  | Niño 1+2 | -1.34 (-1.44, -1.24) | -1.00 (-1.12, -0.89) | -1.10 (-1.22, -0.98) |  | -1.13 (-1.24, -1.02) | -0.98 (-1.08, -0.88) | -1.15 (-1.27, -1.03) |
|  | *P*-value | Ref. | <0.01 | <0.01 |  | Ref. | 0.05 | 0.79 |
|  | Niño 3.4 | -1.39 (-1.53, -1.24) | -1.36 (-1.54, -1.18) | -1.40 (-1.59, -1.20) |  | -1.18 (-1.38, -0.99) | -2.01 (-2.18, -1.84) | -1.48 (-1.63, -1.33) |
|  | *P*-value | Ref. | 0.82 | 0.93 |  | Ref. | <0.01 | 0.02 |
|  | ONI | -1.51 (-1.72, -1.30) | -1.30 (-1.55, -1.06) | -1.86 (-2.13, -1.58) |  | -1.46 (-1.72, -1.19) | -2.36 (-2.60, -2.11) | -1.41 (-1.62, -1.20) |
|  | *P*-value | Ref. | 0.21 | 0.05 |  | Ref. | <0.01 | 0.78 |
|  | Negative SOI | -0.70 (-0.80, -0.59) | -0.81 (-0.94, -0.69) | -0.55 (-0.69, -0.42) |  | -0.68 (-0.82, -0.55) | -0.84 (-0.96, -0.72) | -0.94 (-1.06, -0.82) |
|  | *P*-value | Ref. | 0.17 | 0.11 |  | Ref. | 0.09 | 0.01 |
|  | BEST | -1.23 (-1.34, -1.13) | -1.03 (-1.14, -0.91) | -1.21 (-1.33, -1.08) |  | -1.31 (-1.44, -1.19) | -1.32 (-1.43, -1.22) | -1.10 (-1.21, -0.99) |
|  | *P*-value | Ref. | 0.01 | 0.72 |  | Ref. | 0.91 | 0.01 |
| **Average OER (%) in 0-10 lagged months** | | | | | | | | |
|  | MEI | -0.24 (-0.83, 0.35) | -0.04 (-0.53, 0.46) | -0.23 (-0.74, 0.29) |  | -0.70 (-1.24, -0.16) | 0.11 (-0.38, 0.61) | 0.21 (-0.35, 0.78) |
|  | *P*-value | Ref. | 0.60 | 0.97 |  | Ref. | 0.03 | 0.02 |
|  | Niño 1+2 | 0.05 (-0.31, 0.40) | -0.03 (-0.35, 0.28) | -0.07 (-0.40, 0.26) |  | -0.42 (-0.78, -0.07) | 0.22 (-0.10, 0.54) | 0.12 (-0.20, 0.44) |
|  | *P*-value | Ref. | 0.74 | 0.64 |  | Ref. | 0.01 | 0.03 |
|  | Niño 3.4 | -0.20 (-0.65, 0.25) | 0.21 (-0.20, 0.61) | -0.07 (-0.46, 0.32) |  | -0.40 (-0.84, 0.04) | -0.01 (-0.43, 0.40) | 0.34 (-0.11, 0.78) |
|  | *P*-value | Ref. | 0.19 | 0.66 |  | Ref. | 0.21 | 0.02 |
|  | ONI | -0.25 (-0.82, 0.32) | 0.09 (-0.43, 0.62) | -0.46 (-0.98, 0.06) |  | -0.89 (-1.49, -0.28) | -0.17 (-0.70, 0.36) | 0.38 (-0.16, 0.92) |
|  | *P*-value | Ref. | 0.39 | 0.58 |  | Ref. | 0.08 | <0.01 |
|  | Negative SOI | -0.29 (-0.56, -0.02) | -0.36 (-0.60, -0.12) | -0.31 (-0.55, -0.07) |  | -0.57 (-0.82, -0.31) | -0.31 (-0.54, -0.08) | -0.04 (-0.32, 0.23) |
|  | *P*-value | Ref. | 0.71 | 0.93 |  | Ref. | 0.14 | 0.01 |
|  | BEST | -0.18 (-0.51, 0.14) | -0.15 (-0.43, 0.14) | -0.38 (-0.67, -0.09) |  | -0.34 (-0.66, -0.03) | -0.31 (-0.59, -0.04) | 0.06 (-0.26, 0.37) |
|  | *P*-value | Ref. | 0.87 | 0.38 |  | Ref. | 0.88 | 0.08 |
| **Oil yield in 0-23 lagged months** | | | | | | | | |
|  | MEI | -0.42 (-0.46, -0.38) | -0.43 (-0.48, -0.39) | -0.33 (-0.38, -0.29) |  | -0.36 (-0.41, -0.32) | -0.39 (-0.43, -0.35) | -0.4 (-0.45, -0.36) |
|  | *P*-value | Ref. | 0.60 | <0.01 |  | Ref. | 0.39 | 0.18 |
|  | Niño 1+2 | -0.29 (-0.31, -0.27) | -0.24 (-0.27, -0.22) | -0.23 (-0.25, -0.20) |  | -0.25 (-0.28, -0.23) | -0.21 (-0.23, -0.19) | -0.26 (-0.28, -0.24) |
|  | *P*-value | Ref. | <0.01 | <0.01 |  | Ref. | 0.01 | 0.77 |
|  | Niño 3.4 | -0.28 (-0.30, -0.25) | -0.27 (-0.30, -0.23) | -0.27 (-0.31, -0.23) |  | -0.25 (-0.29, -0.21) | -0.39 (-0.42, -0.35) | -0.30 (-0.33, -0.27) |
|  | *P*-value | Ref. | 0.74 | 0.80 |  | Ref. | <0.01 | 0.04 |
|  | ONI | -0.34 (-0.38, -0.30) | -0.32 (-0.37, -0.27) | -0.40 (-0.45, -0.34) |  | -0.38 (-0.43, -0.32) | -0.50 (-0.55, -0.45) | -0.33 (-0.37, -0.29) |
|  | *P*-value | Ref. | 0.46 | 0.10 |  | Ref. | <0.01 | 0.16 |
|  | Negative SOI | -0.15 (-0.16, -0.13) | -0.15 (-0.17, -0.12) | -0.10 (-0.13, -0.08) |  | -0.14 (-0.17, -0.12) | -0.17 (-0.19, -0.15) | -0.17 (-0.20, -0.15) |
|  | *P*-value | Ref. | 0.92 | 0.01 |  | Ref. | 0.12 | 0.07 |
|  | BEST | -0.25 (-0.27, -0.23) | -0.22 (-0.24, -0.20) | -0.24 (-0.26, -0.21) |  | -0.28 (-0.30, -0.25) | -0.29 (-0.31, -0.27) | -0.21 (-0.23, -0.19) |
|  | *P*-value | Ref. | 0.01 | 0.25 |  | Ref. | 0.66 | <0.01 |
| *Panel B. At the value of -1 for each indicator* | | | | | | | | |
| **FFB yield in 0-23 lagged months** | | | | | | | | |
|  | MEI | -1.46 (-1.62, -1.30) | -2.04 (-2.19, -1.89) | -1.77 (-1.93, -1.61) |  | -2.08 (-2.24, -1.91) | -1.89 (-2.04, -1.74) | -1.22 (-1.36, -1.07) |
|  | *P*-value | Ref. | <0.01 | 0.01 |  | Ref. | 0.10 | <0.01 |
|  | Niño 1+2 | -1.83 (-1.91, -1.75) | -1.89 (-1.98, -1.80) | -1.65 (-1.75, -1.54) |  | -1.92 (-2.02, -1.82) | -1.84 (-1.93, -1.74) | -1.45 (-1.53, -1.37) |
|  | *P*-value | Ref. | 0.33 | 0.01 |  | Ref. | 0.22 | <0.01 |
|  | Niño 3.4 | -3.46 (-3.80, -3.12) | -4.32 (-4.68, -3.97) | -3.36 (-3.73, -2.98) |  | -3.68 (-4.06, -3.29) | -4.60 (-4.95, -4.25) | -3.85 (-4.17, -3.53) |
|  | *P*-value | Ref. | <0.01 | 0.69 |  | Ref. | <0.01 | 0.49 |
|  | ONI | -4.03 (-4.45, -3.62) | -4.52 (-4.97, -4.07) | -4.35 (-4.83, -3.87) |  | -4.35 (-4.84, -3.86) | -5.46 (-5.92, -5.00) | -4.23 (-4.63, -3.83) |
|  | *P*-value | Ref. | 0.12 | 0.33 |  | Ref. | <0.01 | 0.71 |
|  | Negative SOI | -0.56 (-0.66, -0.47) | -0.69 (-0.78, -0.59) | -0.49 (-0.59, -0.39) |  | -0.78 (-0.87, -0.68) | -0.48 (-0.57, -0.39) | -0.41 (-0.50, -0.31) |
|  | *P*-value | Ref. | 0.07 | 0.28 |  | Ref. | <0.01 | <0.01 |
|  | BEST | -1.9 (-2.06, -1.73) | -2.36 (-2.54, -2.18) | -2.15 (-2.34, -1.96) |  | -3.10 (-3.29, -2.91) | -2.38 (-2.56, -2.20) | -1.48 (-1.65, -1.31) |
|  | *P*-value | Ref. | <0.01 | 0.05 |  | Ref. | <0.01 | <0.01 |
| **Average OER (%) in 0-10 lagged months** | | | | | | | | |
|  | MEI | -0.80 (-1.09, -0.52) | 0.17 (-0.11, 0.44) | -0.48 (-0.78, -0.18) |  | -0.44 (-0.79, -0.09) | -0.41 (-0.68, -0.13) | -0.14 (-0.42, 0.14) |
|  | *P*-value | Ref. | <0.01 | 0.12 |  | Ref. | 0.89 | 0.19 |
|  | Niño 1+2 | -0.25 (-0.40, -0.09) | 0.05 (-0.11, 0.22) | -0.33 (-0.50, -0.16) |  | -0.51 (-0.71, -0.30) | 0.04 (-0.13, 0.20) | -0.07 (-0.22, 0.08) |
|  | *P*-value | Ref. | 0.01 | 0.49 |  | Ref. | <0.01 | <0.01 |
|  | Niño 3.4 | -0.64 (-1.11, -0.16) | 0.15 (-0.33, 0.63) | -0.75 (-1.24, -0.26) |  | -0.48 (-1.01, 0.05) | -0.62 (-1.10, -0.15) | -0.32 (-0.78, 0.15) |
|  | *P*-value | Ref. | 0.02 | 0.74 |  | Ref. | 0.70 | 0.64 |
|  | ONI | -0.5 (-1.09, 0.08) | 0.45 (-0.14, 1.05) | -0.90 (-1.50, -0.29) |  | -0.53 (-1.18, 0.11) | -0.54 (-1.10, 0.03) | -0.04 (-0.64, 0.55) |
|  | *P*-value | Ref. | 0.02 | 0.36 |  | Ref. | 0.99 | 0.27 |
|  | Negative SOI | 0.09 (-0.16, 0.34) | 0.53 (0.28, 0.78) | 0.44 (0.19, 0.70) |  | 0.15 (-0.12, 0.42) | 0.50 (0.26, 0.74) | 0.46 (0.22, 0.70) |
|  | *P*-value | Ref. | 0.01 | 0.05 |  | Ref. | 0.05 | 0.09 |
|  | BEST | -0.18 (-0.41, 0.04) | 0.19 (-0.03, 0.42) | 0.05 (-0.19, 0.29) |  | -0.20 (-0.44, 0.03) | 0.10 (-0.11, 0.32) | 0.08 (-0.15, 0.32) |
|  | *P*-value | Ref. | 0.02 | 0.16 |  | Ref. | 0.06 | 0.09 |
| **Oil yield in 0-23 lagged months** | | | | | | | | |
|  | MEI | -0.20 (-0.23, -0.17) | -0.35 (-0.37, -0.32) | -0.32 (-0.35, -0.29) |  | -0.33 (-0.36, -0.30) | -0.3 (-0.32, -0.27) | -0.22 (-0.25, -0.19) |
|  | *P*-value | Ref. | <0.01 | <0.01 |  | Ref. | 0.13 | <0.01 |
|  | Niño 1+2 | -0.29 (-0.31, -0.28) | -0.24 (-0.25, -0.23) | -0.23 (-0.25, -0.22) |  | -0.28 (-0.29, -0.26) | -0.25 (-0.27, -0.24) | -0.22 (-0.24, -0.21) |
|  | *P*-value | Ref. | <0.01 | <0.01 |  | Ref. | 0.02 | <0.01 |
|  | Niño 3.4 | -0.68 (-0.75, -0.61) | -0.87 (-0.94, -0.80) | -0.68 (-0.76, -0.61) |  | -0.77 (-0.85, -0.69) | -0.92 (-0.99, -0.85) | -0.78 (-0.84, -0.71) |
|  | *P*-value | Ref. | <0.01 | 0.99 |  | Ref. | 0.01 | 0.95 |
|  | ONI | -0.86 (-0.94, -0.78) | -1.02 (-1.11, -0.93) | -0.95 (-1.05, -0.86) |  | -1.04 (-1.14, -0.94) | -1.18 (-1.27, -1.09) | -0.90 (-0.98, -0.82) |
|  | *P*-value | Ref. | 0.01 | 0.13 |  | Ref. | 0.04 | 0.03 |
|  | Negative SOI | -0.10 (-0.12, -0.08) | -0.14 (-0.16, -0.13) | -0.10 (-0.12, -0.08) |  | -0.16 (-0.18, -0.14) | -0.10 (-0.12, -0.08) | -0.08 (-0.10, -0.06) |
|  | *P*-value | Ref. | <0.01 | 0.92 |  | Ref. | <0.01 | <0.01 |
|  | BEST | -0.33 (-0.36, -0.29) | -0.46 (-0.49, -0.42) | -0.41 (-0.45, -0.37) |  | -0.59 (-0.63, -0.55) | -0.43 (-0.47, -0.39) | -0.28 (-0.31, -0.24) |
|  | *P*-value | Ref. | <0.01 | <0.01 |  | Ref. | <0.01 | <0.01 |

Notes: (1) Abbreviations: ENSO, El Niño Southern Oscillation; MEI, multivariate El Niño index; ONI, oceanic Niño index; SOI, Southern Oscillation Index; BEST, Bivariate ENSO Timeseries; Ref., reference group. (2) *P*-values are for the differences in effect estimates across subgroups. The association of each ENSO measure with each outcome is computed as the effect of a given value of ENSO measure relative to the reference value (set at zero). (3) For detailed definitions and data sources, please refer to Table S2.

Table S9.

**Cumulative associations between the FFB yield, average OER, and oil yield and extreme levels of ENSO exposure stratified by estates’ exposure to the monthly average soil temperature in the 0-23 lagged months.**

| **Indicator level** | | **At the value of +2 for each indicator** | | |  | **At the value of -1 for each indicator** | | |
| --- | --- | --- | --- | --- | --- | --- | --- | --- |
| **Subgroup** | | **First tertile** | **Second tertile** | **Third tertile** |  | **First tertile** | **Second tertile** | **Third tertile** |
| **FFB yield in 0-23 lagged months** | | | | | | | | |
|  | MEI | -2.16 (-2.36, -1.97) | -2.77 (-2.97, -2.58) | -2.10 (-2.32, -1.88) |  | -1.56 (-1.70, -1.41) | -1.79 (-1.93, -1.64) | -2.10 (-2.26, -1.93) |
|  | *P*-value | Ref. | <0.01 | 0.65 |  | Ref. | 0.03 | <0.01 |
|  | Niño 1+2 | -0.59 (-0.69, -0.48) | -1.44 (-1.54, -1.34) | -1.24 (-1.36, -1.12) |  | -1.71 (-1.79, -1.64) | -1.72 (-1.80, -1.65) | -1.82 (-1.93, -1.70) |
|  | *P*-value | Ref. | <0.01 | <0.01 |  | Ref. | 0.84 | 0.15 |
|  | Niño 3.4 | -1.16 (-1.31, -1.01) | -1.71 (-1.86, -1.56) | -1.61 (-1.81, -1.41) |  | -4.20 (-4.53, -3.86) | -3.90 (-4.22, -3.57) | -3.98 (-4.36, -3.60) |
|  | *P*-value | Ref. | <0.01 | <0.01 |  | Ref. | 0.21 | 0.41 |
|  | ONI | -0.94 (-1.16, -0.72) | -1.98 (-2.20, -1.76) | -1.95 (-2.23, -1.68) |  | -4.28 (-4.70, -3.86) | -4.66 (-5.07, -4.25) | -4.82 (-5.31, -4.33) |
|  | *P*-value | Ref. | <0.01 | <0.01 |  | Ref. | 0.20 | 0.10 |
|  | Negative SOI | -0.81 (-0.93, -0.70) | -0.85 (-0.97, -0.73) | -0.62 (-0.76, -0.48) |  | -0.61 (-0.70, -0.52) | -0.61 (-0.70, -0.52) | -0.57 (-0.67, -0.47) |
|  | *P*-value | Ref. | 0.65 | 0.04 |  | Ref. | 0.94 | 0.57 |
|  | BEST | -0.83 (-0.93, -0.72) | -1.33 (-1.43, -1.22) | -1.37 (-1.49, -1.25) |  | -2.03 (-2.20, -1.87) | -2.08 (-2.25, -1.91) | -2.58 (-2.77, -2.38) |
|  | *P*-value | Ref. | <0.01 | <0.01 |  | Ref. | 0.70 | <0.01 |
| **Average OER (%) in 0-10 lagged months** | | | | | | | | |
|  | MEI | 0.01 (-0.51, 0.53) | -0.18 (-0.72, 0.35) | -0.30 (-0.82, 0.21) |  | -0.45 (-0.72, -0.17) | -0.09 (-0.36, 0.18) | -0.45 (-0.76, -0.14) |
|  | *P*-value | Ref. | 0.60 | 0.40 |  | Ref. | 0.07 | 0.97 |
|  | Niño 1+2 | 0.07 (-0.25, 0.40) | -0.22 (-0.54, 0.10) | 0.09 (-0.26, 0.43) |  | -0.14 (-0.29, 0.01) | -0.20 (-0.35, -0.05) | -0.14 (-0.33, 0.05) |
|  | *P*-value | Ref. | 0.21 | 0.96 |  | Ref. | 0.58 | 0.97 |
|  | Niño 3.4 | -0.06 (-0.48, 0.36) | 0.11 (-0.28, 0.50) | -0.01 (-0.43, 0.41) |  | -0.20 (-0.65, 0.24) | -0.22 (-0.66, 0.21) | -0.87 (-1.41, -0.33) |
|  | *P*-value | Ref. | 0.55 | 0.86 |  | Ref. | 0.95 | 0.06 |
|  | ONI | -0.12 (-0.65, 0.41) | -0.07 (-0.57, 0.42) | -0.34 (-0.89, 0.22) |  | -0.08 (-0.64, 0.48) | 0.03 (-0.51, 0.57) | -1.06 (-1.70, -0.41) |
|  | *P*-value | Ref. | 0.89 | 0.58 |  | Ref. | 0.79 | 0.03 |
|  | Negative SOI | -0.20 (-0.45, 0.04) | -0.38 (-0.63, -0.14) | -0.18 (-0.43, 0.06) |  | 0.25 (0.01, 0.49) | 0.55 (0.31, 0.78) | 0.27 (0.01, 0.53) |
|  | *P*-value | Ref. | 0.31 | 0.90 |  | Ref. | 0.09 | 0.92 |
|  | BEST | -0.13 (-0.43, 0.17) | -0.23 (-0.52, 0.06) | -0.17 (-0.46, 0.12) |  | -0.02 (-0.24, 0.20) | 0.16 (-0.06, 0.38) | -0.14 (-0.39, 0.11) |
|  | *P*-value | Ref. | 0.65 | 0.85 |  | Ref. | 0.25 | 0.47 |
| **Oil yield in 0-23 lagged months** | | | | | | | | |
|  | MEI | -0.32 (-0.35, -0.28) | -0.51 (-0.55, -0.47) | -0.35 (-0.39, -0.30) |  | -0.21 (-0.24, -0.18) | -0.33 (-0.35, -0.30) | -0.35 (-0.38, -0.32) |
|  | *P*-value | Ref. | <0.01 | 0.30 |  | Ref. | <0.01 | <0.01 |
|  | Niño 1+2 | -0.17 (-0.19, -0.15) | -0.31 (-0.33, -0.29) | -0.25 (-0.27, -0.22) |  | -0.23 (-0.25, -0.22) | -0.29 (-0.30, -0.27) | -0.23 (-0.25, -0.22) |
|  | *P*-value | Ref. | <0.01 | <0.01 |  | Ref. | <0.01 | 0.95 |
|  | Niño 3.4 | -0.22 (-0.25, -0.19) | -0.35 (-0.38, -0.32) | -0.31 (-0.35, -0.26) |  | -0.81 (-0.88, -0.74) | -0.82 (-0.89, -0.75) | -0.78 (-0.86, -0.71) |
|  | *P*-value | Ref. | <0.01 | <0.01 |  | Ref. | 0.88 | 0.57 |
|  | ONI | -0.23 (-0.28, -0.19) | -0.44 (-0.49, -0.40) | -0.42 (-0.48, -0.37) |  | -0.92 (-1.01, -0.84) | -1.05 (-1.13, -0.96) | -1.04 (-1.14, -0.95) |
|  | *P*-value | Ref. | <0.01 | <0.01 |  | Ref. | 0.04 | 0.06 |
|  | Negative SOI | -0.18 (-0.20, -0.16) | -0.14 (-0.16, -0.12) | -0.11 (-0.13, -0.08) |  | -0.11 (-0.13, -0.10) | -0.13 (-0.15, -0.11) | -0.11 (-0.13, -0.09) |
|  | *P*-value | Ref. | <0.01 | <0.01 |  | Ref. | 0.16 | 1.00 |
|  | BEST | -0.20 (-0.22, -0.18) | -0.25 (-0.27, -0.23) | -0.27 (-0.29, -0.24) |  | -0.35 (-0.39, -0.32) | -0.40 (-0.44, -0.37) | -0.48 (-0.52, -0.44) |
|  | *P*-value | Ref. | <0.01 | <0.01 |  | Ref. | 0.06 | <0.01 |

Notes: (1) Abbreviations: ENSO, El Niño Southern Oscillation; MEI, multivariate El Niño index; ONI, oceanic Niño index; SOI, Southern Oscillation Index; BEST, Bivariate ENSO Timeseries; Ref., reference group. (2) *P*-values are for the differences in effect estimates across subgroups. The association of each ENSO measure with each outcome is computed as the effect of a given value of ENSO measure relative to the reference value (set at zero). (3) For detailed definitions and data sources, please refer to Extended Data Table S2.
